# Estimating eviction prevalence across the United States

**DOI:** 10.1073/pnas.2116169119

**Published:** 2022-05-16

**Authors:** Ashley Gromis, Ian Fellows, James R. Hendrickson, Lavar Edmonds, Lillian Leung, Adam Porton, Matthew Desmond

**Affiliations:** ^a^Department of Sociology, Princeton University, Princeton, NJ 08540;; ^b^Fellows Statistics, San Diego, CA 92107

**Keywords:** eviction, residential inequality, housing policy

## Abstract

Drawing on 99.9 million court records, we construct national estimates of the annual prevalence of eviction filings and households threatened with eviction in the United States. Using Bayesian hierarchical modeling, we reconcile data from multiple sources to create comprehensive estimates permitting comparisons of eviction filing risk within and between states. This method indicates that relying solely on court-issued data undercounts eviction filings by approximately 1 million cases a year due to omission of counties for which these data cannot be obtained. In an average year between 2000 and 2018, landlords filed more than 3.6 million eviction cases, resulting in almost 7% of renting households facing an eviction lawsuit. During this time, the number of eviction filings nationally increased by 21.5%; however, an expanding renter population has outpaced the growth in filings, resulting in declining filing rates in recent years. Nationwide data reveal stark disparities in eviction filing rates between states that are not explained by variation in sociodemographic composition. Rather, regression discontinuity models indicate a robust association between a simple housing policy—requiring landlords to provide notice to tenants prior to filing an eviction case for nonpayment of rent—and the county-level eviction filing rate, demonstrating that larger structural factors, including state-level landlord–tenant law, could play an important role in shaping risk of receiving an eviction filing. We make aggregated data publicly available to serve as a tool for researchers, policymakers, and members of the public to examine the prevalence, causes, and consequences of eviction lawsuits.

## Significance

Several negative effects of forced displacement have been well documented, yet we lack reliable measurement of eviction risk in the national perspective. This prevents accurate estimations of the scope and geography of the problem as well as evaluations of policies to reduce housing loss. We construct a nationwide database of eviction filings in the United States. Doing so reveals that 2.7 million households, on average, are threatened with eviction each year; that the highest eviction filing rates are not concentrated solely in high-cost urban areas; and that state-level housing policies are strongly associated with county-level eviction filing risk. These data facilitate an expanded research agenda on the causes and consequences of eviction lawsuits in the United States.

Court-ordered eviction and displacement due to eviction are primary causes of homelessness (1–3) and have long-term effects on material hardship and health (4, 5). Beyond the immediate loss of housing, these events inhibit long-term residential security and neighborhood choice (6–8). Eviction cases are filed by landlords in local courts, most frequently for nonpayment of rent. These filings are recorded in tenants’ housing histories, often regardless of the case outcome, making these actions visible to landlords on tenant screening reports and limiting access to future rental housing (9). Policymakers have recognized the disruptive effects of housing loss, most recently by issuing eviction moratoriums during the COVID-19 pandemic (10).

Yet there exist no comprehensive estimates of the annual frequency of eviction lawsuits nationwide. Targeted surveys have identified the prevalence of eviction-related displacement in cities (7, 11, 12); however, differences in how eviction is measured hinder direct comparisons of high- and low-displacement areas. Surveys covering larger geographical areas (e.g., state, nation) are expensive and frequently underrepresent hard-to-reach populations at risk for eviction, including economically disadvantaged renters and those with unstable housing (13, 14). The 2017 American Housing Survey (AHS) asked renter households about receiving an eviction notice in the previous 3 mo, which provided an important point-in-time national estimate of households at risk for eviction, but could not measure the total volume of eviction filings or multiple eviction notices issued against the same household. The AHS sampling frame also limits the comparisons that can be made across states and outside of metropolitan areas. Court records of eviction case filings provide an alternative source of passively collected data (15) to study eviction filing and displacement prevalence (6, 16, 17); however, lack of centralized collection and standardization of these records restrict their geographic scope and comparability.

Local studies have identified sociodemographic characteristics associated with eviction filings and displacement at the individual and neighborhood levels (18, 19) but have been unable to examine how underlying risk of eviction varies geographically. Ignoring differences in rental environments created by state-level landlord–tenant policy (20) may overemphasize microlevel determinants while obscuring legislative regimes responsible for large-scale disparities in eviction prevalence.

Identifying the drivers and consequences of housing loss requires assembling large-scale data infrastructure that can provide longitudinally and geographically consistent measurements of eviction. To achieve this, we compiled, standardized, and validated 99.9 million court records to construct a national database of eviction lawsuits in the United States. We incorporated these data into a Bayesian hierarchical model to produce a set of comprehensive estimates of eviction filings and households threatened with eviction from 2000 to 2018. By making these data publicly available, we hope to help advance research on the causes and consequences of residential instability.

## Materials and Methods

Creating a data infrastructure to produce consistent and comprehensive estimates of eviction prevalence required four steps:

1) Defining the measurement of eviction
2) Compiling public eviction records
3) Validating the coverage of eviction cases in court records
4) Bayesian estimation of eviction prevalence in areas without validated court records.

### Definition of Eviction.

Discrepancies in how eviction is defined can produce conflicting measurements, requiring a precise definition to produce reliable and comparable estimates of prevalence (1). We created two measures of annual eviction prevalence:

1) Eviction case filings. The filing of an eviction lawsuit in court represents the first action in the legal eviction process. There are many possible outcomes to these filings: Tenants may leave the property without contesting the case; the court may order tenants to vacate the property or pay past-due rent; or the landlord and tenants may resolve the dispute with the tenants remaining at the property. The number of eviction filings represents the total volume of eviction cases processed by the legal system. Eviction filings often generate multiple records associated with different legal events related to the case (e.g., filing, judgment). An accurate count of eviction filings requires that individual records be aggregated to the case level.
2) Households threatened with eviction. Some households receive multiple filings threatening eviction in a given year, particularly in areas where landlords use the courts to enforce rent collection (21). The number of unique households receiving at least one eviction filing represents how many households have been threatened with displacement each year.

### Collection of Eviction Records.

We intended to build a comprehensive national database of eviction cases to create annual measures of eviction filings and households threatened with eviction. We requested all available public electronic records of eviction filings directly from courts in all 50 states and the District of Columbia (DC). We received 26.7 million individual records representing 17.7 million cases in 19 states and eight additional counties. Data coverage varied significantly across these states and counties over time (*SI Appendix*, Table S1). Many court systems lacked consistent digitization of case records prior to 2000 or only implemented centralized case management systems in more recent years, preventing comprehensive collection of records across years. Other states have yet to implement centralized case management systems or have legal barriers to making bulk records requests, preventing collection of electronic court records in any year.

The inability to assemble a comprehensive set of eviction filings annually through records requests required us to collect two additional sources of eviction case data. First, we made secondary requests for aggregated annual counts of eviction filings by county, typically the smallest areal unit for which states tabulate filings, from all states and DC for the 2000 to 2018 period. We collected 31,845 aggregated filing counts representing 44.9 million cases from 2,204 counties across 46 states (*SI Appendix*, Table S2). These aggregated counts did not include case-specific information needed to calculate the number of unique households threatened with eviction but allowed us to measure filings in areas where individual electronic records were not available. We refer to these first two sources of data as “court-issued data” as they were provided directly by the courts. Together, the court-issued data covered 2,272 counties across 48 states and DC for at least 1 y in the 2000 to 2018 period (*SI Appendix*, Fig. S1).

Second, we purchased 73.2 million individual eviction records covering 48 states and DC from LexisNexis Risk Solutions (*n* = 40.7 million residential cases filed in the 2000 to 2018 period). These proprietary data provided an important measure of filings and households threatened with eviction in areas without court-issued data, because, in addition to electronic records requests, they perform manual collection of case information from files only accessible in person at courthouses. While this creates electronic case data in areas where it otherwise would not exist, the data may be incomplete in courts that restrict access to records or have other barriers to consistent collection (e.g., off-site record storage, very high filing volume).

We produced annual county-level counts of case filings and households threatened with eviction across these three data sources to create comprehensive estimates of our eviction measures. For court-issued aggregated filing counts, the data directly provided annual measures of filings. For the individual court-issued and proprietary records, we grouped filings by households using probabilistic (inexact) matching across tenant names and addresses listed in the individual records and excluded filings against commercial properties. We aggregated filings and the number of unique households represented in those filings by the county where the case was filed and the year from the earliest-dated record associated with the case. Full details of the collection, standardization, and aggregation of the eviction data are available in *SI Appendix*, section 1.

### Validation of Court-Issued Data.

Although the court-issued data would not be expected to face the same collection challenges as proprietary data, staged implementation of new case management systems, changes in recordkeeping, and inconsistent reporting by local courts can result in incomplete representation of eviction filings. To identify these instances, we validated the court-issued data in two ways. First, we compared filing counts generated from court-issued individual records with court-issued aggregate filing counts in counties for which we obtained both sources of data. Second, we identified substantial fluctuations in case filings across years in the same county. Full details of these validation methods are included in *SI Appendix*, section 2. We excluded court-issued counts of filings that did not appear to be reliable indicators of the true case filing volume (*SI Appendix*, Table S3).

Despite including 50,738,468 eviction filings—an average of 2.6 million annually—the court-issued data covered only 66.8% of renting households (*SI Appendix*, Table S4). To produce comprehensive estimates, we needed to reconcile the proprietary data, which do not contain every filing, with the validated court-issued filing counts. Additionally, a small number of counties did not have validated court-issued or proprietary data (*SI Appendix*, Fig. S1).

### Bayesian Estimation of Eviction Filings and Households Threatened with Eviction.

The inability to collect a complete set of court-issued eviction records annually required that we develop a methodological strategy to produce population-level estimates by reconciling multiple sources of overlapping—but incomplete—data (22). To do this, we incorporated the court-issued and proprietary data into a Bayesian hierarchical model to estimate annual case filings for all counties from 2000 to 2018, including those missing court-issued data. We specified the probability model of logged court-issued filing counts as a function of county demographic, court, and case characteristics, hierarchical variation at the county, state, and region levels, and yearly variation. To estimate how well the proprietary filing counts corresponded to the court-issued data, we specified a secondary probability model, which modeled the proprietary filing counts as a function of the court-issued filing counts and record collection characteristics (*SI Appendix*, section 3).

Annual eviction filing counts could be generated in two ways. If we had validated court-issued data for a county year, we used these observed filing counts. If we lacked those data, we generated estimates using the posterior predictive distribution from the Bayesian model. This allowed us to produce estimates of filing volume in county years that would have otherwise been partially or completely missing from the national estimates. We calculated eviction filing rates by dividing the number of filings by total renting households in each county year.

We leveraged the Bayesian model again to estimate the number of households threatened with eviction in each county year by incorporating annual counts of distinct households represented in case filings from the individual-level court-issued and proprietary eviction records (*SI Appendix*, section 4). This allowed us to preserve important differences in the rates of repeated filings against the same households across states, discussed in more detail below. We calculated rates of households threatened with eviction by dividing the number of distinct households named in the eviction filings by the total renting households in each county year. We calculated the percent of cases representing repeated filings against the same households by finding the difference between the total number of filings and the number of filings representing distinct households and then dividing by the total number of filings. Aggregated court-issued and proprietary filing counts used to produce these estimates, as well as the full set of Bayesian posterior estimates, are posted publicly (23). We display the correspondence between court-issued data and Bayesian posterior estimates in *SI Appendix*, section 5.1.

## Results

**Fig. 1.**
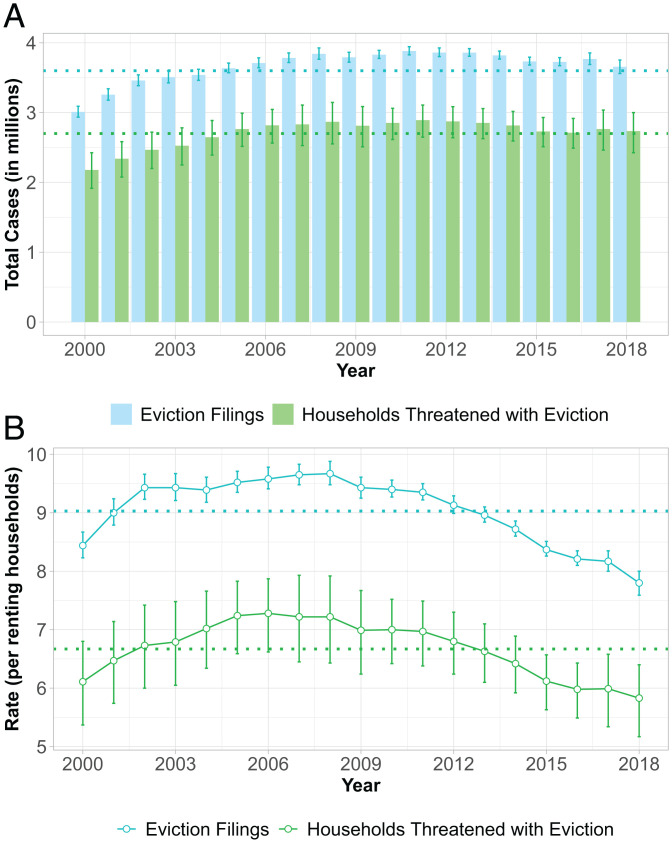
Prevalence of eviction filings and households threatened with eviction in the United States, 2000 to 2018. (*A*) Annual eviction case filings and households that received at least one eviction filing and (*B*) rates of filings and households threatened with eviction per renting households. *n* = 3,143 counties annually; 59,717 total county years. Error bars show 95% credible interval. Respective averages across years are shown by dotted lines. Number of renting households are estimated using linear interpolation between 2000 and 2010 censuses and 2016 Environmental Systems Research Institute (ESRI) Business Analyst. Similar trends were observed when using 1-y American Community Survey tenure estimates (*SI Appendix*, Fig. S2).

Between 2000 and 2018, 69.7 million eviction cases were filed in the United States. This is an average of more than 3.6 million filings annually (Fig. 1*A*), representing approximately nine eviction cases per 100 renting households (Fig. 1*B*). Relying solely on data available for collection directly from courts without incorporating estimates from the Bayesian models would have omitted counties representing 34% of renting households, underestimating annual filings by ∼1 million cases, on average. The predictions from the Bayesian posterior distribution were robust to alternative model specifications, including the exclusion of the proprietary data as a secondary measurement of eviction filing volume (*SI Appendix*, section 5.2).

### Longitudinal Trends in Eviction Filing Prevalence.

The number of eviction filings increased by 21.5% between 2000 and 2018, from 3,009,832 to 3,656,428 cases (Fig. 1*A*); however, the eviction filing rate (the ratio of filings to renting households) followed a modest curvilinear trend, increasing between 2000 and 2008 before decreasing in recent years (Fig. 1*B*). The filing rate peak coincided with the Great Recession, when economic hardship may have increased rent nonpayment. Households threatened with eviction demonstrated similar longitudinal trends. Approximately 2.7 million households received an eviction filing annually, or just fewer than 7 in 100 renting households, on average. In 2011, 2.9 million households received at least one eviction filing, comparable to the number of foreclosure starts at the height of the foreclosure crisis in 2010 (24). Our estimated rate of households threatened with eviction in 2017 (6.0%) is similar to the rough calculation of households receiving an eviction notice obtained from the 2017 AHS (1.8% in a 3-mo period suggests 7.3% in a 12-mo period) (25). The lower rate in our data likely reflects that some of the AHS households who received an eviction notice in the initial 3-mo period received a notice again in subsequent 3-mo periods within the same year.

**Fig. 2.**
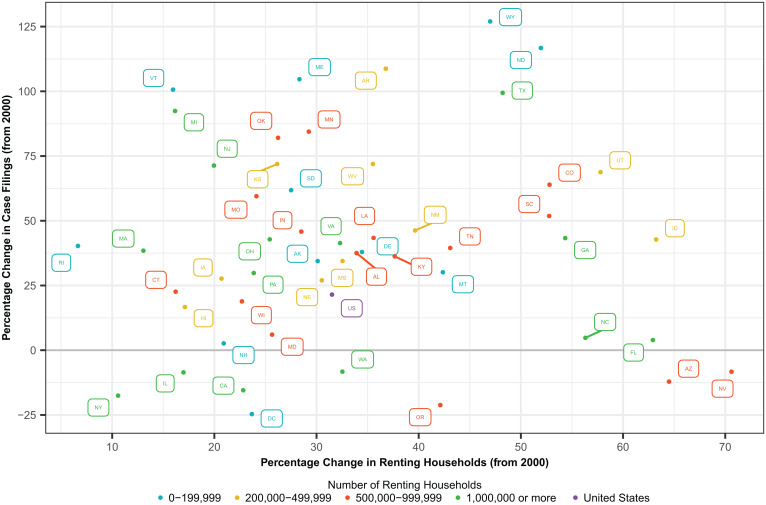
Percentage change in eviction filings and number of renting households by state, 2018. Percentage change in cases filed and renting households calculated in comparison to their respective numbers in 2000. The data point for the United States (shown in purple) reflects the longitudinal trend in Fig. 1*A*; filing counts increased 21.5% from 2000 to 2018, but the number of renting households increased 31.5% during the same period (*SI Appendix*, Fig. S3). This explains why the total number of filings has increased between 2000 and 2018 but the 2018 filing rate is lower than that reported for 2000 (Fig. 1*B*). For demonstration of these changes across the 2000 to 2018 period, annual case filings are plotted against number of renting households for the United States in *SI Appendix*, Fig. S4.

The seemingly contradictory trend of an increasing number of eviction filings coupled with a recent decline in rates of filings and households threatened with eviction is attributable to increases in renting households during the same period. Since 2000, the percentage increase in renting households has outpaced that of eviction filings. Although this trend was not present uniformly in all states, it was observed in many states with large shares of renting households, including New York and California, where the number of case filings decreased from 2000 to 2018 (Fig. 2).

Given rising housing costs in the United States (*SI Appendix*, Fig. S5), why have the number of eviction filings not kept pace with increases in renting households? One possible explanation is that the demographic profile of renters is changing, with increasing percentages of renters who are older (*SI Appendix*, Fig. S6) and wealthier (*SI Appendix*, Fig. S7). These households contribute to the pool of renting households but may not substantially increase the number of eviction filings, leading to declines in filing rates even as the absolute number of filings has increased. State-level economic trends, housing market conditions, and landlord–tenant policy environments may also create disparate trajectories of growth (or decline) in filing prevalence over time, underscoring the importance of creating comparable metrics across states to assess how these factors shape local and national eviction filing trends.

### Spatial Concentration of Eviction Filings.

**Fig. 3.**
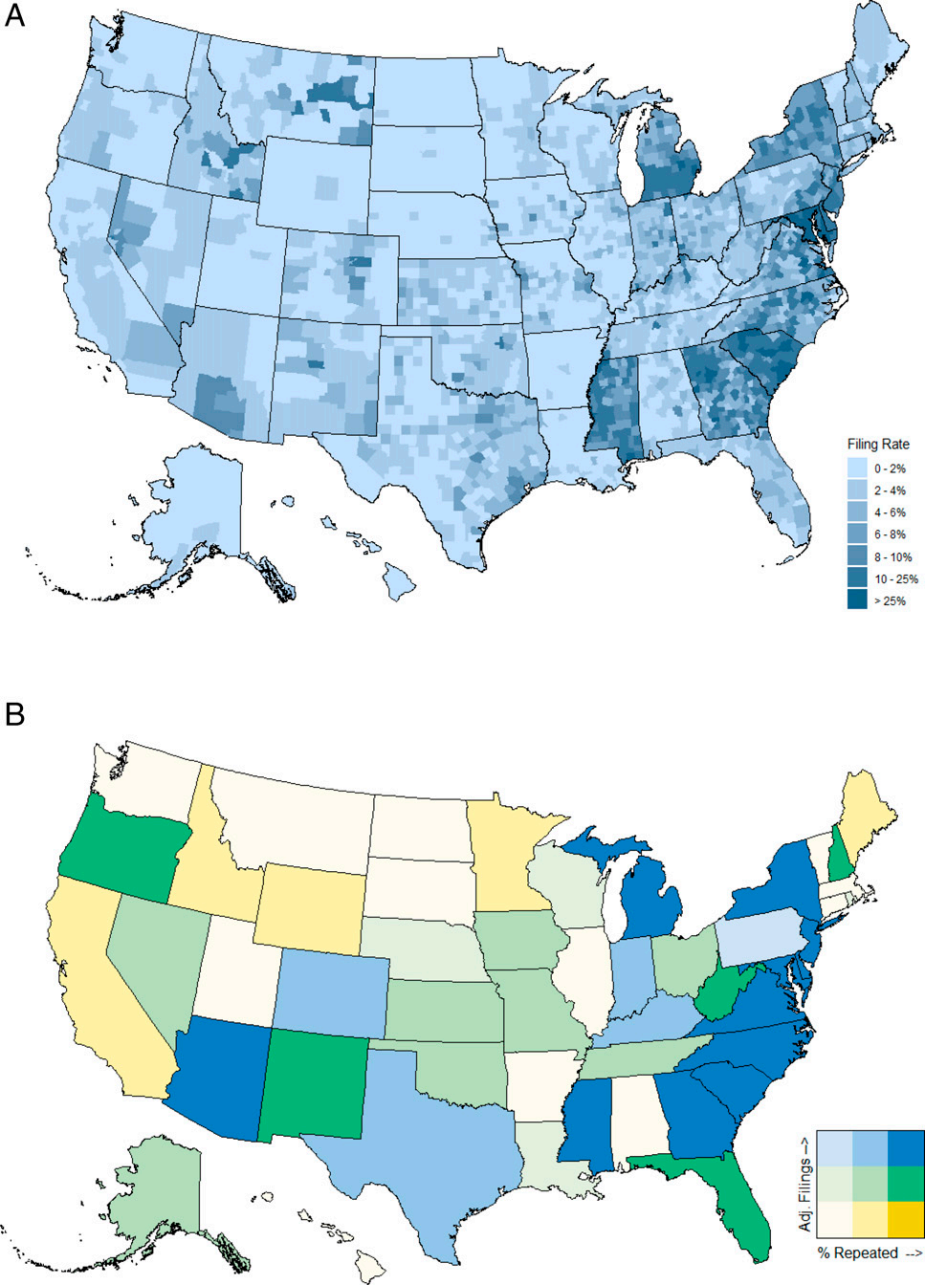
Geography of eviction filings, 2018. (*A*) County-level filing rates demonstrated noticeable disparities across state lines, with the highest rates clustered in the Southeast. (*B*) Most states are located on the diagonal of the bivariate association between filings adjusted for sociodemographic conditions and repeated filings against the same household (shown by tertiles), demonstrating the strong association between repeated filings and increased state-level risk of filings. Adjusted filing counts for a comparable county in each state are shown in *SI Appendix*, Fig. S10 and percent repeated filings against households is shown in *SI Appendix*, Fig. S11.

Despite the prominence of large, high-cost metro areas in discussions of affordable housing in the United States, the highest eviction filing rates were clustered in the relatively low-cost region of the Southeast (Fig. 3*A*). Southern states consistently showed the highest filing rates throughout the 2000 to 2018 period (*SI Appendix*, Fig. S8). Recognizing differences in filing rates across states is a departure from many previous studies of eviction risk (of both filings and displacement due to eviction), which tend to focus on prevalence in specific metropolitan areas and associations of these actions with poverty rates, high rent costs, or rent burden (the ratio of rent costs to household income). Population, median rent, and rent burden showed only modest, positive bivariate associations with county-level filing rates in our data, while poverty rates showed no bivariate association (*SI Appendix*, Fig. S9). Understanding eviction filing risk requires examination of sociodemographic and other potential correlates within the larger state context.

Between-state disparities in eviction filing rates could reflect larger concentrations of sociodemographic characteristics associated with increased aggregate risk of eviction filings, including the number of renting households, household density, share of African American population, median income, and median rent (*SI Appendix*, Table S5). To investigate this, we used the Bayesian model to predict the expected number of eviction filings in a hypothetical county for each state with all demographic and county characteristics set equal to their overall population means. This did not eliminate filing disparities. Instead, counties in many southeastern states were still expected to have significantly more filings than demographically identical counties in other regions (Fig. 3*B*). Continued filing disparities showed a clear association with the percent of eviction cases representing repeated filings against the same households. States exhibited very different rates of repeated filings against the same households (*SI Appendix*, Fig. S11) and state-level filing rates were highly correlated over time (*SI Appendix*, Fig. S12), suggesting that differences in state-level landlord–tenant policy influence how frequently landlords file eviction lawsuits, independent of sociodemographic characteristics or economic shocks.

### State-Level Landlord–Tenant Policy.

States have different laws that govern the filing and adjudication of eviction lawsuits. Some states charge property owners several hundred dollars to file an eviction case; others charge much less (*SI Appendix*, Table S6). Landlord–tenant laws can also dictate how much notice a landlord is required to provide tenants before filing for eviction for nonpayment of rent; some states require landlords to provide tenants with a few days’ notice, others require as much as a 14-d notice, while still others require no notice at all. The required notice period usually corresponds to the minimum number of days a tenant can be late on the rent payment before risking incurring an eviction lawsuit.

To illustrate how notice requirements shape risk of receiving an eviction filing, consider Maryland, which reported a seemingly impossibly high filing rate of 69.6% in 2018. In Maryland, eviction lawsuits can be filed immediately following nonpayment of rent for relatively low cost ($15 to $25) (*SI Appendix*, Table S6); no prior notice is required from the landlord (1, 26). If tenants promptly pay the rent due (plus any additional fees), they are able to remain in the residence (27). This policy environment creates an incentive for landlords to enforce rent collection by using filings as a threat of eviction (21). Repeated filings against the same households constituted 57.4% of all cases in Maryland, demonstrating that many tenants face recurring threats of displacement while ultimately continuing to make rent payments and remaining at a property. We discuss Maryland eviction filings and how they affect estimates of national filing rates in more detail in *SI Appendix*, section 5.3.

To assess the association between eviction notice requirements and filing rates across states, we leveraged data from the state-level Eviction Law Database released by the Legal Services Corporation (28). The database included an indicator for whether a state required landlords to provide notice to tenants before filing an eviction case for nonpayment of rent and, if so, the minimum number of days’ notice required. It is difficult to disentangle the association between a state-level policy and eviction filing rates for two reasons. First, specific policies can be correlated with other aspects of the landlord–tenant legal environment or unmeasured socioeconomic characteristics that may also be associated with eviction filings. Second, many policies, including eviction notice requirements, tend to be stable over time (*SI Appendix*, Table S7), which makes it difficult to isolate policies when using fixed effects to control for other time-invariant conditions in states that may be associated with eviction filing rates (29).

In light of these challenges, we used a regression discontinuity design to examine the association between eviction notice requirements and filing rates in core-based statistical areas (CBSAs) that crossed state lines. We included fixed effects at the CBSA level, as the boundaries of these areas are designed to capture a core urban area and the surrounding counties that are socioeconomically tied to it. CBSA-level fixed effects helped account for unmeasured sociodemographic and economic characteristics in the local area while allowing policies to vary across state borders. We restricted our sample to counties with validated court-issued filing counts to ensure that associations between notice requirements and filing rates were not inflated due to correlations with any characteristics used to estimate filing counts in the Bayesian models. Full details of the models are provided in *SI Appendix*, section 6. Counties included in the analyses are shown in *SI Appendix*, Fig. S13 and CBSAs are listed in *SI Appendix*, Table S8.

**Fig. 4.**
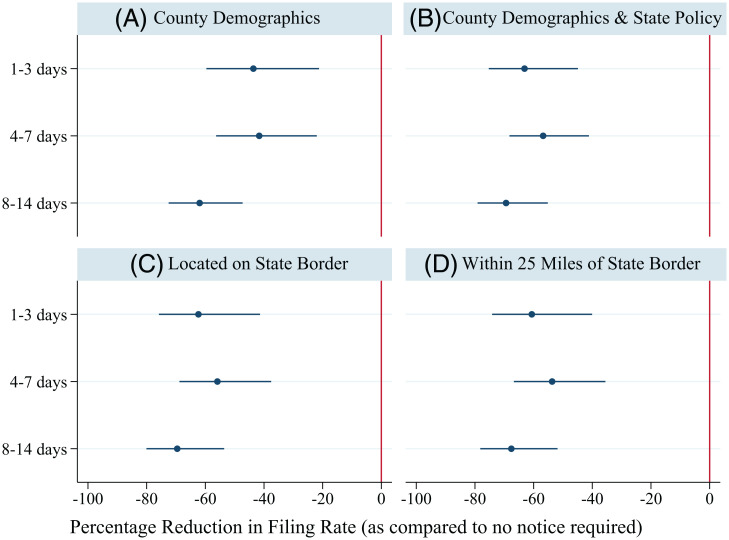
Percentage reduction in eviction filing rate associated with length of notice required before eviction filing for nonpayment of rent. (*A*) The first model (“County Demographics”) includes the county-level sociodemographic covariates used in the Bayesian model to estimate case filings (*n* = 3,074 county years). (*B*) The second model (“County Demographics and State Policy”) includes the county-level covariates plus three additional controls for state-level policy environment (*n* = 3,074 county years). (*C*) The third model (“Located on the State Border”) includes the county- and state-level covariates, but restricts the sample to counties located directly on the state border (*n* = 2,329 county years). (*D*) The fourth model (“Within 25 Miles of State Border”) includes the county- and state-level covariates, but restricts the sample to counties with population centers located within 25 miles of the state border (*n* = 2,551 county years). Coefficients used to generate the figure are shown in *SI Appendix*, Table S9.

We found a negative association between state-level requirements for providing notice prior to filing an eviction case for nonpayment of rent and county-level filing rates (Fig. 4). The association was significant regardless of the length of the notice period required (as compared to not requiring notice). The findings were robust to the inclusion of county-level demographic and state-level policy controls and sample restrictions to minimize effects of unmeasured socioeconomic conditions in the local area. Requiring even a relatively short period of notice (1 to 3 d) was associated with a 43.6% reduction in the eviction filing rate, holding constant county-level household density, percentage renting households, share of African American population, median income, median rent, and unemployment rate (Fig. 4*A*). Adding additional controls for state-level requirements for just-cause eviction, required case filing fees, and an indicator for type of landlord–tenant filings did not alter these findings; requiring landlords to provide 1 to 3 days’ notice before filing a case, as opposed to not requiring notice, was associated with a 63.1% reduction in annual filing rate (Fig. 4*B*). Associations remained similar when restricting the sample to counties located directly along the state border (Fig. 4*C*) or with population centers located within 25 miles of the state border (30) (Fig. 4*D*).

The magnitude of this association reflects the significantly higher filing rates in states that do not require eviction notices before filing against tenants for rent nonpayment. For example, Camden County, New Jersey, which does not require an eviction notice prior to filing nonpayment cases, had a filing rate of 16.1% in 2018. Directly across the Delaware River, Philadelphia County, Pennsylvania, which requires landlords to provide a minimum of 10 days’ notice prior to eviction filings for nonpayment, had a filing rate of 7.7% in the same year. Despite Philadelphia County’s higher household density and larger share of African American population—both characteristics associated with higher filing rates—its filing rate was 52.4% lower than that of Camden County. Although the models presented here do not identify causal effects of eviction notice requirements on filing rates, the results provide evidence that state-level landlord–tenant policies may shape risk of receiving an eviction filing in important ways that are not captured in local prevalence of past-due rent or other sociodemographic conditions.

## Discussion

The data presented here provide nationally comprehensive estimates of the annual prevalence of and geographical variation in eviction filings and households threatened with eviction in the United States. This work makes several important contributions. First, we quantified the prevalence of eviction filings in a national perspective, showing that 2.7 million households, on average, are threatened with eviction each year. We also demonstrated that eviction filings are widespread throughout the country, extending beyond high-cost, large metropolitan, and gentrifying urban neighborhoods, which are disproportionately represented in policy discussions about affordable housing.

Second, we developed and have made public a large dataset that expands opportunities to analyze eviction lawsuits at the national, state, and local levels. Our methods and statistical code can be replicated in future years to expand the database and estimates of eviction filings. Studies that identify the causes and consequences of residential instability, as well as those that evaluate policies designed to reduce eviction prevalence, require the reliable, reproducible investigation of eviction filings and households threatened with eviction across time and space facilitated by these data.

Third, these data revealed substantial between-state disparities in the frequency of eviction filings, controlling for sociodemographic characteristics. Regression discontinuity models suggested a strong, robust association between a simple housing policy—requiring landlords to provide notice to tenants prior to filing an eviction case for nonpayment of rent—and the county-level eviction filing rate. This association could only be revealed after the construction of a standardized eviction database covering the entire nation; failure to interrogate the role of state-level landlord–tenant law in shaping eviction filing rates or likelihood of being threatened with an eviction lawsuit misses important opportunities to develop evidence-based policies and interventions that reduce displacement risk.

Fourth, the methodological strategy of reconciling primary (court issued) and secondary (proprietary) data presented here provides a means of producing population-level estimates when collecting complete administrative records or representative survey data are costly or unfeasible. It can be adapted to produce innovative, comparable, and more accurate measurement strategies to investigate long-standing questions about drivers of the reproduction of poverty and inequality (31, 32).

### Limitations.

Along with these contributions, this study has some important limitations. First, public court records represent formal, legal eviction actions; estimation of the prevalence of informal eviction, in which landlords use means outside of the court system to force tenants to vacate rental properties (33), is beyond the scope of this work. Formal eviction filings are permanently recorded in tenants’ housing histories (8) and may create additional legal and financial burdens relative to informal evictions, rendering them more consequential to long-term residential instability and access to housing. Second, court records do not capture whether a tenant ultimately vacated the property because of the eviction filing. Some court records contain judgments issued on a case, including whether possession of the property was restored to the landlord, which represents a more immediate threat of displacement than an eviction filing. However, judgement information was not captured consistently enough in our data to estimate the prevalence of eviction-related judgments nationwide (*SI Appendix*, section 7). Identifying the number of households that vacate their residence following an eviction filing requires matching court records with supplementary data recording address changes or other evidence of residential moves; this is an important avenue for future research to quantify the prevalence of (and potential geographical variation in) displacement due to eviction lawsuits.

### Conclusions.

Eviction lawsuits are an important, but historically neglected, measure of housing insecurity. Like studies of homelessness, measurement limitations have prevented informed action designed to address residential instability (34). By constructing a national, longitudinal dataset of eviction filings in the United States, we hope to expand the opportunity for a new, collective research agenda. Now, researchers, local officials, and community members across the United States have access to information about local filing prevalence and the ability to compare that local prevalence to other counties within and across states. This opens avenues for academic research on the causes and consequences of eviction lawsuits and empowers stakeholders to use the data while crafting effective policy solutions. Using these data, this study challenges widespread presumptions that risk of receiving an eviction filing is a straightforward reflection of socioeconomic disadvantage (i.e., unpaid rent, poverty) and that it primarily affects households in high-poverty or high-rent neighborhoods. These data reveal that additional research is needed on how macrolevel policies and other political factors, such as the existence and enforcement of fair housing laws, policies governing legal grounds for eviction filings, or right to legal counsel, affect the prevalence of eviction lawsuits or relative disparities in these filings across communities by race/ethnicity and socioeconomic status. Sustained examination at this scale will enable the implementation and evaluation evidence-based policies to reduce housing loss.

## Supplementary Material

Supplementary File

## Data Availability

Anonymized data (downloadable dataset, aggregated at the county level) have been deposited at the Eviction Lab website (https://data-downloads.evictionlab.org/#estimating-eviction-prevalance-across-us/).
