# Supplementary material for: Estimating eviction prevalence across the United States
Source: Proc Natl Acad Sci U S A. 2022 May 16;119(21):e2116169119. doi: 10.1073/pnas.2116169119 (PMC9173767; doi:10.1073/pnas.2116169119)
Supplement: Supplementary File [file pnas.2116169119.sapp.pdf]

Supplementary Information

Estimating Eviction Prevalence across the United States

Contents

1. Data and Measurement
2. Data Validation
3. Bayesian Estimation of Eviction Filings
4. Bayesian Estimation of Households Threatened with Eviction
5. Robustness Analyses
6. Eviction Notice Requirements
7. Eviction Judgments
8. Supplementary Tables
9. Supplementary Figures
10. Supplementary References and Notes

1 Data and Measurement

Public records of eviction cases provide an opportunity to systematically examine the prevalence of eviction filings in the United States (US), but they do not share a standardized format or method of collection. Large-scale aggregation of these records makes these data assessable to researchers, policymakers, and the general public to facilitate investigation of the prevalence, causes, and consequences of eviction lawsuits. We have taken the first step in this direction by collecting, cleaning, and standardizing publicly available eviction records from all 50 states and the District of Columbia (DC), which has resulted in the largest national database of eviction cases to date. Although our goal was to build a comprehensive national database, it is not possible to collect electronic records of all eviction cases filed annually in the US. To overcome this limitation, we developed a novel method of triangulating multiple sources of data on eviction lawsuits to produce comprehensive annual estimates of eviction filings and households threatened with eviction.

1.1 What is an Eviction Case?

Eviction cases are civil lawsuits filed by landlords to remove tenants from rental properties or collect past-due rent or other monetary damages (*1*). The filing represents only the first action in the formal eviction process. Tenants must respond to the filing, either by answering the summons or appearing in court. If the tenant fails to respond, a default judgment in favor of the plaintiff is usually issued by the court. If the tenant responds, the case proceeds and can be resolved in many ways. The case may be dismissed due to insufficient action or evidence by the landlord or because the tenant has already vacated the property. The landlord and tenant may resolve the dispute by mutual agreement, which may or may not stipulate that the tenant vacates the property or repays past-due rent. A judge may decide the case in favor of the landlord or tenant after a court trial. If a tenant is ordered by the court to vacate a property but does not leave voluntarily, the landlord can file a writ of restitution with the sheriff or marshal to forcibly remove the tenant.

Records of eviction cases are typically held in electronic case management systems or paper files in the local court where the case was filed. Some states have standardized electronic case management systems that are used across all (or most) local courts. In these areas, case records often can be straightforwardly compiled into aggregated state-wide databases. In states without standardized systems, local courts may only report the total number of cases filed without case-specific information. Currently, there is no systematic collection of eviction case records or reporting of eviction filings across states by federal agencies.

1.2 Data Collection

We used three strategies to collect eviction case data. First, we made bulk requests for individual electronic records directly to the state courts in all states and DC. We requested all publicly available case records, regardless of when the case was originally filed. We received electronic records directly from 16 states and five counties. We downloaded data for DC and Virginia from available repositories of records scraped directly from courts' online case management system portals. We were sent eviction records that other researchers had previously collected for Washington (state); Philadelphia County, Pennsylvania; and Johnson and Wyandotte Counties in Kansas. In total, we obtained eviction records for 19 states and eight counties (Table S1). We made these records

requests between June 2016 and January 2019. In areas with available data, many of the requests were fulfilled near the beginning of this timeframe, resulting in greater representation of these records in 2016 and earlier years. We refer to these data as court-issued individual records.

We were not able to collect data via bulk records requests from all states for two primary reasons. First, not all states maintain statewide electronic case management systems that compile records across courts. In some states, these systems are just beginning to be implemented (e.g., the Maryland Electronic Courts [MDEC] system) and do not include digitized historical records. Second, some states have policies prohibiting bulk collection of court records by third parties, including researchers. For example, Kansas Supreme Court Rule 196 prohibits bulk records requests in District Courts (*2*).

Second, we also requested annual, aggregated counts of eviction filings at the county level from state and county courts. We began making these requests in October 2017 and continued through April 2020. While lacking in case-specific information, these aggregated filing counts served two purposes. First, we used these data to validate the case counts aggregated from individual records. Second, these data provided information about the filing volume in areas with limited or no individual records data. We requested county-level filings because this is the smallest areal unit in which states consistently tabulate and release these data. We were able to collect at least one year of aggregated filing counts from 2,204 counties across 46 states (Table S2). Throughout the text, we refer to these data as court-issued aggregated data.

Third, we purchased proprietary individual records data from LexisNexis Risk Solutions (LexisNexis). LexisNexis conducts automated and in-person bulk collection of records from local civil and housing courts in most states. Automated collection typically involves bulk transfer of electronic records from the courts directly to LexisNexis. In-person collection, on the other hand, requires the manual entry of case information from on-site records in courthouses, which is much more labor intensive and time consuming. Some courts have restrictions on bulk requests of paper files or store records off-site, which increase the difficulty of in-person data collection. Due to the sheer volume of eviction cases filed annually and lack of available electronic bulk records collection across courts, it is not realistic to expect that the proprietary data will include the universe of eviction filings. Even so, the proprietary data provided an important signal of eviction case activity in areas where we were not able to obtain records directly from the courts, such as states that do not have unified case management systems or prohibit bulk requests of electronic court records.

To be clear then, both the court-issued and proprietary data were built on case records held by local and state courts. We were only able to request electronic individual records or aggregated counts of case filings, while LexisNexis collects records from both electronic and paper case management systems. In county-years in which we were able to obtain both court-issued and proprietary records, there was significant overlap in case representation across these data sources, as expected.

Although we did not place time restrictions on our data requests of the courts or LexisNexis, we restricted the analytic sample for this project to residential eviction cases filed between 2000 and 2018. Many court systems lacked consistent digitization of case records before this period (Table S1), which limited both the representativeness of our data and the ability to validate filing counts across data sources before this period.

1.3 Data Cleaning

Court records are created for administrative rather than research purposes and the format, quantity, and quality of case information varies substantially across sources. We cleaned and standardized all individual court records, regardless of whether we obtained them directly from the courts or LexisNexis. We received records electronically, usually in the form of text-delimited files. The files were structured (i.e., the information was separated into labeled fields as opposed to unformatted text or document images) but did not share a standardized format across sources. Information was recorded at different levels across files. Some files had all case information contained within one observation. Other data files contained separate observations for each party (plaintiff or defendant) on a case. Still others had multiple observations for separate actions (e.g., filings, judgments) associated with a case. In the latter two formats then, some cases were represented by multiple observations. Unique cases were identified by the county name (or numeric county id), court id, and case number.

The available data elements for cases varied across sources as well. Most files included a basic set of information: filing date, landlord-plaintiff name(s), tenant-defendant name(s), defendant or property address, judgments entered on the case, corresponding judgment dates, and monetary amounts defendants were ordered to pay the plaintiff (if applicable). Some data files included additional pieces of information, including whether a writ was issued for the sheriff's office to remove tenants from the property (Virginia), names of judges assigned to the case (Philadelphia County), or itemized monetary judgment amounts (Pennsylvania). Given our goal of creating comparable eviction metrics across states, however, we were only able to fully utilize the data elements that were consistently available in most files for this project.

We can gain important indicators of eviction prevalence from these records. At the most foundational level, we can count how many residential eviction cases were filed within a particular place in a selected time period. Case-level address information allows aggregation of filings in smaller areal units, including census tracts, than are available in annual reports of case filings released by courts. We can then use the combination of tenant names and addresses in these records to move beyond total filings to distinguish how many unique households have experienced an eviction filing over time. This constitutes a separate measure from the overall number of filings as some households receive multiple eviction filings at the same address. The total number of filings represents the burden of eviction on the legal system within a jurisdiction, while the number of households receiving at least one eviction filing is a more direct measure of the burden of eviction on renters. The difference between these numbers can also impart important information on how landlords use the legal system to manage rental properties and variation in landlord behavior across jurisdictions (*3*, *4*).

These records do not allow us to measure how many households were displaced following the case filing or how often households were forcibly removed from rental properties. Courts do not track whether tenants remain at disputed properties throughout or following an eviction case. Default judgments may be issued against tenants who have already vacated the property. Landlords and tenants may resolve the dispute without the tenant leaving the property even after a judgment in favor of the landlord has been returned on the case. Most courts do not track whether cases result in the issuance of a writ of restitution for the disputed property, and even those that do may not reliably update records when the writ is cancelled or unexecuted. While many court records contain some form of judgment information, the quality and type of information varies significantly across

data sources, making it difficult to create comparative metrics. For this reason, we cannot create a nationally comprehensive estimate of eviction judgments or households displaced due to eviction. We discuss these limitations in greater detail in Section 7.

1.3.1 Case Location

We needed to assign each eviction case to a precise geographic location to aggregate filings into standardized areal units (e.g., Census tract). For the data files that included a designated field for the address of the disputed property, we used that as the case address (*5*). For the remaining data files we used the defendant address as the case address under the assumption that most cases were initiated while the tenant resided at the disputed property (*6*). We cleaned and geocoded addresses to obtain latitude and longitude coordinates.

1.3.2 Address Cleaning

We cleaned the case addresses prior to geocoding to increase the likelihood they would be matched to a known street address. We did this in several steps:

1. We capitalized all alphabetic characters and removed excess white space in all fields containing address information.

2. Some data files included all address information in a single field, while others contained separate fields for street address, city name, state, or zip code. In some cases, data files with separate address fields were incorrectly or incompletely split. We ensured that addresses were accurately split into five distinct fields:

   - street address
   - unit designation
   - city name
   - state abbreviation
   - 5-digit zip code

   We used regular expressions to separate city names, state abbreviations, and 5- or 9-digit zip codes. We removed punctuation (with the exception of dashes and apostrophes) and other non-alphabetic characters from city names. We standardized state abbreviations to valid two-letter strings representing US states. We verified that the zip code field contained only numbers and shortened 9-digit zip codes to five digits. We left partial 3- or 4-digit zip codes unchanged.

3. We removed business, individual, or care-of (C/O) names from street addresses by searching for and removing strings of alphabetical characters that appeared before street numbers.

4. We removed post office box information from street addresses to prevent erroneous matches to street names during geocoding by searching for and removing common variations of this information (e.g., "PO BOX", "POB", "PMB").

5. We separated apartment designations from street addresses by searching for common unit signifiers (e.g., "APT", "UNIT", "SUITE", "NO", "#") and transferring these elements into a separate field.

6. We corrected any cases where street address and apartment designations had been inverted in the original data by searching for cases in which the street address field did not begin with numeric characters (i.e., the street number), but the apartment (or second street address) field did.

7. We standardized street type abbreviations (e.g., "AVE" to "AVENUE", "ST" to "STREET", "DR" to "DRIVE") and numeric designations (e.g., "1st" to "First", "2nd" to "Second", "ONE" to "1").

8. We cleaned city names, state abbreviations, and 5-digit zip codes via automated comparison to a standardized listing of city names and state abbreviations associated with US zip codes (*7*). We conducted this standardized comparison listing using two sources: Zip Codes To Go and US Census Zip Code Tabulation Areas. Zip Codes To Go (www.zipcodestogo.com) maintains an up-to-date listing of all zip codes used by the United States Postal Service (USPS), along with the primary city name and state abbreviation associated with each zip code. The US Census provides Zip Code Tabulation Areas (ZCTAs) that roughly align with USPS zip codes (*8*). We combined the listings of city names, state abbreviations, and zip codes from both sources into one master file of valid city name, state abbreviation, and zip code combinations.

   We then compared each city name, state abbreviation, and zip code in the eviction records to the master file. We used the *matchit* program in Stata, which performs fuzzy string comparison, to find the best standardized match for each record using bigram vectoral decomposition distance. Bigram vectoral decomposition breaks each string into two-letter segments and then calculates a similarity score recording the proportion of these segments that align across two strings. The highest possible similarity score was 1, which indicated that a city name, state abbreviation, and zip code from a case address perfectly matched a city, state, and zip code listing in the master file. Most case addresses (95.6%) were matched with a similarity score of 1.

   Some of the matches with similarity scores below 1 were corrected versions of the city name, state, and zip code in the case addresses. We marked matches with similarity scores below 1 as correct in the following situations:

   - Similarity scores greater than 0.70 with a perfect match for the city name and zip code. In these cases the state abbreviation in the eviction record was incorrectly entered, as zip codes do not repeat across states.
   - Similarity scores greater than 0.70 with a perfect match for the state abbreviation and zip code. These matches represented misspellings in city names (*9*).
   - Similarity scores greater than 0.70 with a perfect zip code match. These matches represented minor misspellings in city names and incorrect state abbreviations.
   - Similarity scores greater than 0.75 with a perfect match for city name, state abbreviation, and only one unmatched digit in the first four digits of the zip code. Zip codes are

assigned in a consistent pattern across states such that one unmatched digit (excluding the final digit) is very likely to represent an error.

- Similarity scores greater than 0.75, with a perfect match for city name, state abbreviation, with two consecutive digits of the zip codes inverted. Again, if two digits are simply inverted, this is more likely to represent a data entry error than a distinct, unknown location.

If the matches between the case addresses and master listing were marked as correct, we updated the cleaned address fields in the case address with the standardized city names, state abbreviations, and zip codes in the master file. City names, state abbreviations, and zip codes were updated for 1.7% of case addresses using these criteria. Suggested matches that did not meet any of the above criteria were not used to update the case addresses (2.7% of addresses) (*10*).

These were the general rules used for cleaning addresses, but we checked corrections as we worked and developed more specific rules for addressing particular variations of these problems. More specific examples are discussed elsewhere (*11*).

1.3.3 Geocoding

We geocoded cleaned case addresses to obtain two additional pieces of information:

1. A standardized representation of the address that is consistent across records.
2. The latitude and longitude coordinates that can be used to assign cases to areal units.

The case addresses were geocoded using the 2016 Environmental Systems Research Institute (ESRI) USA Street Address Locator Files. In total, 93.7% of addresses could be geocoded at the point- or street-address level. The point-address level assigns coordinates based on the house or building address. The street-address level assigns coordinates based on a street number falling within a specific range on a particular street (e.g., "123 Main Street" would be assigned to the 100-200 block of Main Street). Both the point- and street-address levels represent locations with a high degree of geographic precision. For addresses geocoded at these levels, we updated the case address fields to reflect the standardized addresses returned during the geocode. For addresses geocoded at less precise levels, we did not update the case addresses. When this was the case, we continued to use the cleaned address as it was before geocoding (6.3% of cases). We retained all returned latitude and longitude coordinates for case addresses but created an indicator for addresses geocoded at the point- or street-level so that we would be able to restrict the sample when aggregating in small areal units that require a high degree of precision for assignment, such as Census tracts.

1.3.4 Address Standardization

Even after cleaning and geocoding, some case addresses still appeared in multiple variations in the data. This prevented us from identifying the same household across cases, which was important for determining the number of unique households threatened with eviction. We standardized multiple versions of the same address appearing for the same defendant by identifying the following patterns:

1. The street address, state, and zip code matched, but one or more entries had a different city name, e.g., "123 MAIN STREET, HENRICO, VA 55555" and "123 MAIN STREET, RICHMOND, VA 55555." These were usually neighboring cities. We updated all entries to share the same city name, with preference given according to the following ordered criteria:
   (a) The version of the address geocoded at the point or street level (the most precise levels of geography).
   (b) The version of the address that could be geocoded at any level of geography.
   (c) The version of the address that was verified as a valid city name, state, and zip code combination (Item 8 in Section 1.3.2).
   (d) The version of the address that appeared most frequently in the records.
2. The street address, state, and city name matched, but one or more entries had a different zip code. As with variations in city names, this commonly occurred near zip code borders and these addresses were updated to share the same zip code. We used the same set of criteria listed above.
3. The apartment designation was appended to the street number, e.g., "123 MAIN STREET, APT 2B, ANYWHERE, US 55555" and "1232B MAIN STREET, ANYWHERE, US 55555". We identified these cases by searching for instances in which both the street and apartment numbers associated with one address appeared within the street number of another address. It was important to identify these cases because addresses with apartment designations appended to the street number often could not be geocoded. We updated concatenated versions with the correct separation of street number and unit designation.
4. Addresses shared the same street number and had street names that had either (1) a Levenshtein string edit distance of one character or less or (2) the containment of one street name within the other (e.g., "MAIN" and "MAINE"). We again updated addresses using the same ordered criteria specified for city names (*12*).
5. For addresses in DC, we extracted street directions (e.g., NE, NW, SE, SW), standardized the format to the two-letter abbreviation, and placed them uniformly at the end of the street address.

For addresses updated during this process, we also updated the standardized representation of the address and geographic coordinates obtained from the geocode.

1.3.5 Multiple Addresses

Some data files without designated property addresses had cases that listed multiple defendant addresses. In some instances, different addresses were associated with different defendants. In others, multiple addresses were listed for the same defendant, which may have resulted from defendants leaving the disputed property while the case was ongoing. Less than 10% of cases across data files had multiple defendant addresses, indicating that most defendant addresses likely represented the property address.

If multiple addresses appeared on a case, we selected the address with the highest likelihood of representing the disputed property using the following criteria:

1. The address associated with the original case filing.
2. The address associated with a forcible detainer judgment or dismissal (if present). A forcible detainer judgment restores possession of the property to the landlord and is likely to reflect the location of the property at stake.
3. The modal case address.
4. The address associated with the earliest case action.
5. The in-state address (if in-state and out-of-state addresses are listed).
6. The address that could be geocoded at the point or street level.
7. If more than one candidate address remained for a case, we randomly selected an address.

1.3.6 Commercial Defendants

As we are studying housing eviction, we excluded cases with commercial defendants. The proprietary data already included an indicator for commercial defendants. To identify commercial defendants in the court-issued data, we developed a list of key words commonly associated with business entities and used regular expressions to identify defendant names that included these keywords (*13*).

1.3.7 Unique Defendant Identifiers

Administrative records may contain multiple representations of the same defendant name due to data entry errors or variations in how the name was listed on multiple case filings. Variations in names for the same person across multiple records impede our ability to identify duplicate records or multiple filings against the same household. We need to be able to identify multiple filings against the same household to estimate the number of unique households threatened with eviction (Section 4). We defined a unique defendant as the same individual residing at the same address.

To find instances in which variations of the same defendant name and address appeared across multiple records, we used the *fastLink* probabilistic record linkage program in R (*14*). The program calculated similarity scores by first name, last name, and street address using Jaro-Winkler string distance (*15*,*16*). We considered a full match to be a similarity score of at least 0.95 and a partial match to be a similarity score of at least 0.92 (for reference, an exact match would have a similarity score of 1). We marked observations as representing the same defendant if all three fields were full matches or the last name was a partial match and the first name and street address was a full match.

We created unique defendant identifiers by treating the *fastLink* matches as undirected edges of a network graph. In this case, each raw name and address observation was a node in the network, with matched combinations representing ties between nodes. Each connected component created by the ties represented a unique defendant. We used the *pooh* package in R (*17*) to calculate the equivalence classes using weak tie transitive closure for the components in the network. Each resulting component was assigned a sequential number, which then served as the unique defendant identifier.

1.3.8 Household Identifiers

Landlords may not list all persons residing in the household as defendants on an eviction case. If multiple cases are filed against the same household over time, there may be variations in defendants listed on each filing. We created unique household identifiers by grouping together cases located at the same case address that shared at least one defendant name in common. If multiple cases were listed at the same address but shared no defendants in common, they were considered cases against separate households.

1.3.9 Duplicate Records

Data entry errors can lead to the same case being entered into the case management system multiple times. Failing to identify and exclude these records can inflate estimates of the number of case filings. We searched for duplicate records by the unique defendant identifier (same defendant name and address), date, case action, and judgment amount (if a monetary judgment was issued on that date). It is important to note that duplicate records do not represent multiple, distinct filings against the same households, which we discuss in Section 1.3.10. These are records that duplicate the entry of a filing that has already been captured in the records (e.g., same filing date, same outcome). We marked duplicated records both within and across case numbers. The former represent duplicated actions on the same case while the latter represent duplicated cases within the court system. Duplicated cases often appeared to be the product of small variations in the case numbers. To avoid over-counting cases, we linked duplicated records (both within and across cases) under one updated case number. We also checked for duplicated records across the same defendant and case action date. Multiple, separate actions could appear on the same date for a case; we ensured that these records were linked under the same case number.

1.3.10 Identifying Case Series

Case series refer to a set of cases filed against the same household. Identifying repeated case filings against tenants at the same residence challenges the assumption that the intention of an eviction filing is to remove a tenant from the property and illuminates other ways landlords use the legal system to manage rental properties (*3*, *4*). It also helps us distinguish the total volume of case filings from the number of households threatened with eviction—two distinct measures of eviction prevalence. We used the household identifiers (Section 1.3.8) to identify case series.

1.3.11 County-level Aggregation

We summed the number of eviction filings and unique households threatened with eviction for all US counties annually for 2000-2018. We defined our geography according to the 2010 Census (N=3,143 counties). Each case was assigned to the county and calendar year in which it was filed (N=59,717 total county-years). All data received directly from the courts included filing dates (or filing year, in the case of court-reported aggregated filing counts). When filing dates were not available in the proprietary data (approximately 32% of cases), we assigned the case to the calendar year of the earliest recorded action. For eviction filings, we simply summed all cases by county and calendar year. To measure unique households threatened with eviction, we again summed cases

by county and calendar year but only counted the first (or only) appearance of a household in that county-year.

1.3.12 Covariates

To better understand how eviction prevalence varies across counties, we also collected a set of demographic and court characteristics for each county-year. The demographic covariates include the number of renting households, total population, population density, household density, percent urban population, percent renting households (of total households), share of population by race/ethnicity (Black/African American, Hispanic/Latino, Native America, Asian, Pacific Islander/Native Hawaiian, other race/ethnicity, two or more race/ethnicities, and non-Hispanic white), median income, median property value, median rent, mean rent burden (defined as the average percent of income spent on housing), share of population living at or below the poverty line, and unemployment rate. The court characteristics include the cost to file an eviction case, availability of a public access terminal in courts, and the number of courts that hear eviction cases in the county.

We estimated the number of renting households using linear interpolation of block group-level data from the 2000 and 2010 Censuses and 2016 ESRI Business Analyst. We obtained renting household data for the 2000 Census in 2010 Census geography from the IPUMS National Historical Geographic Information System (*18*). We extrapolated the linear interpolation for 2017-2018. We then aggregated block groups to create counts of renting households at the county level. We calculated household density by dividing the number of renting households in the county by the total land area reported in the 2010 Census.

We downloaded Census measures of percent county population residing in urban areas from the Missouri Census Data Center (*19*,*20*). These measures were only available for 2000 and 2010. We used linear interpolation to create percentages for the intervening years and extrapolated estimates to 2018.

We used measures of total population, share of population by race/ethnicity, median income, median property value, median rent, mean rent burden, and share of population living at or below poverty line from the 2000 and 2010 Censuses and four waves of the 5-year American Community Survey (ACS): 2005-2009, 2008-2012, 2011-2015, and 2014-2018. We used 5-year rather than 1-year ACS estimates as many counties had fewer than 65,000 residents, thus not meeting the population threshold for inclusion in 1-year ACS data. We assigned yearly data as shown in Table S10. To calculate population density, we divided total population by the total land area reported in the 2010 Census. We obtained the annual unemployment rate from the National Bureau of Labor Statistics. All variables were measured at the county level.

Information on the availability of public access terminals and the number of courts that hear eviction cases in each county was collected from court information provided by the Public Record Research System (*21*). Public access terminals allow the public to view electronic case records in the courthouse (as opposed to having to request paper files from the court clerks) and may increase ease of access to records. The availability of public access terminals was used as a binary measure, with "1" indicating that courts had public access terminals. The data also included an indicator of whether a court heard eviction cases. To measure the number of courts that hear eviction cases, we summed this number within counties.

We calculated several additional data collection measures directly from the proprietary data to estimate how well these data should be expected to compare to the court-issued case counts. First,

we calculated the percentage of records collected through an automated source (rather than in-person collection). We expected automated collection to increase data coverage as it should be less time and resource intensive than in-person collection, although challenges with consistent automated collection in some systems may also result in decreased coverage. We marked cases with records collected via automation through the collector id included in the data file. Second, we calculated the total number of data uploads that occurred in each county-year by counting the unique upload dates appearing on records; a greater number of uploads may represent increased effort to collect records from that area. Third, we might expect data coverage to differ by record collectors; some collectors may systematically collect a higher volume of cases than others. While we did not know the identity of the record collectors, we were able to identify the collector that contributed the majority of records to the data in a county-year and calculate the proportion of records contributed by that collector. Fourth, we calculated the percent of cases that were dismissed or appeared unresolved. Collection of dismissed cases may be a lower priority (or prevented by some states), rendering these cases more likely to be systematically missing. If records of the case filing were collected prior to the case being dismissed, it may appear in our data but without judgment information. Lack of dismissed (or unresolved) cases in the data may indicate an incomplete set of filings for that county-year.

1.3.13 Eviction Case Filing Fees

Landlords (or their agents) are required to pay an administrative fee when filing an eviction case in court. Some states set state-wide fee schedules, while others allow filing fees to be determined by counties (or the type of court). We collected data on the cost to file an eviction case manually. Research interns recorded the total minimum dollar amount required to file an eviction case in each county through either official fee schedules or case information posted on the court website or contact via phone or email with court clerks (*22*). We were able to successfully collect current fee schedules in 2018 for 3,116 of 3,143 counties (99.1%). In 28 states, filing fees were consistent throughout the state (i.e., the same across all counties). Even in states without fixed state-wide fees, the cost to file a case tended to be very similar across counties (Table S6). We confirmed that the within-state variance was significantly less than the between-state variance by fitting a fixed-effects model at the state-level. The fraction of variance attributable to the state-level intercepts was 0.953, with results from an F-test of the null hypothesis that all intercepts were indistinguishable from 0 significant at $p < 0.001$ ($F(50, 3065) = 1023.82$).

1.3.14 Regional and Urban-Rural Classification of Counties

To examine eviction prevalence across different areas, we classified counties by level of urbanization and region. We used the 2013 National Center for Health Statistics (NCHS) urban-rural classification scheme (*23*) to separate counties into six levels of urbanization:

1. Large central metro area: Counties located in Metropolitan Statistical Areas (MSAs) with populations of at least 1 million that either contain the entire population of the largest principal city of the MSA, are entirely contained in the largest principal city of the MSA, or have populations of at least 250,000 residents of any principal city included in the MSA.

2. Large fringe metro/suburban: Counties located in MSAs with populations of at least 1 million that do not satisfy the requirements for large central metro areas.
3. Medium metro: Counties located in MSAs with populations of 250,000 to 999,999.
4. Small metro: Counties located in MSAs with populations less than 250,000.
5. Micropolitan: Counties located in Micropolitan Statistical Areas.
6. Non-core/Rural: Counties not located in Metropolitan or Micropolitan Statistical Areas.

We assigned counties to Census regions (Northeast, Midwest, South, and West) by state (*24*).

2 Data Validation

2.1 Court-Issued Data

Although court-issued data were expected to represent all eviction cases that were processed through the courts' case management systems, staged implementation of new case management systems, changes in record-keeping, and inconsistent reporting by local courts can result in incomplete representation of eviction filings. After cleaning the individual records data and aggregating filings by county-year, we validated our court-issued county-year filing counts to ensure we had complete and consistent data coverage across counties. We did this in two steps. First, we compared filing counts in county-years in which we had both court-issued individual records and aggregated filing counts. We expected these filing counts to be very similar as we received both directly from the courts rather than through secondary data collection. We marked instances in which the filing counts differed by more than 10% and investigated discrepancies. Oregon was the only state with consistent, significant discrepancies. We determined that Oregon's court-issued individual records were not consistently inclusive of cases over time. For this reason, we excluded individual court-issued records from Oregon from the analyses.

Second, we marked yearly fluctuations in the filing counts from the court-issued data within each county. We did this for both the court-issued individual records and aggregated filing counts. We flagged years in which the filing counts increased or decreased by 50% or more (*25*). We also flagged county-years that had court-issued filing counts that were less than 50% of filing counts observed in the proprietary data. We reviewed all instances that were flagged by one or more of these criteria and excluded filing counts that appeared incomplete. We identified the following issues:

Court-issued individual records

1. We excluded most county-years for Indiana due to inconsistent implementation of the state's electronic case management system.

2. South Carolina had 49 county-years from 11 counties excluded due to inconsistent data reporting.

Court-issued aggregated filing counts

1. We excluded county-years in Arkansas that also allowed eviction complaints to be filed as criminal "Failure to Vacate" cases (*1*). The data we received for Arkansas included only civil cases, resulting in an undercount of eviction filings in these county-years.

2. Filing counts for Georgia and Texas were reported at the court level, but not all courts consistently reported filings. We excluded county-years with one or more missing filing counts from courts that heard eviction cases in the previous year.

3. The filing counts we received for New York counties in 2017-2018 (excluding New York City) did not include cases heard in local or district courts, likely undercounting case filings. We excluded these county-years.

We excluded a small number of additional county-years of court-issued data (both individual records and aggregated filing counts) due to inconsistencies that did not appear to be part of a

systematic or long-term pattern. These may represent more isolated data entry, data collection, or case management system errors. Table S3 summarizes availability and exclusion of court-issued data across states.

Our goal was to produce a comprehensive set of eviction filing estimates across all 3,143 US counties from 2000-2018 (N=59,717 county-years). After validating the court-issued data, we had 9,432 county-years (in 935 counties across 20 states) with reliable filing counts from individual records and 29,545 county-years (in 1,930 counties across 44 states) of reliable aggregated filing counts. We used the filing counts from the court-issued individual records in the available county-years (N=9,432). We used filing counts from the court-issued aggregated data in county-years with reliable aggregated data but missing or unreliable court-issued individual records (N=23,815). We gave preference to the individual records when both sources of court-issued data were available as we were able to review these data and remove commercial and duplicated records. Again, we removed only duplicated records that represented re-entries of cases already included in the data, not multiple, distinct filings against the same households. For the 2000-2018 period, we were able to observe filing counts from court-issued data for 33,247 county-years (55.7% of the total 59,717 county-years).

Filing counts from court-issued individual records and aggregated data were very similar in county-years in which we reliably observed both (N=5,730 county-years in 681 counties across 13 states). Filing counts reported in the court-issued aggregates were 1.7% higher, on average, than filing courts generated from the individual records when there were at least 20 case filings reported in the individual records. When including all county-years with reliable court-issued individual records and aggregated data, the mean difference was 2.4%, but this reflected the fact that very small differences of one or two records can result in large percentage differences when the total number of filings is small. There was some variation in this difference across states, making it difficult to make blanket assumptions about whether or how to adjust court-issued aggregated filing counts. In 2,971 of these county-years (51.8%), there was no difference between the individual records and aggregated filing counts. When there was a difference, the aggregated filing counts were more likely to be higher (N=2,244, 39.2% of county-years) than lower (N=515, 9.0% of county-years) than filing counts generated from the individual records, as expected. Due to the variation in the difference and the relatively small overall discrepancies, we decided not to make a blanket adjustment to the court-issued aggregated filing counts. If we would have made a blanket adjustment—1.7% for court-issued aggregates with 20 or more filings and 3.5% for aggregates with fewer than 20 filings (the average difference between individual records and court-issued aggregates when fewer than 20 filings were reported in the individual records)—it would have had very little impact on estimated filing counts. The national number of filings would have been reduced by 32,472, less than 0.9% of total filings, on average, annually. As we cannot assess the full distribution of possible differences between court-issued individual records and aggregated filing counts across counties and states, we decided against adjusting the court-issued aggregates before incorporating them into the national eviction filing estimates or Bayesian model (as we wanted the Bayesian model to preserve county-level variation in case filings as much as possible). The rough calculations presented above suggest that the decision not to adjust court-issued aggregated filing counts should have made very little difference for estimates.

2.2 Court-Issued and Proprietary Data

For county-years without reliable court-issued data (N=26,470), we needed a novel method to estimate the number of eviction filings. On one hand, we had at least one year of validated, court-issued case filings in 2,272 counties in 49 states in the 2000-2018 period; on the other, we were missing court-issued data for at least one year in a similar number of areas (2,673 counties in 49 states). This incentivized the development of a hierarchical model that allowed us to borrow information about filing volume within counties and states across years in which we were missing court-issued filing counts, when possible. We incorporated the proprietary data as a secondary measure of court-issued filing counts. We did not use proprietary filing counts as a direct measure of filing volume due to concerns about the inability to capture all case filings, as discussed in Section 1.2.

We observed at least one filing in the proprietary data in 75.3% of county-years (N=44,953 of 59,717 county-years). Of these 44,953 county-years, 25,715 (57.2%) had validated court-issued filing counts. The coverage of filings in the proprietary data relative to the court-issued filing counts varied considerably across these county-years (Table S11). In very few county-years were the court-issued and proprietary filing counts an exact match (4.3% of county-years with both validated court-issued data and at least one filing in the proprietary data). In 38.7% of these county-years, the proprietary data were a fairly reliable approximate of court-issued filing counts, showing a discrepancy of fewer than 10 cases or less than 5% of court-issued filing volume. In over half the county-years (52.5%), however, the proprietary data undercounted the court-issued filings by more than 10 cases or 5% of total filing volume. In only 4.6% of county-years did the proprietary data overcount filings by more than 10 cases or 5% of total volume relative to the court-issued data.

On average, the proprietary data undercounted the court-issued filings by 29.8%. In all states, the average difference revealed the proprietary data to be an undercount of the court-issued filings, except for Louisiana. In Louisiana, the proprietary data overcounted court-issued filings in one county but, because we had very few county-years in Louisiana with both court-issued and proprietary data, the overall mean difference was positive. The undercounts of filings in the proprietary data relative to court-issued data were likely the result of the inability to collect the universe of cases due to limitations discussed in Section 1.2. Small discrepancies in filing counts (both over- and undercounts) between the court-issued and proprietary data could also be due to differences in the dating of some cases discussed in Section 1.3.11. For county-years in which proprietary data were unavailable, it is not possible to determine whether it was because there were no eviction filings in that county-year or cases were filed but not collected. Due to the variation in the differences in court-issued and proprietary filing counts across counties and states, we incorporated these counts as a secondary measurement of filing volume in the Bayesian model rather than applying a standard adjustment to the proprietary data across all areas. We discuss how these data were entered into the model in more detail in the next section.

Finally, we were unable to obtain court-issued or proprietary filing counts in 7,232 county-years (in 1,331 counties across 41 states). These county-years needed to be estimated directly from the Bayesian model. Fig. S14 shows the distribution of data sources underlying county-level estimates across years. Although the same number of counties are represented in national estimates of eviction filings and households threatened with eviction each year (N=3,143), the data sources underlying county-level estimates shift across years.

3 Bayesian Estimation of Eviction Filings

3.1 Model Introduction

We considered the proprietary data to be a secondary measure of court-issued eviction filing volume. That is, we considered the validated court-issued data to represent complete annual case filing volume and the proprietary data to be an imperfect measurement of it. As shown in Table S11, the proprietary data typically undercounted filing volume relative to court-issued data, although the variability of the measurement error differed considerably across states. In some states there was very good agreement between proprietary and court-issued filing counts, whereas in others the proprietary filing counts were consistently lower than court-issued filing counts or fluctuated more.

We developed a joint Bayesian model for the court-issued and proprietary filing counts in response to these challenges. The court-issued filing counts were modeled as a function of demographic and court characteristics with an expected association with eviction case prevalence. The proprietary filing counts were modeled as a function of the court-issued filing counts and data collection conditions.

This joint model was then used to generate 25,000 imputed datasets for county-years in which we were missing court-issued filing counts. The multiple imputation of these missing data was generated from the posterior predictive distribution of the joint model. The posterior predictive distribution is the distribution of values for the unobserved court-issued counts given the observed court-issued and proprietary counts, accounting for both the true variability of filing counts and our uncertainty regarding the parameters in the Bayesian model. To perform inference tasks, such as estimating the filing counts for a larger geographical area, the inference task was performed on each imputed dataset and the results from these analyses were combined to provide point estimates as well as measures of uncertainty.

3.2 Data and Notation

Let Y_i be a random variable with realization y_i indicating the court-issued filing count for year $t_i \in \{2000, ..., 2018\}$ and county $c_i \in \{1, ..., n_c\}$, where n_c is the total number of counties and i indexes all county-year combinations. Further, let Z_i be a random variable with realization z_i indicating the county-year filing count from the proprietary data, s_i be the county's state, and $r_i \in \{1, ..., 4\}$ its Census region (Northeast, Midwest, South, West).

In some counties all landlord-tenant cases were included in the reported filing count, whereas in others, only eviction-specific case types were reported. Let $l_i \in \{0, 1\}$ be "1" if all landlord-tenant cases were reported and "0" otherwise. As most landlord-tenant disputes are eviction cases, the discrepancies should be small, but as some court-issued data and all the proprietary data contain landlord-tenant cases, we included this term to adjust for these differences and ensure an apples-to-apples comparison in the joint model.

We included several measures of demographic and court characteristics with an expected association with eviction filing volume in the model. The following county-level covariates were considered:

1. Number of renting households

2. Total population

3. Population density
4. Household density
5. Percent urban population
6. Black/African American population
7. Hispanic/Latino population
8. Native American population
9. Asian population
10. Pacific Islander/Native Hawaiian population
11. Other race/ethnicity population
12. Multiple race/ethnicity population
13. Non-Hispanic white population
14. Median income
15. Median property value
16. Median rent
17. Rent burden (mean)
18. Percent families living in poverty
19. Unemployment rate
20. Percent change in unemployment rate from previous year
21. Eviction case filing fee
22. Number of courts that hear eviction cases
23. Availability of public access terminal in courthouse

For each covariate, we investigated potential model inclusion by calculating the residuals of the currently-fit model at the posterior mode and then investigating the relationship between those residuals and the variable. If a relationship existed, we transformed the variable to render the relationship approximately linear and standardized the variable. The transformations were chosen to make the relationship between the covariate and $\log(y_i + 10)$ approximately linear, and a second transformation was added for three variables in which additional non-linearity in the relationship remained after the initial transformation. Table S12 shows all covariate transformations. Once a variable was included in the model, we investigated the posterior distribution of the parameter to ensure that it was substantively different from zero, and that its inclusion improved model fit. The resulting matrix was denoted by X. We similarly investigated interactions between

covariates and potential variability of the covariate relationships with filing counts by state. We included random slope parameters for household density, median rent, and unemployment rate. We denote the transformed scaled covariates as x_i^{hd} for household density, x_i^{mr} for median rent, and x_i^{u} for unemployment. We investigated using splines and tensors to model the non-linearity in covariate relationships; however, this added computational expense and did not improve model fit. Additionally, these methods would have greatly complicated utilization of random slopes to model any interactions between covariates and state.

As noted in Section 1.3.12, many of the covariates, including total population, share of population by race/ethnicity, median income, median property value, median rent, mean rent burden, and share of population living at or below poverty line, were calculated from decennial census data in some years and 5-year ACS data others (Table S10). Due to sampling differences, ACS data are measured with greater error than the decennial census (*26*). The presence of measurement error in the ACS could potentially lead to understatement of the true covariate effects by the model and lead to less precise filing estimates relative to observing the true values of the covariates. We investigated including ACS margin of error estimates directly into the model, however, we determined that it added too much computational complexity to be practical.

There were several measures from the proprietary data relevant to estimating how well z filing counts should correspond to y (court-issued) filing counts. First, the share of records collected from automated sources was dichotomized into $\leq 50\%$ and $> 50\%$ ($\text{auto}_i \in \{0, 1\}$). Second, the total number of data uploads that occurred in that county-year was dichotomized into ≤ 10 and > 10 ($\text{nups}_i \in \{0, 1\}$). Third, the collector tasked with the majority of record collection in that county-year (col_i). Fourth, an indicator for whether the county-year did not include any dismissed cases ($\text{cds}_i \in \{0, 1\}$). Finally, an indicator for whether the county-year did not include any unresolved cases ($\text{bns}_i \in \{0, 1\}$). Table S13 presents descriptive statistics for all variables included in the model.

Only some of the y_i and z_i values were observed. The indices where y was observed are o^y and the indices where z was observed are o^z. We denote the indices where they were not observed as the compliment ($(o^y)^C$ and $(o^z)^C$).

3.3 Probability Model for Y

Filing counts were transformed using a log plus ten transform and modeled using a hierarchical normal model. This transformation and distribution were chosen for several reasons. First, the resulting random variable was approximately normal. Second, the residuals of the fit model were approximately normal. Third, the residual variance was stable across predicted count levels. Fourth, the variance in the proprietary filing counts depended greatly on a number of covariates, which would have been challenging to model using some other distribution classes. Fifth, using a normal model admits closed form representations for the marginalized distribution of the proprietary filing counts when the court-issued counts are missing (see Section 3.4) and for generating posterior predictive values from the posterior via closed form representation (see Section 3.6). Finally, the model fitting process required extensive computing time and the utilization of many of the other potential formulations greatly increased the computational burden of the Hamiltonian Markov Chain Monte Carlo algorithm.

We also investigated using a hierarchical Poisson model for the raw filing counts but determined that the variability of the outcome (after adjusting for covariates) did not follow the expected

relationship under the Poisson assumption. Additionally, a different model class would have been required to handle the variance model for Z. Along a similar line, we investigated utilizing a binomial model with the number of renting households as the binomial sample size, which was rejected for the same reasons as the Poisson model.

The model utilized a hierarchical specification with counties nested within states nested within regions. We added additional terms for the effect of the covariates, year, and landlord-tenant indicator and state-varying parameters for the effect of household density, median rent, and unemployment rate. We modeled state and year effects as normally distributed. County effects and covariate-state random slopes exhibited heavier than expected posterior tails when modeled as normal, so these utilized T-distributions. We set the degrees of freedom for these at 1, except for the county-level effects, as there was enough information in the data to include degrees of freedom as a parameter with a half-normal prior. The scale parameters for all random effects were given half-Cauchy priors following the recommendation of Gelman (2006) (*27*). The covariate effect parameters were given normal priors with large spreads. All priors were chosen to be weakly- or non-informative. We assessed this by expanding the scale of the priors and checking that little appreciable change was observed in the posterior.

The probability model for Y was specified as

$$\begin{aligned}
\log(Y_i + 10) &\sim \text{normal}(\lambda_i^y, \sigma_i^y) \\
\lambda_i^y &= \beta_{c_i}^c + \beta_{s_i}^s + \beta_{r_i}^r + \beta^l l_i + \beta^X \cdot X_{i,*} + \beta_{s_i}^{hd} x_i^{hd} + \beta_{s_i}^{mr} x_i^{mr} + \beta_{s_i}^u x_i^u + \beta_{t_i}^t \\
\beta^c &\sim \text{scaled_t}(\text{df} = \nu, \text{mean} = 0, \text{scale} = \tau^{\beta^c}) \\
\beta^s &\sim \text{normal}(0, \tau^{\beta^s}) \\
\beta^r, \beta^l, \beta^X &\sim \text{normal}(0, 10) \\
\beta^t &\sim \text{normal}(0, \tau^{\beta^t}) \\
\beta^{mr} &\sim \text{scaled_t}(\text{df} = 1, \text{mean} = 0, \text{scale} = \tau^{\beta^{mr}}) \\
\beta^{hd} &\sim \text{scaled_t}(\text{df} = 1, \text{mean} = 0, \text{scale} = \tau^{\beta^{hd}}) \\
\beta^u &\sim \text{scaled_t}(\text{df} = 1, \text{mean} = 0, \text{scale} = \tau^{\beta^u}) \\
\tau^{\beta^u}, \tau^{\beta^{hd}}, \tau^{\beta^{mr}}, & \\
\tau^{\beta^s}, \tau^{\beta^c}, \tau^{\beta^t}, \sigma^y &\sim \text{half_cauchy}(0, 5) \\
\nu &\sim \text{half_normal}(1, 5).
\end{aligned}$$

and is displayed graphically in Fig. S15.

3.4 Probability Model for Z

The differences between court-issued and proprietary filing counts varied significantly between states. This was true both in terms of the bias of $\log(z + 10)$ compared to $\log(y + 10)$ and its spread. Thus it was important to be able to model both the mean (λ_i^z) and standard deviation (σ_i^z) of the measurement difference with state-varying parameters.

Fig. S16 shows the graphical representation of the measurement error model. This model consists of a bias component, which represents the deviation of the proprietary filing counts from

the court-issued counts, which can vary by state. A variability component for measurement error is also included, which can also vary by state. The model is expressed mathematically as

$$\log(Z_i + 10) \mid Y_i = y_i \sim \text{normal}(\lambda_i^z + \log(y_i + 10), \sigma_i^z),$$

When the court-issued filing count is missing it can be marginalized out via integration resulting in

$$\log(Z_i + 10) \sim \text{normal}(\lambda_i^z + \lambda_i^y, \sqrt{\sigma_i^z \sigma_i^z + \sigma_i^y \sigma_i^y}).$$

Fig. S17 shows the details of the bias portion of the model. Hierarchical parameters for the effect of state, the effect of each covariate by state, and the effect of the majority record collector on bias are all modeled as normal. The hyper-parameter priors were chosen to be normally distributed for central tendency measures and half-Cauchy distributed for the scale parameters. The intercept and landlord-tenant parameters were given flat improper priors, though these were so well determined by the data that any weakly-informative prior has little effect on the posterior. All priors were inspected and determined to be weakly- or non-informative.

The bias of the proprietary filing counts is then expressed mathematically as

$$\begin{aligned}
\lambda_i^z &= \alpha^0 + \alpha^l l_i + \alpha^{\text{col}}_{\text{col}_i} + \alpha^s_{s_i} + \alpha^{\text{nups}}_{s_i}\text{nups}_i + \alpha^{\text{auto}}_{s_i}\text{auto}_i + \alpha^{\text{bns}}_{s_i}\text{bns}_i + \alpha^{\text{cds}}_{s_i}\text{cds}_i + \alpha^{\text{bns,cds}}_{s_i}\text{bns}_i\text{cds}_i \\
\alpha^0, \alpha^l &\sim \text{uniform}(-\infty, \infty) \\
\alpha^{\text{auto}} &\sim \text{normal}(\mu^{auto}, \kappa^{auto}) \\
\alpha^{\text{nups}} &\sim \text{normal}(\mu^{nups}, \kappa^{nups}) \\
\alpha^{\text{bns}} &\sim \text{normal}(\mu^{bns}, \kappa^{bns}) \\
\alpha^{\text{cds}} &\sim \text{normal}(\mu^{cds}, \kappa^{cds}) \\
\alpha^{\text{bns,cds}} &\sim \text{normal}(\mu^{bns,cds}, \kappa^{bns,cds}) \\
\alpha^{\text{col}} &\sim \text{normal}(0, \kappa^{col}) \\
\alpha^{s} &\sim \text{normal}(0, \kappa^{s}) \\
\mu^{auto}, \mu^{nups}, \mu^{bns}, & \\
\mu^{cds}, \mu^{bns,cds} &\sim \text{normal}(0, 10) \\
\kappa^{auto}, \kappa^{nups}, \kappa^{bns}, & \\
\kappa^{cds}, \kappa^{bns,cds}, \kappa^{s}, \kappa^{col} &\sim \text{half_cauchy}(0, 5).
\end{aligned}$$

Fig. S18 provides a graphical representation of the variance portion of the measurement error model. We modeled the measurement error variance using a log-normal distribution. There is, to our knowledge, no standard guidance suggesting a particular distributional form for a hierarchically-specified scale parameter. Log-normal was chosen for two reasons. First, it is restricted to the positive domain and can take any mean and variance value. Second, the hierarchy for log variance can be expressed as a familiar hierarchical normal model. One disadvantage of this distribution is that the density goes to 0 as the variance goes to 0, thus if any of the measurement error variance was at or near 0, the model might have difficultly converging. We inspected the observed measurement errors broken down by all covariates and states and determined that there was appreciable variability at all disaggregation levels.

Covariate effects were modeled as normal with weakly-informative normal priors for the central tendency parameters and half-Cauchy priors for the spread parameters. The intercept was chosen to have a weakly-informative normal prior.

Mathematically, we express the variance portion of the model as

$$\begin{aligned}
\sigma_i^z &= \sqrt{\exp\left(\gamma^0 + \gamma_{s_i}^{auto}\text{auto}_i + \gamma_{s_i}^{bns}\text{bns}_i + \gamma_{s_i}^{cds}\text{cds}_i\gamma_{s_i}^{bns,cds}\text{bns}_i\text{cds}_i\right)} \\
\gamma^{auto} &\sim \text{normal}(\theta^{auto}, \eta^{auto}) \\
\gamma^{bns} &\sim \text{normal}(\theta^{bns}, \eta^{bns}) \\
\gamma^{cds} &\sim \text{normal}(\theta^{cds}, \eta^{cds}) \\
\gamma^{bns,cds} &\sim \text{normal}(\theta^{bns,cds}, \eta^{bns,cds}) \\
\gamma^0, \theta^{auto}, \theta^{bns} & \\
\theta^{cds}, \theta^{bns,cds} &\sim \text{normal}(0, 10) \\
\eta^{auto}, \eta^{bns}, \eta^{cds}, \eta^{bns,cds} &\sim \text{half_cauchy}(0, 5).
\end{aligned}$$

3.5 Inference

The missingness process for Y and Z were assumed to be missing at random and thus could be factored out of the likelihood for Bayesian inference. The likelihood for observation can be expressed in terms of the probability models outlined in the previous sections:

$$L_i = \begin{cases} p(z_i|y_i)p(y_i) & i \in o^y \cap o^z \\ p(y_i) & i \in o^y \cap (o^z)^C \\ p(z_i) & i \in (o^y)^C \cap o^z. \end{cases}$$

The posterior distribution for this likelihood was constructed using the Stan probabilistic programming language (*28*).

3.6 Imputing Missing Data Using the Posterior Predictive Distribution

Given samples of the model parameters from the posterior distribution, we drew imputed samples from the posterior predictive distribution for y_i in cases where it was missing. If we did not observe z_i, then the kth sample ($(Y_i)^{(k)}$) from the posterior predictive distribution was:

$$\log((Y_i)^{(k)} + 10) \mid (\lambda_i^y)^{(k)}, (\sigma_i^y)^{(k)} \sim \text{normal}((\lambda_i^y)^{(k)}, (\sigma_i^y)^{(k)}),$$

where $(\lambda_i^y)^{(k)}$ and $(\sigma_i^y)^{(k)}$ are the kth parameter samples from the posterior distribution.

If we did observe z_i but not y_i, then the kth sample ($(Y_i)^{(k)}$) from the posterior predictive distribution was:

$$\log((Y_i)^{(k)} + 10) \mid z_i, (\lambda_i^y)^{(k)}, (\sigma_i^y)^{(k)}, (\lambda_i^z)^{(k)}, (\sigma_i^z)^{(k)} \sim \text{normal}(\upsilon, \rho),$$

where

$$\upsilon = (\lambda_i^y)^{(k)} \frac{(\sigma_i^z)^{(k)}(\sigma_i^z)^{(k)}}{(\sigma_i^y)^{(k)}(\sigma_i^y)^{(k)} + (\sigma_i^z)^{(k)}(\sigma_i^z)^{(k)}} + \Big(\log(z_i + 10) - \lambda_i^z\Big) \frac{(\sigma_i^y)^{(k)}(\sigma_i^y)^{(k)}}{(\sigma_i^y)^{(k)}(\sigma_i^y)^{(k)} + (\sigma_i^z)^{(k)}(\sigma_i^z)^{(k)}}$$

$$\rho = \sqrt{\frac{(\sigma_i^z)^{(k)}(\sigma_i^y)^{(k)}}{(\sigma_i^y)^{(k)}(\sigma_i^y)^{(k)} + (\sigma_i^z)^{(k)}(\sigma_i^z)^{(k)}}}.$$

After the imputation was complete, we adjusted down filing counts in counties where all landlord-tenant cases (rather than only eviction-specific case types) were reported by subtracting off $(\beta^l)^{(k)}$ from the $\log(y_i + 10)$ filing counts. Parameters estimated from these models are reported in Section 11. We do not report county-level hierarchical parameters from the primary model or majority collector identifiers from the secondary (proprietary filings) model due to space considerations, but the full set of parameter estimates can be downloaded at [url].

4 Bayesian Estimation of Households Threatened with Eviction

Some tenants are taken to court repeatedly by their landlord. Therefore, each filing does not represent a unique household threatened with eviction. Setting aside repeated filings against the same households is important for accurately assessing the number of households threatened with eviction each year. The frequency of these repeated filings varies across counties and states. For this reason, we specified a secondary Bayesian model to estimate the number of unique households receiving an eviction filing, which allowed us to preserve this geographic variation.

For county-years in which we have reliable court-issued individual records, we could directly observe the number of households threatened with eviction. We did this by creating household identifiers (Section 1.3.8) and aggregating unique instances of these identifiers annually (Section 1.3.11). We were able to measure the number of households threatened with eviction from court-issued individual records for 4,711 county-years.

There were several states in which we had court-issued individual records but could not measure households threatened with eviction directly from the data. Connecticut lacked tenant names for cases heard in housing courts, which account for approximately 50% of records. North Carolina was also missing tenant addresses for approximately 50% of cases. Data from DC and South Dakota did not include tenant addresses. Johnson and Wyandotte Counties in Kansas did not include tenant names or addresses. Virginia data included only the city, state, and zip code of a tenant's address. South Carolina included names and addresses, but because the original dataset was at the case level (i.e., one observation per case) the tenant name and address variables were often concatenated and difficult to parse. Without individual tenant names and addresses, we were unable to consistently group filings by household. We were not able to use the court-issued aggregated filing counts to observe or estimate the number of households threatened with eviction as there was no information on who appeared on the case filings or how often.

For states without court-issued individual records with name and address information, we estimated the number of households threatened with eviction from the Bayesian model. We calculated the number of unique households threatened with eviction from the proprietary data in the same manner as we did for the court-issued individual records. We only used proprietary data in county-years with filing coverage of at least 70% of that reported in the court-issued data to ensure that we had adequate representation of the proportion of repeated filings against the same household in the total case volume (*29*). To assess the agreement between the two data sources, we compared the proportion of filings that represented a unique household threatened with eviction using the court-issued and proprietary individual records. We calculated the correlation between these two proportion for the 390 country-years with validated data from both sources and at least 400 filings. The correlation was 0.87, indicating strong agreement.

We then leveraged hierarchical Bayesian models to estimate the number of unique households threatened with eviction for all county-years (threat_i). The number of households threatened with eviction given the number of eviction filings was modeled as binomial, with hierarchical normal effects for state (s), county within state (c), and year within county within state (cy). Demographic predictors were also added for the median rent (medrent_i), population size (pop_i), percentage of families living in poverty (perpov_i), percent unemployment rate (unemp_i), and median income (medinc_i). Additional predictors for the logged number of filings (y_i), year (t_i), and whether the filings represented all landlord tenant cases (l_i) were also included.

Flat priors were used for all population-level predictors and weakly-informative half student-t

distribution priors were used for the hierarchical parameters. The full model specification was

$$
\begin{aligned}
\text{threat}_i &\sim \text{binomial}(p_i, y_i)\\
\text{logit}(p_i) &= \kappa^{\text{intercept}} + \kappa^{y} \log(y_i + 10) + \kappa^{\text{medrent}} \log(\text{medrent}_i + 10) + \kappa^{\text{pop}} \log(\text{pop}_i + 10)+\\
&\quad \kappa^{\text{perpov}} \log(\text{perpov}_i + 10) + \kappa^{\text{unemp}} \log(\text{unemp}_i + 1) + \kappa^{\text{medinc}} \log(\text{medinc}_i + 10)+\\
&\quad \kappa^{\text{l}} l_i + \kappa^{t}_{t_i} + \kappa^{s}_{s_i} + \kappa^{c}_{c_i} + \kappa^{\text{cy}}_{\text{cy}_i}\\
\kappa^{y}, \kappa^{\text{medrent}}, \kappa^{\text{pop}}, \kappa^{\text{perpov}},&\\
\kappa^{\text{unemp}}, \kappa^{\text{medinc}}, \kappa^{\text{l}}, \kappa^{t}, \kappa^{\text{intercept}} &\sim \text{uniform}(-\infty, \infty)\\
\kappa^{s} &\sim \text{normal}(0, \nu^{s})\\
\kappa^{c} &\sim \text{normal}(0, \nu^{c})\\
\kappa^{\text{cy}} &\sim \text{normal}(0, \nu^{\text{cy}})\\
\nu^{s}, \nu^{c}, \nu^{\text{cy}} &\sim \text{half\_scaled\_t}(\text{df} = 3, \text{mean} = 0, \text{scale} = 10).
\end{aligned}
$$

We considered modeling the number of households threatened with eviction jointly with the number of filings by incorporating the binomial model into the larger eviction filing model. This was rejected for two reasons. First, we could only observe the number of households threatened with eviction directly in counties with court-issued filing counts generated from individual records. Second, it added significant computational complexity that led to very long run times.

To generate an imputation sample from the posterior predictive distribution for a county-year, we first imputed a value for the number of case filings from the posterior predictive distribution of the eviction filings model (y_i) and then we sampled a value from the posterior predictive distribution of the household threatened with eviction model using this imputed filings value.

5 Robustness Analyses

We performed several robustness analyses on the estimates of eviction filings and households threatened with eviction to investigate how sensitive our results were to alternative specifications of data sources and measurement. First, we assessed the correspondence between the court-issued data and estimates produced by the Bayesian models. Second, we investigated how incorporating the proprietary data as a secondary measure of filing volume affected national and state-level filing estimates. Third, we evaluated how Maryland's uniquely high filing counts affect national filing estimates.

5.1 Correspondence between Court-Issued Data and Bayesian Estimates

In a perfect world (of eviction data transparency), all states would have complete, electronic repositories of eviction case records. From these records, it would be possible to directly calculate the number of eviction filings and households threatened with eviction annually. Throughout the main text and in our discussion of data collection (Section 1.2), we have discussed the practical, legal, and technological barriers that make this impossible.

Collecting available court-issued filings data (either in individual record or aggregated filing count form) is an important first step in generating estimates of filings and households threatened with eviction; however, relying solely on these data is problematic for two reasons. First, as we have demonstrated in this paper, rates of filings and households threatened with eviction differ across states. We cannot assume that the filing rate observed in one or two states in any given year can be directly transferred to other states for which we were unable to obtain court-issued data. Second, the composition of states and counties represented in court-issued data changes over time. This occurs for many reasons. Courts implement electronic case management systems at different times; some courts digitize historical records and incorporate them into the system, others do not. Changes in record management systems may create inconsistencies in filing counts or delays in producing updated filing counts. Court case management systems are also vulnerable to external threats. For example, we were unable to obtain court-issued aggregated filing counts for the post-2016 period from Georgia due to a malware attack that affected the court system there. Making comparisons across years when there are fluctuations in the states or counties with available court-issued data is problematic because changes in filing counts may be due to sample composition rather than actual changes in the prevalence of filings nationally. For this reason it is important to have a balanced panel of counties included in estimates across years; however, the sources of available data on which to base these estimates may shift over time (Fig. S14).

Due to the fluctuations in availability of court-issued data over time, the composition of the national filing estimates—the proportion of county-years with court-issued filing counts relative to county-years with filing counts generated from the Bayesian posterior distribution—also fluctuated across years (Fig. S19). Relying solely on court-issued data would make it appear as though there have been drastic reductions in eviction filings in recent years, though this would be primarily a reflection of court-issued data availability (Fig. S14) rather than precipitous declines in eviction filings reported in court-issued individual records or aggregated filing counts. Even with the inclusion of Bayesian posterior estimates in county-years in which we were unable to collect court-issued data, there has still been a decline in eviction filings in recent years (Fig. 1), albeit much smaller than that produced by omitting county-years missing in court-issued data (light blue

bars in Fig. S19).

Court-issued eviction filing rates were more similar within than across states over time (Fig. S20), which affirms the across-state differences identified using the national estimates in the main text. Fig. S20 also shows the variation in availability of court-issued data across states over time. Fluctuations in filing rates estimated using only court-issued data reflect two possible sources of variation over time: (1) true changes in the prevalence of eviction filings and (2) changes in the states that had available court-issued data. For example, Maryland has a substantially higher filing rate per renting households than all other states, therefore we would expect a higher average filing rate in the court-issued data in years in which we had these data available for Maryland as compared to years when we did not, even if the true prevalence of eviction filings in Maryland (or other states) did not change. This makes direct comparisons of filing rates using only the court-issued data to the full set of national estimates, which is a balanced panel of all counties in the United States across years, over time very difficult (and possibly misleading).

To investigate the validity of the longitudinal trend in national filing rates reported in the main text then, we examined changes in average filing rates across three time periods in court-issued data, where robust state-level coverage of court-issued filing counts was available. The national estimates presented in the main text showed an increasing filing rate from 2000 to 2008 before declining in recent years. To increase the number of comparison periods in the court-issued data, we split the national trend into three periods: 2000-2003 (period 1), 2006-2010 (period 2) and 2015-2018 (period 3). We calculated the national average filing rate within each of these three periods: 9.1%, 9.6%, 8.1%, respectively. The percentage change in the filing rate between these periods was an increase of 5.6% between periods 1 and 2 and a decrease of 15.1% between periods 2 and 3.

We then created comparable metrics across states with court-issued data across these three periods (Table S14). We only calculated state metrics if we had validated court-issued data for at least 50% of counties in a given year to increase the likelihood that the changes we calculated reflected true state-level trends in filing rates. We calculated the percentage changes in court-issued state-level filing rates analogous to those calculated for the national filing rate. Looking across states, many displayed similar longitudinal trends to those observed in the estimated national filing rate. On average, court-issued state-level filing rates increased by 3.3% between periods 1 and 2; however, after weighting by renting households, the average increase was 5.1%, very similar to the change observed in the national filing rate between the same periods. Likewise, court-issued state-level filing rates decreased by an average of 10.1% between periods 2 and 3, 18.7% when weighted by renting households, again comparable to the change observed in the national filing rate between the same periods. This suggests that the longitudinal trends present in the national filing estimates are reflective of the changes observed in court-issued data.

We also compared the filing estimates produced by the Bayesian model to those reported in the court-issued data for the county-years in which we had validated court-issued data (N=33,247). As shown in Table S15, 60.5% of the county-year Bayesian estimates differed by less than 10 filings or 5% of court-issued filings, demonstrating fairly good agreement between court-issued data and estimates. The remaining county-years were evenly split between overestimates (20.4%) and underestimates (19.2%) by the Bayesian model. The average percentage difference between court-issued filing counts and those predicted from the Bayesian posterior distribution was 10.0%; however, this difference was inflated minor discrepancies in counties with very low filing counts. When restricting to county-years in which the court-issued data reported at least 10 filings, the mean percentage difference between court-issued filing counts and those predicted in the Bayesian

posterior distribution was only 1.8%.

To evaluate the fit of the Bayesian model relative to the court-issued filing counts, we re-fit the Bayesian model with a holdout set of 5% (N=1,668) randomly selected county-years. Fig. S21 shows the court-issued filing counts plotted against the posterior mean predicted values from this model. The figure shows good agreement and no appreciable bias in the estimates. The holdout set relative bias in the transformed outcome ($\log(y_i + 10)$, where y_i = court-issued filing counts) was less than 1%. Additionally, the variance explained in the model for the court-issued filing counts was high (98.0%). Explained variance was slightly higher (99.0%) for the transformed court-issued filing counts ($\log(y_i + 10)$). As might be expected, the number of renting households in a county accounted for a significant portion of the explained variance in number of eviction filings. Adjusting for renting households ($\log(y_i + 10) - \log(rh_i + 10)$), the variance in filing counts explained by the model was 95.0%.

The full set of county-level eviction filing rate estimates in 2018 generated from the Bayesian posterior mean predicted values are shown in the map in Fig. S22. The distribution of predicted filing rates across the US is almost indistinguishable from that presented in the main text, which combined both the court-issued and Bayesian estimated filing counts, for 2018 (Fig. 3A). The full set of posterior mean predictions for all years, including credible interval and coefficient estimates, can be downloaded alongside the court-issued counts of filings and households threatened with eviction at [url].

Finally, Table S16 presents state-level average filing rates across data sources underlying the county-level estimates. States were assigned to data sources based on the predominant underlying data source for county estimates. In some states, county-level filing estimates were drawn from different data sources based on availability of court-issued individual records or aggregated filing counts. In five states, no single data source covered 90% of counties (final column in Table S16). We presented average filing rates from 2015 in Table S16 as this was the year with the best representation of court-issued data (Fig. S14). The assignment of states to data sources varied across years with changes in availability of court-issued data. There was significant variation in the magnitude of average filing rates across states in all data sources. Although it appears that the mean filing rate among states drawing filing counts from court-issued aggregated data was significantly higher than that in other data sources, this was due to the inclusion of Maryland, which has an abnormally high filing rate. Removing Maryland from the court-issued aggregated filing counts reduced the overall mean to 7.4%, which was almost identical to that observed for state-level filing rates generated from court-issued individual records (7.3%). Although the mean filing rates for states with estimates generated from the Bayesian model or with no majority data source are lower than those with court-issued data in 2015, many of these states have very small renter populations. The mean filing rates across data sources fluctuated significantly across years as state-level filing rates are highly correlated over time (Fig. S12) but the underlying sources of data often fluctuated (Fig. S14).

We also plotted the annual percent eviction filings representing unique households for counties in which we observed data on households threatened with an eviction filing (see Section 4 for a discussion of the observed data for households threatened with eviction) along with rates estimated from the Bayesian model for these same county-years (N=12,870). The rates show very good correspondence across all years (Fig. S23). These proportions are used to calculate the number of households threatened with an eviction filing each year. Again, as with eviction filings, these percentage non-repeated filings against the same household should not be directly compared to the full set of national estimates for households threatened with eviction (Fig. 1B) as the counties and

states with available observed data fluctuated across years. It may appear as though the percentage of unique households represented in eviction filings decreased from 2009-2013 before returning to previous levels in 2018; however, this decrease is due in large part to the availability of observed data in South Carolina and Virginia for 2010-2016. South Carolina and Virginia, which both have higher than average rates of repeated filings against the same households (Fig. S11), only had data on households threatened with an eviction filing available in these years, which had a noticeable effect on the average percentage of filings representing unique households.

5.2 Eviction Filing Estimates without Proprietary Data

We also assessed how the inclusion of proprietary data in the Bayesian model affected the estimated number of filings. As discussed above, there are many structural challenges to collecting a complete set of eviction records from courts. Limitations imposed on record collection and changes in collection volume may create trends in the proprietary data that do not reflect true fluctuations in case filings. As the proprietary data entered the Bayesian model as a secondary, imperfect measure of court-issued filings, it was also possible to estimate the model without these data. Structurally, the model omitting the proprietary data was very similar to the model described in Section 3. The model included the same covariates and used filing counts from the court-issued data as the primary outcome.

As shown in Fig. S24, filing rates were estimated to be 4.7% higher, on average, when excluding proprietary data from the model. The discrepancy was greatest in 2000 before stabilizing at a smaller margin for the remaining years. The estimates from the full model incorporating proprietary data were contained within the 95% credible intervals of the modeling omitting the proprietary data in many years between 2009 and 2016, albeit near the lower bound. While the estimates from the model without proprietary data were consistently higher, we could not conclude that the estimates differ significantly in these years. The shape of the longitudinal trend for filings was almost identical regardless of whether proprietary data were included in the model. The curvilinear trend in eviction filings then is not attributable to fluctuations in secondary collection volume over time. Additionally, the 95% credible intervals were much wider when proprietary data was not included in the model. Even if sometimes incomplete, the proprietary data provided an important signal about how many filings should be expected in areas without court-issued data. In particular, the proprietary estimates were a valuable source of information when we lacked court-issued data for entire states. Filing rates differ substantially across states, even when populations are demographically similar (Fig. S10). For this reason, observing data from as many states as possible is crucial to producing accurate estimates of the national prevalence of eviction.

The importance of access to data in all states is clear when comparing predicted state-level filings with and without proprietary data. In the Northeast, the availability of court-issued data increased the reliability of estimates over time (Fig. S25); however, the inclusion of proprietary data helped reduce uncertainty in states with long-term limited data, particularly New York. In the Midwest, we observed similar reductions in uncertainty in Illinois and Kansas (Fig. S26). Reductions in uncertainty were even more striking in the South (Fig. S27), where lack of court-issued data in Louisiana, Mississippi, Oklahoma, Tennessee, and West Virginia made it particularly difficult to estimate filings in this region. This pattern was repeated in Idaho and Montana in the West (Fig. S28). Incorporating proprietary data into the analyses was key to producing more precise estimates, without creating artificial patterns in long-term filing trends.

5.3 Maryland Estimates

Maryland is an outlier in the number of eviction filings. An average of 589,064 cases (95% credible interval: 579,879 - 597,957) were filed annually between 2000 and 2018. With a renting population of approximately 707,000 households, this is an average annual filing rate of 83.3%. In some areas of the state, including Baltimore City, the annual number of filings regularly exceeds the number of renting households (*30*). The unusually high filing rate is produced by the state's landlord-tenant policy. In Maryland, the filing serves as the initial eviction notice and can be filed immediately following nonpayment (*31*); however, if the tenant pays the balance of the rent due, plus any incurred late fees or court costs, the complaint is considered satisfied and the tenant remains in place (*32*, *33*). For this reason, many households receive multiple filings before an actual eviction occurs; in fact, repeated filings against the same household constituted 57.4% of all case filings in Maryland.

We have annual county-level, court-issued aggregated landlord-tenant filing counts in Maryland for 2000-2017. These data are released in annual reports by the Maryland Judiciary (*34*). The only source of uncertainty in our filing counts then is adjusting for the portion of eviction-specific cases out of the total landlord-tenant case volume (see Section 3) in 2017 and estimates for 2018. As aggregated filing counts do not include case-specific information, we had to estimate households threatened with eviction from the Bayesian model (Section 5). The uniquely high prevalence of filings in Maryland resulted in higher estimated rates of households threatened with eviction, as we might expect.

To understand how the high prevalence of filings in Maryland affected national estimates, we produced an additional set of numbers that excluded Maryland. Excluding Maryland reduced the number of filings by approximately 500,000 cases annually. This reduced the average national filing rate by 14.5%, from 9.0% to 7.7% (Panel A, Fig. S29). Although the filing prevalence was lower when excluding Maryland, the overall longitudinal trend in the filing rate remained unchanged, indicating that, while Maryland does contribute a large number of filings in the national perspective, it is not the primary driver of changes in the trajectory of eviction prevalence.

Maryland's effect on the rates of households threatened with eviction was less substantial (Panel B, Fig. S29). When excluding Maryland, the average rate of households threatened with eviction was reduced by 7.7%. The relatively smaller magnitude of this reduction is likely attributable to the higher prevalence of repeat filings against the same households. The "pay to stay" condition allows renters to remain in the residence if financial obligations of the eviction complaint are satisfied, yet the use of the filing as the eviction notice and low filing fees incentivize the landlords to repeatedly file cases if rent payments are late in subsequent months. In this way, unique households may represent a smaller portion of the annual filings. Renting households in Maryland are at higher risk of filings, independent of household characteristics typically associated with eviction (Fig. S10). They are also at higher risk of receiving repeated filings (Fig. S11), which is why excluding Maryland results in a larger reduction to the filing rate than rate of households threatened with eviction.

The process for notifying a tenant of an eviction action, including how and when the landlord is required to provide notice, differs significantly across states but is not included in many classifications of state-level landlord-tenant policy regimes. The frequent classification of Maryland as a more tenant-friendly state (e.g., *35*) may reflect important legal protections afforded to renters but does not capture key aspects of policies that create increased risk of landlords threatening tenants with court-ordered eviction. These differences contribute significantly to disparities in eviction

filings across states and deserve greater attention in future studies of landlord-tenant policy.

6 Eviction Notice Requirements

We investigated the association between state-level eviction notice requirements for filing nonpayment of rent cases and filing rates by downloading the publicly available Eviction Laws Database released by the Legal Services Corporation (LSC) (*36*). We used two measures of eviction notice requirements from the data. First, an indicator for whether landlords were required to provide notice to tenants before filing an eviction case for nonpayment of rent. All 50 states and DC require landlords to provide notice for eviction filings for reasons other than nonpayment, but, as nonpayment cases constitute a substantial majority of filings, the lack of notice for nonpayment cases is more consequential for overall filing volume. Second, the number of days' notice, if any, a landlord was required to provide prior to filing a nonpayment case.

The LSC Eviction Laws Database was assembled in 2021, after the period for which we had collected data on eviction filings. To verify that the same notice requirements were in place for the 2000-2018 period, we checked the legal code history using the Westlaw Next Campus Research database (accessed through the Princeton University Library). We searched for archived copies of the legal codes cited by LSC for eviction notice requirements. When possible, we verified the consistency of notice requirements for the full period of study. We examined the text of the most recent archived legal code prior to 2000 and any amendments enacted between 2000 and 2018 (Table S7). The verification year listed in Table S7 does not represent the date the law(s) governing notice requirements were implemented or changed; instead, it represents the most recent year prior to the start of the study period (2000) that archived enacted legal code was available. Most notice requirements were initially adopted well before the year used to verify the legal code as of 2000. Westlaw served as the verification source, except when noted otherwise in Table S7. We were able to verify notice requirements for the full study period in 36 states. For the remaining 15 states, we were able to verify requirements for 13 (out of 19 possible) years, on average. Only 11 of these 15 states had counties included in the analysis. The lack of verification for the full study period does not mean that notice requirements changed during this time, only that archived legal code was not readily available pre-dating 2000. In fact, we observed no changes to notice requirements during the 2000-2018 period. This indicates that policies governing notice requirements tend to be fairly stable over time. As shown in Table S7, we did find 8 discrepancies between notice requirements in the LSC Eviction Laws Database and the archived legal code, all owing to changes in the law that occurred post-2018. In all cases, we were able to observe the changes directly in archived legal code. Some of these changes were due to the COVID-19 pandemic (e.g., in DC), while others may have been attributable to increasing attention to eviction as an important housing issue in the US.

The lack of changes in eviction notice requirements during our study period prohibited us from examining the associations between these requirements and filing rates longitudinally within states. If we included required days notice in models with state-level fixed effects (which help account for time-invariant factors that may be associated with both housing policy regimes and eviction filing frequency), the effects of notice requirements would be absorbed into the (unobserved) state-specific intercepts. To overcome these difficulties, we exploited state variation occurring in core-based statistical areas (CBSAs) that cross state borders. CBSAs are designed to capture an urban area and the surrounding counties that are socioeconomically interdependent with that urban area. Unlike many Census areal units that are fully contained within states, CBSAs are defined as groupings of counties that are not bound by state borders. CBSAs constitute areas expected to share similar socio-demographic and economic conditions; however, eviction filings are governed

by the landlord-tenant code of the state in which the county is located. This variation allowed us to examine differences in eviction notice requirements for nonpayment cases in otherwise comparable areas.

We used a regression discontinuity design to detect the associations between notice requirements and filing rates across state borders. We placed two restrictions on our analytic sample. First, we included only counties with validated court-issued eviction filing counts. We restricted the sample in this way to eliminate concerns that the covariates used to predict filing counts in the Bayesian model may be correlated with state policy conditions, thus inflating the association between notice requirements and filing rates. Second, the county had to be located in a CBSA that crossed state borders. We included cross-state CBSAs in the sample regardless of whether notice requirements changed at the state border; CBSAs without variation in notice requirements were necessary to help estimate the associations (*37*). Our sample included 230 counties in 39 CBSAs that met these criteria. Fig. S13 shows the counties included in the sample and the distribution of notice requirements across states. Table S8 provides a list of the CBSAs included in the sample.

We fit a longitudinal linear regression model with random effects at the county level and fixed effects for year and CBSA. The random effects were appropriate for repeated observations within counties across time. Including fixed effects at the year level helped account for any exogenous variation in filing rates over time. CBSA fixed effects helped control for local time-invariant conditions that may be associated with the likelihood of eviction filings. The outcome was the logged eviction filing rate. We calculated this by dividing the number of court-issued filings by the total number of renting households in the county and then taking the natural log. We added a small constant (0.01) to avoid dropping the few county-years in which the filing rate was zero. Excluding these counties did not affect the results. We logged the filing rate to adjust for skew (i.e., the majority of counties had very low filing rates, while a few consistently had more filings than renting households).

The primary covariate of interest was a categorical measure of required days notice for nonpayment filings. Although the original measure was interval (number of days), we created a categorical measure due to concerns that the association between days required notice and eviction filings may not be linear; for example, additional days' notice near the beginning of the interval (e.g., one to three days) may be more consequential to the filing rate than additional days near the end of the interval (e.g., 12-14 days). We separated days' notice into four categories:

1. 0 days (i.e., no notice required)
2. 1-3 days
3. 4-7 days
4. 8-14 days

We tested alternative specifications of these categories (e.g., 0 days, 1-6 days, 7-13 days, and 14 or more days), but chose the shorter windows due to the lack of states requiring more than 14 days' notice prior to nonpayment filings. We entered this measure into the model as a series of indicators to reflect the assumed non-linearity in association between notice period and filing rate. Descriptive statistics for this measure (and all others included in the analysis) are shown in Table S17. The models contained several additional control variables. First, we included the county-level

socio-demographic variables from the Bayesian model of case filings: percent renting households, share African American population, household density, median household income, median rent, and unemployment rate. We logged household density, median household income, and median rent to adjust for skew (Model 1 in Table S9). We also fit a model that included the number of courts that hear eviction cases, but this did not substantively change the association between notice requirements and filing rate (Model 2 in Table S9). We did not include this model in Fig. 4 due to space considerations.

We then included a series of policy measures capturing other aspects of the landlord-tenant legal environment that may affect how often landlords file eviction cases. First, we used an indicator for whether a state requires that landlords have a just cause to terminate tenancy at the end of a lease. This measure was also taken from the LSC Eviction Laws Database (*36*). Although this requirement itself may not substantially impact on the number of eviction filings, it may help capture the general character of the landlord-tenant legal regime (*35*). We also included the fee required to file an eviction case (see Section 1.3.13) as a measure of the relative ease (or difficulty) of case filing. Filing fees were consistent across all counties in 28 states and tended to be very similar across counties in states that did not have fixed fee schedules (Table S6). In general, lower filing fees were associated with higher eviction filing rates (Fig. S30). Finally, we included a state-level indicator for whether filings were eviction-specific or represented all landlord-tenant cases. In practice, discrepancies between these counts are very minor as most landlord-tenant filings are eviction cases (see Section 3.2) but this indicator also helped to verify that any associations with filing rates were not due to the inclusion of non-eviction complaints in the filing counts. While filing fee and landlord-tenant indicators were both significantly associated with filing volume, they did not explain the association between notice requirements and filing rates (Model 3 in Table S9).

Finally, we fit two additional models with more stringent restrictions on sample inclusion to test the robustness of these associations. These additional restrictions imposed criteria expected to increase comparability of the counties included within CBSAs. First, we restricted the sample to counties that were located directly along the state border. This restricted our sample to 168 counties in the 39 CBSAs but did not substantively alter the estimated associations between notice requirements and filing rates (Model 4 in Table S9). Second, we restricted the sample to counties that had population centers located within 25 miles of the state border (*38*). Again, this resulted in a smaller number of counties in the sample (N=185) but no substantive change in the findings (Model 5 in Table S9).

We also tested the robustness of the specification of notice requirements. We fit an identical set of models that used a binary indicator of required notice (1=landlords required to provide notice to tenants before filing a nonpayment case, 0=no notice required); these results are shown in Fig. S31. As expected, the indicator was negative and significant across all models.

In practice, the number of days required notice before filing usually approximates the minimum number of days a tenant can be late on rent before the landlord may initiate an eviction lawsuit. To demonstrate this, we created an equivalent categorical measure of the number of days a tenant can be late on rent before having a case filed against them from the LSC Eviction Laws Database (*36*). We updated this measure for any changes in legal code that occurred between our study period and the creation of the LSC dataset (Table S7). This measure produced almost identical findings (Fig. S32), suggesting that policies governing the timing of eviction filings by landlords, including grace periods for late rent payments, are significantly associated with how frequently eviction cases are filed, even after accounting for local demographic and socioeconomic conditions.

These analyses do not establish the causal effect of notice requirements for nonpayment of rent on eviction filings, but they suggest that state-level landlord-tenant legal code can play an important role in shaping risk of an eviction filing. The estimated associations are also substantially large. This is likely due to the high filing rates observed in states that do not require prior notice before filing eviction cases for nonpayment of rent or do not have a minimum grace period tenants can fulfill past-due rent before receiving an eviction lawsuit. This has important practical implications. Landlord-tenant policies that incentivize landlords to legally document that tenants are past-due on rent or to use the courts to enforce rent collection (*4*) can put tenants at higher risk of receiving an eviction filing. This complicates commonly held beliefs that eviction filing prevalence is a straightforward reflection of unpaid rent. Two states could have similar proportions of tenants with outstanding rent balances but very different rates of eviction filings. In states that require landlords to provide a minimum period of notice (or a grace period for unpaid rent), tenants may be less at risk of receiving the permanent mark of an eviction filing in their housing history. As mentioned in the main text, eviction filings, not just orders to vacate rental properties or executed evictions by the local sheriff, are recorded in housing histories, limiting future access to rental housing (*39*).

Additional research is necessary to establish the causal effects of specific landlord-tenant policies on frequency of eviction filings and risk of displacement due to eviction. Eviction notice requirements may be associated with other aspects of state-level landlord-tenant legal code that shape risk of being threatened with eviction; these policy regimes may affect this risk in ways that extend far beyond the demographic or socio-economic factors that have been identified as increasing risk of receiving an eviction filing or forced displacement due to eviction. Maryland and New Jersey, states that have been frequently categorized as “renter-friendly” in previous studies of landlord-tenant legal regimes (e.g., *35*), consistently report higher than average filing rates and do not require landlords to provide tenants with notice prior to nonpayment filings (nor do they specify a minimum number of days a tenant can be late on rent before receiving an eviction filing), demonstrating that there is much work to be done in understanding the characteristics and effects of landlord-tenant policy regimes.

7 Eviction Judgments

In this paper, we measured eviction prevalence as the number of eviction filings and households threatened with eviction annually. While these numbers illuminate the burden of eviction on the court system and the threat eviction poses to housing security, they do not tell us how many households are displaced by eviction. This number is more difficult to estimate for many reasons. First, court records do not provide information about whether tenants vacated the rental property before, during, or after an eviction filing. When multiple cases were filed against the same household, we knew that tenants remained at the property after the previous filing but did not know whether the case series ultimately resulted in displacement. For households who only appeared once in the data, we had no means of assessing whether the single filing resulted in displacement. Second, courts do not uniformly record whether writs of restitution were issued for the disputed property following a judgment in favor of the landlord. Forcible removal of tenants by the sheriff or marshal is perhaps the strictest definition of an eviction (*31*) yet counting only these events as evictions ignores the many tenants who vacate properties as a direct result of legal eviction action before a writ of restitution is granted or executed. Third, while a judgment in favor of the landlord restores legal authority over the property and/or grants the ability to extract past-due rent and fees from tenants, court records often contain unclear information about the terms of the judgment and whether landlords ultimately sought restitution of the property. Furthermore, the quality of this information and how it is recorded varies across court records, making direct comparisons of the volume of eviction judgments very difficult.

Most of the individual records data (both court-issued and proprietary) contained some information about how cases were resolved. The court-issued data often contained the judgment codes used by the courts; some were detailed enough to determine whether restitution of property to the landlord was included in the judgment, while others used only broad categories of judgments (e.g., bench verdict, consent judgment, default judgment, mediated settlement). Only a few states directly recorded which party the judgment favored. In some states, codes were too uninformative or ambiguous to determine whether the judgment was in favor of the landlord or tenant. The proprietary data, on the other hand, did not contain court judgment codes but marked judgments as small claims, civil judgments, or forcible detainer judgments in favor of the plaintiff. Small claims and civil judgments typically indicate that defendants were ordered to pay the plaintiff a monetary settlement, while forcible detainers involve restitution of property to the landlord. It was not possible to determine whether restitution of property was included in the small claims or civil judgments, which constituted the overwhelming majority of judgments in the data. Furthermore, 32.1% of cases in the proprietary data did not contain judgment information. This judgment information was not missing at random. Some dispositions, including dismissals, were less likely to be recorded in the data. Additionally, some states were less likely to have judgment information included for cases, including Georgia, New Jersey, and Utah.

We attempted to develop a systematic marking and tabulation of eviction judgments across data sources and states but found little agreement between court-issued and proprietary data when comparing aggregated judgment counts. There could be several reasons for these discrepancies. Lack of clear judgment information in some of the court-issued data made it difficult to mark eviction judgments in a way that was consistent with the judgment categories included in the proprietary data. The lack of original court judgment codes in the proprietary data made it impossible to reconstruct the information available in the court-issued data. To be clear, neither the court-issued

nor the proprietary data were created with the purpose of tracking displacement due to eviction, underscoring the limitations of using administrative data to answer this type of research question. Furthermore, the proprietary data recorded the current case status at the point of data collection. If judgments or other actions occurred on the case following the date of collection, information may not be updated in these data. This may explain why some cases lack judgment information: when the case information was collected the case was still pending. The court-issued data would likely reflect the most up-to-date information on case status, although some court-issued data files contained non-trivial numbers of cases missing judgment information as well.

Although we were unable to create a comprehensive measure of eviction judgments, we calculated this measure for the court-issued individual records to provide a general sense of how many case filings resulted in eviction-related judgments. We first provide details about how we marked eviction judgments in the court-issued data. We then compare aggregated eviction judgments across 16 states. Although the court-issued data have more complete judgment information than the proprietary data, missing and ambiguous judgment information in the court-issued data still limits the ability to accurately determine how cases were resolved.

7.1 Marking Eviction Judgments

When possible, we marked cases as resulting in an eviction judgment if the final action recorded for a case was a judgment in favor of the plaintiff. Judgment information varied significantly across the court-issued data files. Some files included information on which party the judgment was in favor of or included straightforward judgment codes indicating unlawful detainer or monetary judgments. When this information was not included in the data, we marked judgment types likely to be in favor of the plaintiff (e.g., default, consent, and uncontested) as eviction judgments. For cases without clear judgment information, it was impossible to discern how the case was resolved. These cases could represent unresolved cases or cases in which there was a judgment (for eviction or otherwise), but it was not entered into the case management system. We did not count cases lacking judgment information as resulting in eviction judgments. We were able to observe case filings and some form of judgement information from court-issued data for 7,002 county years in the following states and counties:

- Alabama
- Maricopa County, Arizona
- San Francisco County, California
- Connecticut
- DC
- Hawaii
- Cook and Kane Counties, Illinois
- Iowa
- Johnson and Wyandotte Counties, Kansas

- Minnesota
- Missouri
- Nebraska
- New York City (Bronx, Kings, New York, Queens, Richmond)
- North Dakota
- South Dakota
- Virginia

In some instances, we observed an eviction judgment issued against a household but knew it did not result in displacement because the same household was observed at the same property on a subsequent case filing. In states and counties with complete name and address information for records, we could adjust for this. We created a secondary measure of eviction judgments in which we counted a case as having an eviction judgment only if it was the final case observed in the series of filings against the same household. We did not count eviction judgments on the non-final cases in the series as we knew the previous judgments did not result in displacement. This adjustment did not affect households that appeared once in the data, as one case represented their first and last filing. We were able to calculate this secondary measure for 6,549 county years in the following states and counties:

- Alabama
- Maricopa County, Arizona
- Hawaii
- Cook and Kane Counties, Illinois
- Iowa
- Minnesota
- Missouri
- Nebraska
- New York City (Bronx, Kings, New York, Queens, Richmond)
- North Dakota
- Virginia

We could not create this secondary measure for San Francisco County, Connecticut, DC, Kansas, or South Dakota due to missing name and address information. Although included in the adjusted measures, partial name and address missingness in Maricopa County, Arizona and Virginia may have resulted in incomplete adjustment for repeated filings against tenants.

7.2 Eviction Judgment Prevalence

Fig. S33 shows the average rate of case filings ending in eviction judgments across states. In seven states more than 50% of the cases filed ended in eviction judgments, while eviction judgments were less common in other jurisdictions. For example, less than 25% of cases filed in DC ended in eviction judgments, suggesting that more cases may be dismissed, judged in favor of the defendant, or remain unresolved. Adjusting for repeated filings against the same household also produced larger reductions in the eviction judgment rate in some municipalities, including New York City and Virginia, relative to others. This suggests that filings result in different rates of eviction judgments across states and that some repeated filings result in eviction judgments that do not routinely lead to displacement of tenants.

Fig. S34 highlights these differences by plotting comparisons between average rates of case filings, households threatened with eviction, and adjusted eviction judgments. Filings in North Dakota and Hawaii are more likely to represent cases against unique households and, in the case of North Dakota, eviction judgments than other states. New York and North Carolina have high filing rates, yet comparatively average eviction judgment rates. It is difficult then to make assumptions about the number of eviction judgments or displacement due to eviction based on the number of filings or unique households threatened with eviction in a state. Certainly, higher rates of filings are often associated with repeating cases against the same households, but some states still have relatively higher percentages of filings ending in eviction judgments than others, even after accounting for repeated filings. The differences in filing behavior by landlords across states are not clearly communicated in eviction records, leaving tenants in states with high filing rates with relatively inflated numbers of eviction cases in their housing histories.

Although these numbers are restricted in scope, these 11 states and 11 counties recorded an average of 342,139 eviction judgments annually. This is an average of 53.7% of filings resulting in eviction judgments (*40*). If these states are representative of the rate of filings resulting in eviction judgments in the national perspective, more than 1.9 million eviction judgments would be issued on eviction cases, on average, in the US each year. The magnitude of case filings, households receiving at least one filing, and eviction judgments annually demand better accounting of displacement due to eviction across the US, including work that acknowledges that filing rates differ systematically across states and do not accurately capture households displaced due to eviction.

8 Supplementary Tables

Table S1. State and county court-issued individual records

	Geographic Area	Years	Raw Records	Raw Cases	Cleaned Cases[1]
States	Alabama	1961-2015[10]	1,148,022	503,663	355,992
	Connecticut	2006-2016[11]	333,029	219,809	211,575
	Washington DC[2]	2002-2013	504,424	504,380	502,364
	Hawaii	2006-2016	28,955	25,736	22,160
	Indiana[3]	1983-2016	366,703	152,361	148,426
	Iowa	1995-2015	374,419	283,699	225,458
	Minnesota	2009-2016[11]	414,393	153,246	137,242
	Missouri	2011-2015	534,782	220,958	218,397
	Nebraska	1986-2015[10]	857,687	136,349	117,999
	New Jersey[4]	1995-2015	1,638,451	713,958	
	New York[5]	2000-2018[11]	5,420,999	4,918,190	4,483,504
	North Carolina	2006-2015	4,438,982	1,838,724	1,838,166
	North Dakota	1986-2017[10,11]	31,920	19,904	12,790
	Oregon	1978-2016	760,410	529,582	311,449
	Pennsylvania	2006-2017[12]	2,089,888	876,283	543,018
	South Carolina	1990-2015	2,591,894	2,591,894	2,106,432
	South Dakota	2010-2017	27,242	5,596	4,610
	Virginia[6]	2010-2017[11]	1,237,430	1,226,473	1,201,666
	Washington[7]	2004-2013	200,163	200,163	266,652
Counties	Maricopa (AZ)	2000-2017	1,266,536	1,266,536	1,203,003
	San Francisco (CA)	2000-2019	77,805	69,031	66,393
	Johnson (KS)[8]	2016-2017	4,625	4,625	4,625
	Wyandotte (KS)[8]	2016-2017	4,519	4,519	4,518
	Cook (IL)	2001-2015	1,536,686	487,745	463,221
	Kane (IL)	2006-2015	44,472	25,789	22,875
	Orleans Parish (LA)	2006-2014[11]	64,816	24,282	22,805
	Philadelphia (PA)[9]	1969-2016[10]	680,053	679,961	350,008
	Total		26,679,305	17,683,456	14,533,899

[1] Cases filed from 2000-2018, with duplicated and commercial cases removed (analytic sample).
[2] Data downloaded from: http://www.grahamimac.com/dcevictions.
[3] Data was only available for the 27 counties (of 92 total) included in Indiana's Odyssey case management system. Of these 27 counties, data were incomplete for 16 counties, leaving only 11 counties included in the analysis.
[4] Data from New Jersey was too incomplete and inconsistent to be cleaned and aggregated.
[5] Data were only available for the five counties in New York City: Bronx, Kings, New York, Queens, and Richmond.
[6] Data collected via web-scraping and shared by Ben Schofield (http://virginiacourtdata.org/).
[7] Data collected and shared by Tim Thomas.
[8] Data collected and shared by Flatland (*41*).
[9] Data collected via web-scraping and shared by Jonathan Pyle (https://www.philalegal.org/jonathanpyle).
[10] Many early years included only partial data.
[11] Partial-year data only for final year.
[12] Partial data only for first and final years.

Table S2A. Aggregated county-level court-issued eviction filings reported by state courts

State	Number of Counties	Counties Included	Years	Total Cases
Alabama	67	67	2000-2017	384,883
Alaska	29	28	2007-2016	28,108
Arizona	15	15	2004-2017	1,271,394
Arkansas	75	75	2000-2018	53,547
California	58	57-58	2010-2017	1,413,453
Colorado	64	64	2001-2017	793,894
Connecticut	8	8	2016-2018	62,358
Delaware	3	3	2003-2018	269,043
Washington DC	1	1	2003-2018	627,903
Florida	67	67	2000-2018	2,729,944
Georgia	159	159	2000-2016	4,211,932
Hawaii	5	5	2004-2018	36,352
Kentucky	120	120	2000-2016	671,399
Maine	16	16	2006-2018	70,515
Maryland	24	24	2000-2017	10,608,785
Massachusetts	14	14	2002-2016	568,247
Michigan	83	83	2010-2018	1,870,533
Minnesota	87	87	2017-2018	35,164
Missouri	115	115	2003-2017	664,869
Nebraska	93	91	2000-2016	155,018
Nevada	17	17	2000-2016	657,189
New Hampshire	10	10	2000-2018	158,520
New Jersey	21	21	2013-2018	989,211
New Mexico	33	33	2000-2018	294,013
New York	62	42	2017-2018	541,917
North Carolina	100	100	2000-2018	3,268,604
North Dakota	53	53	2000-2016	15,810
Ohio	88	88	2002-2018	1,877,027
Oregon	36	30-36	2000-2018	443,521
Pennsylvania	67	67	2000-2018	2,098,079
Rhode Island	5	5	2008-2018	102,352
South Dakota	66	56-57	2010-2016	4,609
Texas	254	254	2000-2018	3,879,252
Utah	29	29	2004-2018	116,213
Vermont	14	14	2009-2018	15,395
Virginia	134	124	2014-2016	509,999
Washington	39	39	2014-2018	266,652
Wisconsin	72	72	2000-2017	482,930
Wyoming	23	23	2014-2017	5,501
Total				42,254,135

Table S2B. Aggregated county-level court-issued eviction filings reported by county courts

State	County	Years	Total Cases
California	Los Angeles	2002-2009	433,509
	San Francisco	2005-2009	19,030
Indiana	Allen	2017-2018	10,606
	Wayne	2000-2017	41,473
	Whitley	2000-2017	774
Louisiana	Vernon	2000-2017	496
Illinois	Bond	2000-2017	413
	Cumberland	2017	1
	Edwards	2001-2017	15
	Madison	2000-2017	21,899
	Montgomery	2000-2017	1,936
	Morgan	2000-2017	1,701
	Moultrie	2010-2017	144
	Piatt	2001-2017	160
	Rock Island	2000-2017	14,864
Mississippi	Harrison	2017-2018	16,516
Montana	Park	2003-2018	681
New York	Bronx	2009-2016	666,860
	Kings	2009-2016	568,128
	New York	2009-2016	449,148
	Queens	2009-2016	320,134
	Richmond	2009-2016	43,240
Oklahoma	Bryan	2000-2017	5,022
	Dewey	2000-2017	83
	Stephens	2000-2017	3,747
Tennessee	Macon	2008-2018	826
Total			2,621,406

Table S3. County-years of excluded eviction filing counts in court-issued data, 2000-2018

	Individual Records		Aggregated Filings	
State	Total	Excluded	Total	Excluded
Alabama	1,139	0	1,205	10
Alaska	0	0	280	0
Arizona	17	1	210	0
Arkansas	0	0	1,425	1,072
California	19	0	474	3
Colorado	0	0	1,080	5
Connecticut	77	7	24	0
Delaware	0	0	45	0
Washington DC	12	0	16	0
Florida	0	0	1,273	6
Georgia	0	0	2,703	450
Hawaii	45	0	70	0
Illinois	25	0	134	40
Indiana	158	88	38	0
Iowa	1,584	4	0	0
Kansas	4	0	0	0
Kentucky	0	0	2,040	117
Louisiana	0	0	26	0
Maine	0	0	208	0
Maryland	0	0	432	1
Massachusetts	0	0	210	0
Michigan	0	0	747	0
Minnesota	609	0	174	0
Mississippi	0	0	2	0
Missouri	575	12	1,725	8
Montana	0	0	16	0
Nebraska	1,183	1	1,547	5
Nevada	0	0	289	0
New Hampshire	0	0	190	0
New Jersey	0	0	126	0
New Mexico	0	0	627	8
New York	85	6	124	84
North Carolina	1,000	4	1,800	5
North Dakota	689	0	901	0
Ohio	0	0	1,496	16
Oklahoma	0	0	54	0
Oregon	612	612	662	16
Pennsylvania	413	2	1,273	0
Rhode Island	0	0	55	0
South Carolina	736	49	0	0
South Dakota	371	3	398	0
Tennessee	0	0	11	1
Texas	0	0	4,824	429
Utah	0	0	435	4
Vermont	0	0	140	6
Virginia	868	0	372	0
Washington	0	0	578	1
Wisconsin	0	0	1,296	15
Wyoming	0	0	92	0

Table S4. Source of reported county-level eviction filing counts, 2000-2018

	Court-issued Records		Court-issued Aggregates		Bayes Posterior Distribution	
Year	Counties	% Renting HH[1]	Counties	% Renting HH	Counties	% Renting HH
2000	211	9.92	932	27.78	2,000	62.30
2001	213	13.19	1,073	31.14	1,857	55.67
2002	215	14.23	1,186	41.71	1,742	44.06
2003	304	13.43	1,234	39.47	1,605	47.09
2004	364	14.98	1,288	47.92	1,491	37.10
2005	364	14.91	1,322	48.48	1,457	36.61
2006	470	18.94	1,246	46.31	1,427	34.75
2007	479	19.41	1,267	46.15	1,397	34.44
2008	475	14.96	1,249	46.42	1,419	38.62
2009	568	20.74	1,287	47.19	1,288	32.08
2010	814	26.45	1,355	55.51	974	18.04
2011	926	28.16	1,246	53.94	971	17.89
2012	928	28.10	1,234	53.91	981	18.00
2013	930	28.04	1,257	56.73	956	15.23
2014	931	27.70	1,275	56.43	937	15.87
2015	929	27.60	1,298	56.88	916	15.52
2016	307	11.93	1,677	66.51	1,159	21.56
2017	3	0.74	1,407	61.95	1,733	37.31
2018	1	0.52	982	39.93	2,160	59.54
Total	9,432	17.57	23,815	49.23	26,470	33.20

[1] The percent of total US renting households represented by the counties covered in each data source.

Table S5. Estimated Bayesian coefficients for eviction filing counts, socio-demographic covariates

			95% Credible Interval	
Variable	Mean	Std. Dev.	Lower Bound	Upper Bound
Renting households	1.07	0.02	1.03	1.10
Low number renting households (< 1600)	-0.09	0.01	-0.11	-0.07
Household density	0.54	0.04	0.47	0.62
Number of eviction courts	0.10	0.01	0.07	0.12
Percent Black population	0.07	0.01	0.06	0.09
Percent Black population over 40%	0.01	0.00	0.00	0.02
Median income	0.03	0.00	0.02	0.04
Median rent	0.04	0.01	0.02	0.06
Median rent > $1300	-0.01	0.00	-0.01	0.00
Unemployment rate	0.00	0.01	-0.01	0.02

Table S6. Distribution of required fees to file an eviction case across states, 2018

State	No. Counties	Mean	Std. Dev.	Min	Max
Alabama	62	277.23	26.33	221.50	350.00
Alaska	29	150.00	0.00	150.00	150.00
Arizona	15	100.53	10.50	89.00	119.00
Arkansas	75	165.00	0.00	165.00	165.00
California	58	240.78	4.37	240.00	270.00
Colorado	64	97.00	0.00	97.00	97.00
Connecticut	8	205.00	0.00	205.00	205.00
Delaware	3	45.00	0.00	45.00	45.00
Washington DC	1	15.00		15.00	15.00
Florida	67	185.00	0.00	185.00	185.00
Georgia	159	89.66	20.55	60.00	224.00
Hawaii	5	155.00	0.00	155.00	155.00
Idaho	44	166.00	0.00	166.00	166.00
Illinois	101	143.67	39.71	67.00	328.00
Indiana	91	100.37	17.14	76.00	197.00
Iowa	99	85.00	0.00	85.00	85.00
Kansas	105	54.14	1.46	54.00	69.00
Kentucky	118	123.38	12.55	81.00	142.50
Louisiana	64	100.09	3.07	86.00	120.00
Maine	16	100.00	0.00	100.00	100.00
Maryland	24	15.42	2.04	15.00	25.00
Massachusetts	14	205.29	30.81	171.00	231.00
Michigan	83	55.00	0.00	55.00	55.00
Minnesota	86	295.51	3.52	285.00	300.00
Mississippi	80	64.84	3.32	62.50	89.00
Missouri	115	51.97	17.63	33.00	150.00
Montana	56	50.00	0.00	50.00	50.00
Nebraska	93	46.00	0.00	46.00	46.00
Nevada	17	56.88	11.56	25.00	76.00
New Hampshire	10	150.95	0.15	150.54	151.00
New Jersey	21	50.00	0.00	50.00	50.00
New Mexico	33	77.00	0.00	77.00	77.00
New York	62	45.00	0.00	45.00	45.00
North Carolina	100	126.00	0.00	126.00	126.00
North Dakota	53	80.00	0.00	80.00	80.00
Ohio	88	127.79	30.22	60.00	205.00
Oklahoma	77	108.00	0.00	108.00	108.00
Oregon	36	83.00	0.00	83.00	83.00
Pennsylvania	65	162.10	20.03	131.32	228.00
Rhode Island	5	100.75	0.00	100.75	100.75
South Carolina	46	40.00	0.00	40.00	40.00
South Dakota	66	70.00	0.00	70.00	70.00
Tennessee	93	135.29	34.25	72.50	208.50
Texas	244	131.71	21.41	48.00	246.00
Utah	29	75.00	0.00	75.00	75.00
Vermont	14	295.00	0.00	295.00	295.00
Virginia	133	44.47	2.68	40.00	58.00
Washington	39	85.00	0.00	85.00	85.00
West Virginia	55	50.00	0.00	50.00	50.00
Wisconsin	72	94.55	0.41	94.50	98.00
Wyoming	23	55.00	0.00	55.00	55.00

Table S7. Verification of eviction notice requirements for nonpayment filings, 2000-2018

State	Days' Notice Required	Year Verified	Year Changed	Notes
Alabama	7	2006		
Alaska	7	1994		
Arizona	5	1999		
Arkansas	3	2005		
California	3	2001		
Colorado	3	2005	2019	Earliest enactment date with same text was 2005, although no amendments from 1994-2005 included changes to notice requirements. Notice requirements were amended to 10 days in 2019.
Connecticut	3	1999		
Delaware	5	1996		
Washington DC	0	1990	2021	Verified in D.C. Annotated Code. There were no documented changes to notice requirements in legal code archived by Westlaw since 1990. Notice requirements were temporarily instated due to COVID-19 in 2021.
Florida	3	1999		
Georgia	1	1995		
Hawaii	5	1984		
Idaho	3	2001		
Illinois	5	2011		
Indiana	10	2002		
Iowa	3	1979		
Kansas	3	1992		
Kentucky	7	1984		
Louisiana	5	1981		
Maine	7	1999		
Maryland	0	2004		Hartman & Robinson (2003) also noted lack of required notice in 2002 filings.
Massachusetts	14	1977		
Michigan	7	1990		
Minnesota	0	1999		
Mississippi	3	1972		Verified in Mississippi Annotated Code indexed by LexisNexis.
Missouri	10	1997		
Montana	3	1995		Verified in Montana Annotated Code.
Nebraska	3	2001	2019	Changed to 7 days in 2019.
Nevada	5	1985	2019	Changed to 7 days in 2019.
New Hampshire	7	1988		Westlaw lists statute as valid through 2006, previous amendment was dated 1988.
New Jersey	0	1997		
New Mexico	3	1999		
New York	3	1985	2019	
North Carolina	10	2001		
North Dakota	3	2009		
Ohio	3	2006		Verified in Ohio Annotated Code.
Oklahoma	5	1995		
Oregon	6	2005	2020	Increased to 13 days in 2020.
Pennsylvania	10	1996		
Rhode Island	5	1986		
South Carolina	5	1998		
South Dakota	3	1986		
Tennessee	14	1999		
Texas	3	1997		
Utah	6	2006		Source: https://law.justia.com/codes/utah/2006/title78/78_32004.html; guide from Utah Legal Services in 1996 also specified 3 days' notice requirement.
Vermont	14	1999		
Virginia	5	2000	2020	Changed to 14 days in 2020.
Washington	3	1998	2020	Changed to 14 days in 2020.
West Virginia	0	1983		
Wisconsin	5	2012		
Wyoming	3	1977		

Table S8. Core-based statistical areas (CBSAs) in eviction notice requirements analyses

Core-Based Statistical Area	County-years		
	Full Model	State Border	Within 25 Miles of State Border
Allentown-Bethlehem-Easton, PA-NJ	63	25	44
Augusta-Richmond County, GA-SC	103	87	103
Berlin, NH-VT	29	29	29
Boston-Cambridge-Newton, MA-NH	113	83	83
Charlotte-Concord-Gastonia, NC-SC	181	89	124
Chicago-Naperville-Elgin, IL-IN-WI	45	35	35
Cincinnati, OH-KY-IN	203	169	186
Claremont-Lebanon, NH-VT	58	58	58
Columbus, GA-AL	69	57	69
Davenport-Moline-Rock Island, IA-IL	34	34	34
Duluth, MN-WI	36	36	27
Eufaula, AL-GA	31	31	31
Evansville, IN-KY	33	33	33
Fargo, ND-MN	26	26	26
Fayetteville-Springdale-Rogers, AR-MO	52	33	33
Fort Madison-Keokuk, IA-IL-MO	31	31	31
Grand Forks, ND-MN	26	26	26
Huntington-Ashland, WV-KY-OH	51	51	51
Iron Mountain, MI-WI	27	27	27
Kansas City, MO-KS	139	79	49
La Crosse-Onalaska, WI-MN	27	27	27
Louisville/Jefferson County, KY-IN	119	56	107
Marinette, WI-MI	27	27	27
Minneapolis-St. Paul-Bloomington, MN-WI	162	63	90
Myrtle Beach-Conway-North Myrtle Beach, SC-NC	35	35	35
New York-Newark-Jersey City, NY-NJ-PA	170	146	164
Omaha-Council Bluffs, NE-IA	132	115	115
Philadelphia-Camden-Wilmington, PA-NJ-DE-MD	152	133	152
Portland-Vancouver-Hillsboro, OR-WA	120	68	101
Providence-Warwick, RI-MA	70	70	70
Salisbury, MD-DE	69	69	69
Sioux City, IA-NE-SD	73	73	73
South Bend-Mishawaka, IN-MI	11	11	11
St. Louis, MO-IL	123	93	93
Virginia Beach-Norfolk-Newport News, VA-NC	122	59	80
Wahpeton, ND-MN	26	26	26
Washington-Arlington-Alexandria, DC-VA-MD-WV	205	138	131
Worcester, MA-CT	28	28	28
Youngstown-Warren-Boardman, OH-PA	53	53	53
Total	3,074	2,329	2,551

Table S9. Estimated associations of eviction notice requirements for nonpayment cases on logged eviction filing rate, 2000-2018

	(1)			(2)			(3)		
Variable	County Demos.			County Courts			State Policy		
	Coeff.	SE	p	Coeff.	SE	p	Coeff.	SE	p
Renting households (%)	-0.009	0.005	0.060	-0.008	0.005	0.109	-0.011	0.005	0.022
Household density (log.)	0.126	0.040	0.002	0.093	0.041	0.023	0.151	0.038	0.000
Black pop. (%)	0.023	0.004	0.000	0.031	0.004	0.000	0.023	0.003	0.000
Median income (log.)	0.013	0.146	0.928	-0.062	0.159	0.695	-0.046	0.145	0.752
Median rent (log.)	0.218	0.128	0.088	0.295	0.138	0.033	0.243	0.128	0.057
Unemployment rate (%)	0.003	0.007	0.694	-0.003	0.006	0.592	0.000	0.007	0.997
No. eviction courts				-0.007	0.003	0.014			
Just cause (1=yes)							-0.366	0.200	0.067
Filing Fee ($)							-0.004	0.001	0.000
Landlord-tenant (1=yes)							-0.222	0.058	0.000
Notice Required									
0 days/No notice		*(ref.)*			*(ref.)*			*(ref.)*	
1-3 days	-0.573	0.171	0.001	-0.602	0.179	0.001	-0.997	0.205	0.000
4-7 days	-0.538	0.148	0.000	-0.517	0.147	0.000	-0.838	0.157	0.000
8-14 days	-0.966	0.166	0.000	-0.937	0.164	0.000	-1.184	0.195	0.000
Constant	0.808	1.463	0.580	1.311	1.480	0.376	2.184	1.454	0.133
County-years	3,074			2,570			3,074		
Counties	230			222			230		

SE = standard error; p = p-value. Fixed effects included for year and CBSA but not shown. The percentage reductions in eviction filing rates shown in Fig. 4 were generated by exponentiating the coefficients shown in the table above, subtracting 1, and multiplying by 100 (a standard method to calculate percentage change in the non-transformed outcome).

Table S9. Estimated associations of eviction notice requirements for nonpayment cases on logged eviction filing rate, 2000-2018 (cont.)

Variable	(4) Located on State Border			(5) Within 25 mi. of Border		
	Coeff.	SE	p	Coeff.	SE	p
Renting households (%)	-0.017	0.006	0.002	-0.016	0.005	0.003
Household density (log.)	0.198	0.046	0.000	0.189	0.043	0.000
Black pop. (%)	0.022	0.004	0.000	0.022	0.004	0.000
Median income (log.)	-0.026	0.178	0.884	-0.029	0.163	0.859
Median rent (log.)	0.162	0.153	0.291	0.149	0.145	0.302
Unemployment rate (%)	0.006	0.008	0.446	0.004	0.007	0.612
Just cause (1=yes)	-0.440	0.217	0.043	-0.374	0.203	0.066
Filing Fee ($)	-0.004	0.001	0.000	-0.004	0.001	0.000
Landlord-tenant (1=yes)	-0.044	0.082	0.591	-0.191	0.067	0.005
Notice Required						
0 days/No notice		*(ref.)*			*(ref.)*	
1-3 days	-0.977	0.226	0.000	-0.932	0.214	0.000
4-7 days	-0.818	0.177	0.000	-0.770	0.169	0.000
8-14 days	-1.190	0.216	0.000	-1.127	0.202	0.000
Constant	2.062	1.823	0.258	2.481	1.642	0.131
County-years	2,329			2,551		
Counties	168			185		

SE = standard error; p = p-value. Fixed effects included for year and CBSA but not shown. The percentage reductions in eviction filing rates shown in Fig. 4 were generated by exponentiating the coefficients shown in the table above, subtracting 1, and multiplying by 100 (a standard method to calculate percentage change in the non-transformed outcome).

Table S10. Yearly assignment of Census and American Community Survey data for covariates

Year	Data Source
2000	
2001	
2002	2000 Census
2003	
2004	
2005	
2006	
2007	2005-2009 5-Year American Community Survey
2008	
2009	
2010	2010 Census (short form) & 2008-2012 5-Year American Community Survey
2011	
2012	
2013	2011-2015 5-Year American Community Survey
2014	
2015	
2016	
2017	2014-2018 5-Year American Community Survey
2018	

Table S11. Comparison of eviction filing counts, court-issued to proprietary data

State	County-years	Court = Prop.	Court ≈ Prop.	Court < Prop.	Court > Prop.	Mean % Diff.
Alabama	1,204	6.73	61.21	3.07	28.99	-13.07
Alaska	19	0.00	0.00	0.00	100.00	-76.65
Arizona	174	0.00	10.92	0.57	88.51	-60.05
Arkansas	114	0.88	45.61	2.63	50.88	-82.56
California	406	0.49	7.14	0.49	91.87	-54.06
Colorado	901	4.33	39.18	5.44	51.05	-38.85
Connecticut	66	0.00	6.06	0.00	93.94	-30.28
Delaware	45	0.00	42.22	2.22	55.56	-19.26
Washington DC	17	0.00	17.65	0.00	82.35	-42.78
Florida	1,138	0.00	9.49	7.56	82.95	-47.17
Georgia	1,761	0.74	27.09	4.09	68.09	-28.86
Hawaii	44	0.00	0.00	0.00	100.00	-64.57
Illinois	112	3.57	33.04	17.86	45.54	-20.90
Indiana	107	2.80	25.23	24.30	47.66	-11.47
Iowa	1,519	12.84	61.09	1.65	24.42	-22.84
Kansas	4	0.00	25.00	0.00	75.00	-25.71
Kentucky	1,397	2.65	37.01	1.36	58.98	-43.19
Louisiana	21	0.00	19.05	52.38	28.57	22.00
Maine	205	0.00	12.68	0.00	87.32	-30.25
Maryland	425	0.00	0.00	0.00	100.00	-92.59
Massachusetts	198	0.51	19.19	1.01	79.29	-40.18
Michigan	722	0.69	14.82	1.39	83.10	-42.65
Minnesota	616	8.93	43.34	6.17	41.56	-22.74
Mississippi	2	0.00	0.00	0.00	100.00	-62.25
Missouri	1,640	8.60	56.95	10.85	23.60	-7.77
Montana	15	6.67	26.67	0.00	66.67	-39.76
Nebraska	863	14.83	59.33	2.32	23.52	-28.86
Nevada	213	1.88	23.94	3.76	70.42	-36.55
New Hampshire	141	0.00	0.00	0.00	100.00	-73.42
New Jersey	126	0.00	23.02	1.59	75.40	-8.15
New Mexico	548	2.55	33.94	5.66	57.85	-31.29
New York	74	0.00	39.19	5.41	55.41	-30.69
North Carolina	1,883	4.41	48.75	11.15	35.69	-9.53
Ohio	1,473	2.38	49.15	3.80	44.67	-12.06
Oklahoma	53	7.55	28.30	5.66	58.49	-20.15
Oregon	607	3.62	49.92	11.70	34.76	-0.50
Pennsylvania	553	3.62	59.49	0.36	36.53	-19.74
Rhode Island	43	0.00	4.65	0.00	95.35	-48.41
South Carolina	344	1.16	24.71	1.45	72.67	-26.68
Tennessee	10	0.00	10.00	0.00	90.00	-31.05
Texas	2,801	2.28	25.24	1.07	71.40	-56.37
Utah	331	5.14	45.92	1.21	47.73	-35.75
Vermont	82	0.00	10.98	0.00	89.02	-90.90
Virginia	859	2.21	25.73	4.89	67.17	-25.63
Washington	513	2.34	31.58	1.56	64.52	-34.90
Wisconsin	1,262	7.37	62.28	8.40	21.95	-5.98
Wyoming	64	1.56	46.88	0.00	51.56	-66.09
Total	25,715	4.27	38.66	4.60	52.47	-29.78

Note: “Court = Prop.” = exact match of court-issued and proprietary data; “Court ≈ Prop.” = court-issued and proprietary data not an exact match but difference was less than 10 cases or 5% of filings reported in court-issued data; “Court < Prop.” = court-issued data reported fewer cases than proprietary data (difference was more than 10 cases or 5% of filings in court-issued data); “Court > Prop.” = court-issued data reported more cases than proprietary data (difference was more than 10 cases or 5% of filings in court-issued data); Mean. % diff is average percentage difference in court-issued and proprietary filing counts for county-years with more than 10 filings reported in the court-issued data.

Table S12. Covariate transformations for Bayesian model

Variable	First Transformation	Second Transformation
Number of renting households	log(max(1500,x) + 1)	log(max(22016, x) + 10)
Household density	log(x + 1)	
Number of eviction courts	log(x + 1)	
Black/African American pop. (%)	log(x + 1)	x > 40
Median income	log(min(20000, max(75000, x)))	
Median rent	log(x + 1)	x > 1300
Unemployment rate (%)	log(x + 1)	

Table S13. Descriptive statistics for Bayesian models, county-level, 2000-2018

Variable	N	Mean	Std. Dev.	Minimum	Maximum
Court Data					
Number of case filings[1]	33,247	1,526.11	7,929.07	0.00	176,701.00
Number of unique households in filings[2]	4,711	304.25	1,809.72	0.00	35,442.00
Proprietary Data					
Number of case filings	44,953	906.28	4,430.34	1.00	143,719.00
Number of unique households in filings	44,953	721.88	3,189.29	0.00	74,854.00
Percent auto-collected cases	44,953	22.71	39.96	0.00	100.00
Percent dismissed cases	44,953	6.02	12.90	0.00	100.00
Percent unresolved cases	44,953	19.04	25.53	0.00	100.00
Number of uploads	46,586	18.14	33.10	0.00	340.00
Demographics					
Number of renting households	59,717	12,957.53	51,494.53	5.00	1,824,095.00
Household density	59,717	112.38	818.83	0.03	38,823.26
Median income	59,709	43,619.56	12,776.40	9,333.00	136,268.00
Median rent	59,698	616.14	210.07	178.00	2,158.00
Percent African American population	59,717	8.80	14.41	0.00	87.41
Unemployment rate	59,635	6.12	2.71	1.10	28.90
Number of eviction courts	58,558	1.87	2.57	1.00	46.00
**Total County-Years**	59,717				

[1] From court-issued individual records and aggregated filing counts.
[2] From court-issued individual records with complete name and address information.

Table S14. Longitudinal trends in court-issued filing counts, state-level

State	Average Filing Rate Period 1 2000-2003	Period 2 2006-2010	Period 3 2015-2018	Percent Change Period 1 to 2	Period 2 to 3
Alabama	3.79	3.64	4.01	-3.86	10.13
Alaska		3.03	2.77		-8.30
Arizona		12.62	8.59		-31.94
California		4.01	2.47		-38.31
Colorado	9.04	8.87	5.91	-1.87	-33.36
Connecticut		4.75	4.34		-8.81
Delaware	17.03	19.29	16.45	13.28	-14.69
Washington DC	31.96	27.62	18.14	-13.59	-34.31
Florida	6.72	6.63	4.48	-1.34	-32.51
Georgia	22.85	23.68	18.89	3.65	-20.25
Hawaii		1.28	1.14		-10.79
Iowa	4.24	4.47	3.80	5.42	-14.89
Kentucky	8.32	7.70	7.02	-7.51	-8.77
Maine		3.10	3.11		0.35
Maryland	80.29	87.03	83.33	8.40	-4.25
Massachusetts	3.45	4.00	3.93	16.03	-1.92
Michigan		19.57	17.11		-12.55
Minnesota		3.77	2.82		-25.06
Missouri	5.56	6.36	5.60	14.48	-12.00
Nebraska	4.05	4.03	3.33	-0.59	-17.32
Nevada	12.38	9.16	9.37	-26.00	2.24
New Hampshire	4.90	5.97	4.72	21.80	-20.92
New Jersey			12.96		
New Mexico	5.84	6.96	6.16	19.26	-11.51
North Carolina	17.48	16.07	11.64	-8.05	-27.59
North Dakota	0.76	0.76	1.44	-0.33	89.92
Ohio	7.52	7.67	6.38	2.05	-16.78
Oregon	5.15	4.51	3.05	-12.48	-32.45
Pennsylvania	6.79	7.43	7.14	9.39	-3.90
Rhode Island		5.30	5.80		9.34
South Carolina	24.29	26.65	23.67	9.75	-11.21
South Dakota		0.52	0.67		28.31
Texas	5.56	6.79	6.39	22.05	-5.84
Utah		3.20	2.38		-25.71
Vermont		1.99	2.15		8.05
Virginia		18.40	15.03		-18.30
Washington		2.14	1.92		-10.43
Wisconsin	3.62	3.84	3.62	6.20	-5.74
Wyoming			1.71		
Unweighted mean				3.31	-10.06
Weighted mean				5.60	-18.76
**National estimate (Fig. 1)**	**9.08**	**9.58**	**8.14**	**5.56**	**-15.06**

Note: Blank cells indicate missing data within that period. We only included state-years in calculations if we had data for at least 50% of counties to increase likelihood that trends were representative of the state overall. This excluded ten states: Arkansas, Illinois, Indiana, Kansas, Louisiana, Mississippi, Montana, New York, Oklahoma, and Tennessee.

Table S15. Comparison of eviction filing counts, court-issued to Bayesian posterior estimates

	All County-years					County-years with > 10 court-issued filings	
State	County-years	Court ≈ Ests.	Court < Ests.	Court > Ests.	Mean % Diff.	County-years	Mean % Diff.
Alabama	1,205	64.84	17.74	17.41	5.56	1,128	1.52
Alaska	231	89.29	5.71	5.00	25.20	103	-2.16
Arizona	213	41.31	30.52	28.17	7.69	203	2.10
Arkansas	328	88.10	4.53	7.37	28.95	114	-3.69
California	477	39.41	34.17	26.42	6.82	465	2.48
Colorado	1,025	64.84	16.93	18.23	15.69	757	0.80
Connecticut	94	39.36	39.36	21.28	2.33	94	2.33
Delaware	45	66.67	17.78	15.56	0.41	45	0.41
Washington DC	17	64.71	29.41	5.88	1.68	17	1.68
Florida	1,262	37.17	31.81	31.02	5.93	1,241	3.61
Georgia	2,249	37.64	32.80	29.56	4.79	2,211	3.78
Hawaii	56	48.57	28.57	22.86	2.42	56	2.42
Illinois	109	62.18	14.29	23.53	12.87	87	-2.91
Indiana	108	37.96	25.00	37.04	10.23	106	3.26
Iowa	1,568	80.25	8.23	11.52	13.08	1,107	-0.66
Kansas	4	50.00	50.00	0.00	2.33	4	2.33
Kentucky	1,921	63.75	17.68	18.56	10.51	1,689	2.89
Louisiana	26	61.54	15.38	23.08	6.72	26	6.72
Maine	208	48.56	25.96	25.48	2.40	208	2.40
Maryland	431	25.06	36.66	38.28	0.81	431	0.81
Massachusetts	210	35.71	33.81	30.48	5.30	210	5.30
Michigan	746	50.47	28.25	21.29	4.22	731	3.24
Minnesota	780	74.58	12.01	13.41	14.38	594	0.77
Mississippi	2	50.00	0.00	50.00	-4.77	2	-4.77
Missouri	1,691	72.39	12.00	15.61	13.38	1,300	0.12
Montana	16	56.25	25.00	18.75	6.81	16	6.81
Nebraska	1,274	90.73	3.50	5.77	30.18	460	-5.87
Nevada	272	53.98	23.18	22.84	12.21	214	3.61
New Hampshire	190	32.11	38.95	28.95	2.66	190	2.66
New Jersey	126	55.56	34.13	10.32	2.26	126	2.26
New Mexico	585	56.87	22.13	21.00	9.10	500	3.13
New York	79	32.91	43.04	24.05	2.32	79	2.32
North Carolina	1,895	50.77	28.34	20.90	2.85	1,882	2.32
North Dakota	522	91.79	4.33	3.88	20.25	158	-2.38
Ohio	1,480	45.68	30.07	24.26	3.06	1,476	2.66
Oklahoma	54	61.11	14.81	24.07	29.12	36	-1.29
Oregon	605	51.70	24.30	23.99	10.07	538	3.07
Pennsylvania	1,273	60.09	23.17	16.73	3.60	1,227	1.78
Rhode Island	55	21.82	45.45	32.73	3.13	55	3.13
South Carolina	687	29.84	39.74	30.42	3.78	687	3.78
South Dakota	218	96.74	1.50	1.75	24.58	47	-11.61
Tennessee	10	20.00	50.00	30.00	6.42	10	6.42
Texas	3,917	64.35	17.52	18.13	13.65	3,005	1.33
Utah	396	74.48	11.60	13.92	18.67	256	-1.66
Vermont	134	63.43	18.66	17.91	11.39	119	1.62
Virginia	868	50.12	27.65	22.24	4.92	851	2.80
Washington	570	60.14	21.14	18.72	8.27	496	2.39
Wisconsin	1,257	68.54	14.05	17.41	7.84	1,140	0.97
Wyoming	89	77.17	2.17	20.65	29.03	50	-4.74
Total	33,247	60.47	20.37	19.16	10.04	26,547	1.84

Note: "Court ≈ Ests." = Difference between court-issued filing counts and Bayesian estimates ("Ests.") was less than 10 cases or 5% of filings reported in court-issued data; "Court < Ests." = court-issued data reported fewer cases than Bayesian estimates (difference was 10 or more cases or greater than 5% of court-issued filings); "Court > Ests." = court-issued data reported more cases than Bayesian estimates (difference was 10 or more cases or greater than 5% of court-issued filings); Mean. % diff is average percentage difference in court-issued filing counts and Bayesian estimates.

Table S16. Average state-level eviction filing rates, by data source, 2015

Individual Court-Issued Records N = 11		Aggregated Court-Reported Filing Counts N = 25		Bayesian Posterior Estimates N = 10		No Data Source ≥ 90% of Counties N = 5	
State	Filing Rate	State	Filing Rate	State	Filing Rate	State	Filing Rate
Alabama	3.96	Alaska	2.80	Idaho	2.95	Arkansas	0.85
Hawaii	1.12	Arizona	8.60	Illinois	2.91	Connecticut	4.69
Iowa	3.80	California	2.66	Kansas	3.57	Indiana	8.74
Minnesota	2.80	Colorado	5.81	Louisiana	2.51	Oregon	3.22
Missouri	5.39	Delaware	17.33	Mississippi	16.25	South Dakota	0.60
Nebraska	3.27	Washington DC	19.02	Montana	1.43		
North Carolina	11.76	Florida	4.85	New York	9.42		
North Dakota	1.37	Georgia	18.99	Oklahoma	6.19		
Pennsylvania	6.99	Kentucky	6.96	Tennessee	6.88		
South Carolina	23.67	Maine	3.28	West Virginia	5.71		
Virginia	16.05	Maryland	83.53				
		Massachusetts	3.93				
		Michigan	17.98				
		Nevada	9.33				
		New Hampshire	5.28				
		New Jersey	14.06				
		New Mexico	6.21				
		Ohio	6.68				
		Rhode Island	5.33				
		Texas	6.22				
		Utah	2.51				
		Vermont	2.13				
		Washington	1.99				
		Wisconsin	3.59				
		Wyoming	1.67				
Mean	7.29		10.43		5.78		3.62
Minimum	1.12		1.67		1.43		0.60
Maximum	23.67		83.53		16.25		8.74

Table S17. Descriptive statistics for eviction notice requirement models, county-level, 2000-2018

Variable	N	Mean	Std. Dev.	Minimum	Maximum
Eviction filing rate	3,074	9.70	13.64	0.00	136.42
Eviction filing rate (logged)	3,074	1.72	1.09	-4.61	4.92
Nonpayment notice required (1=yes)	3,074	0.86		0.00	1.00
Days' notice	3,074				
0 days (no notice)	461				
1-3 days	722				
4-7 days	1,077				
8-14 days	814				
Renting households (%)	3,074	26.82	11.02	5.86	76.26
Household density	3,074	734.11	3,072.17	3.02	38,394.05
Black/African American pop. (%)	3,074	10.62	13.63	0.00	63.52
Hispanic/Latino pop. (%)	3,074	6.09	7.11	0.00	55.89
Median household income	3,074	55,333.29	16,565.08	25,101.00	136,268.00
Median rent	3,074	795.50	271.63	315.00	1,936.00
Unemployment rate (%)	3,074	6.07	2.33	2.00	19.80
Number of eviction courts	2,570	2.13	3.07	1.00	35.00
Just cause requirement (1=yes)	3,074	0.10		0.00	1.00
Filing Fee ($)	3,074	108.39	73.48	15.00	328.00
Landlord-tenant filings (1=yes)	3,074	0.30		0.00	1.00

9 Supplementary Figures

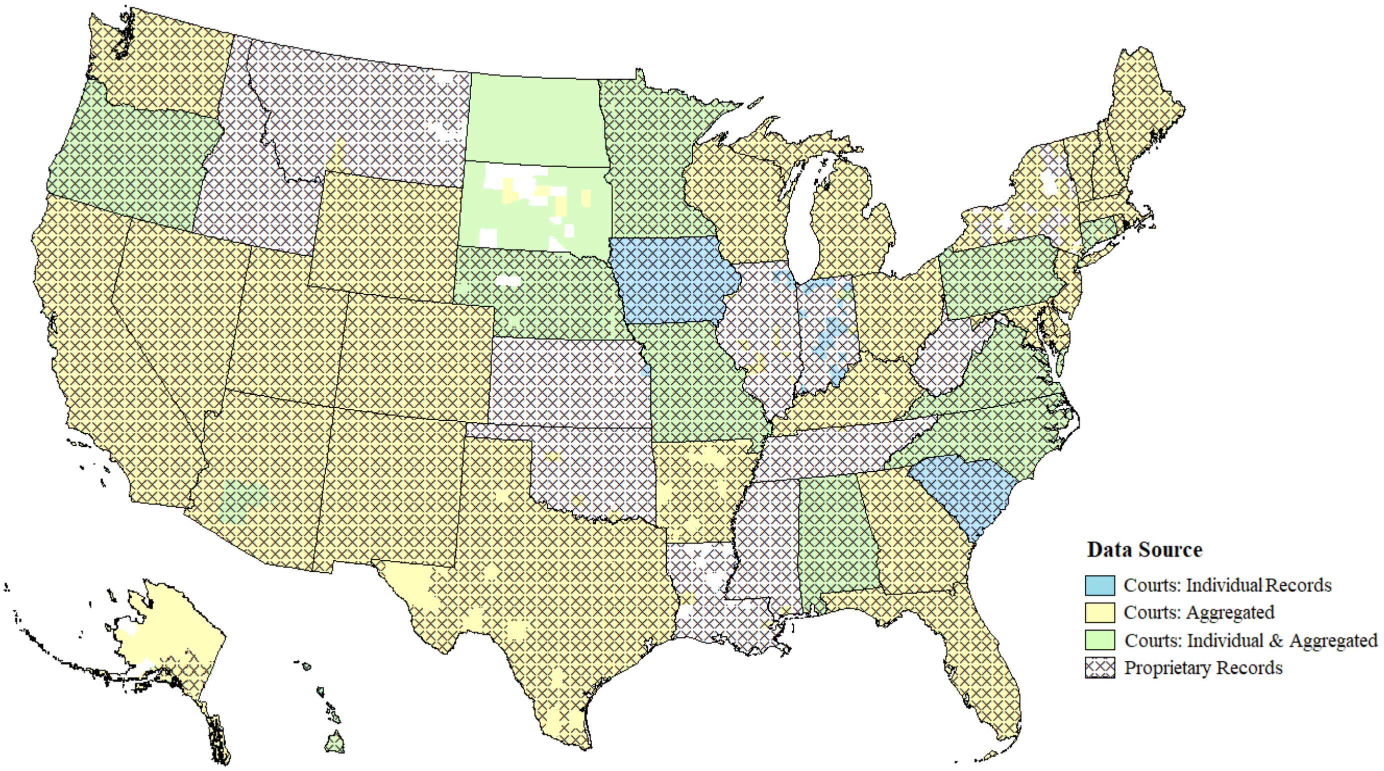

Fig. S1. County-level data coverage across the United States
Coverage is shown based on one or more years of county-level data from that source. Section 2 provides details of data validation, including where data were determined to be incomplete and excluded from the analyses.

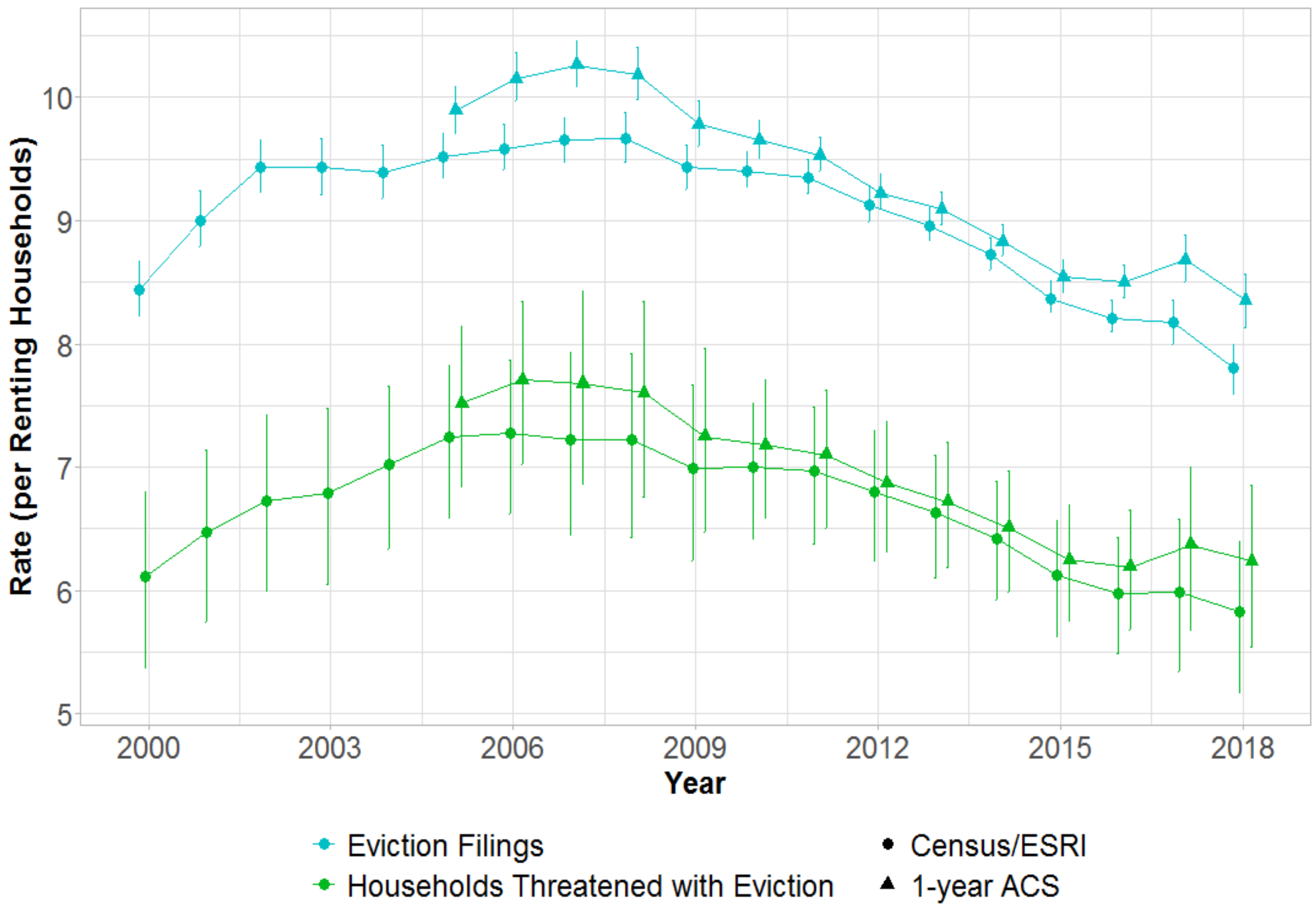

Fig. S2. Rates of eviction filings and households threatened with eviction, by tenure source, 2000-2018.

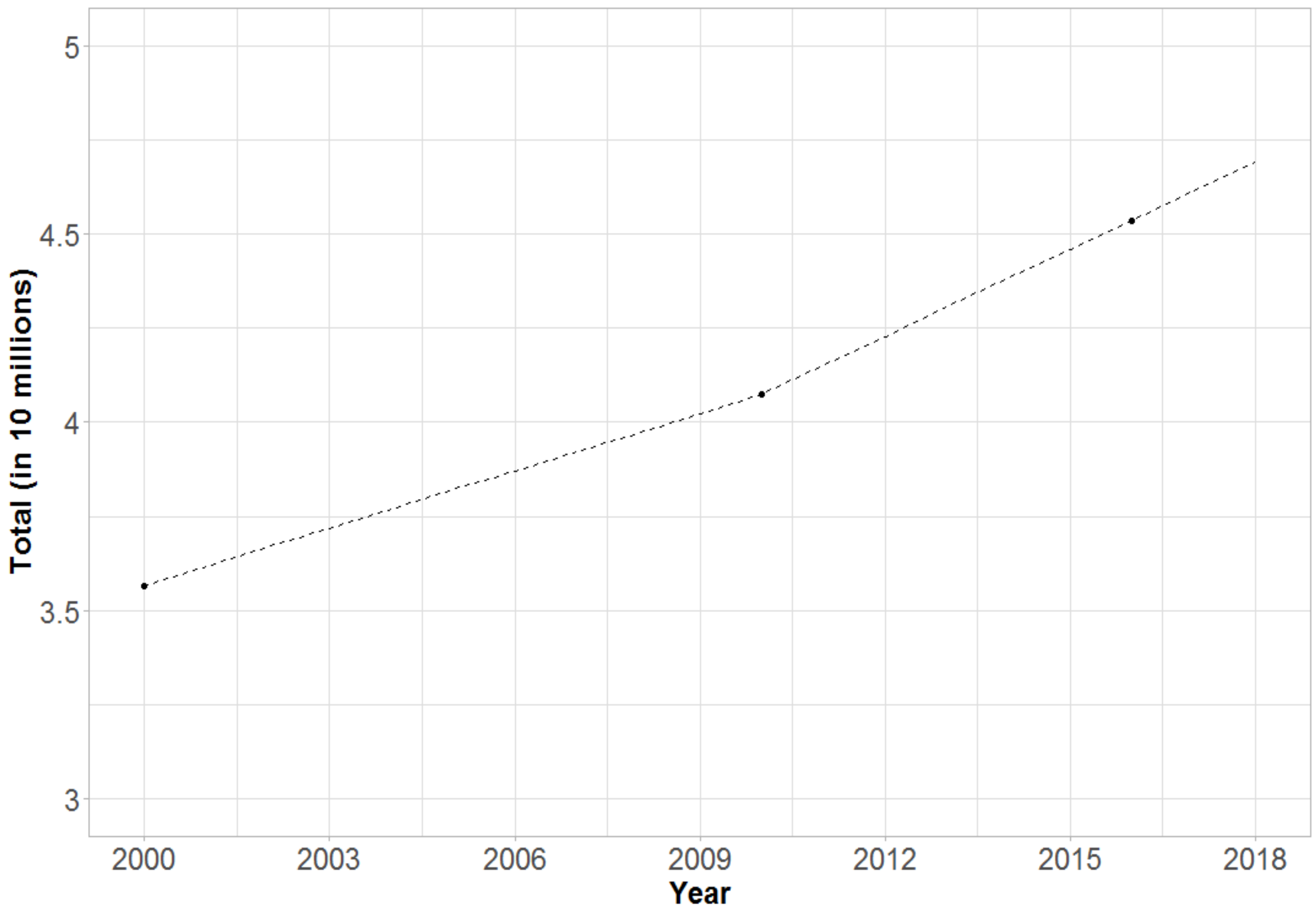

Fig. S3. Number of renting households in the United States, 2000-2018.
Estimates of renting households were taken from the 2000 and 2010 Censuses and 2016 ESRI Business Analyst. We linearly interpolated estimates of renting households from 2000 to 2010, 2010 to 2016, and 2016 to 2018, as shown by the dashed line.

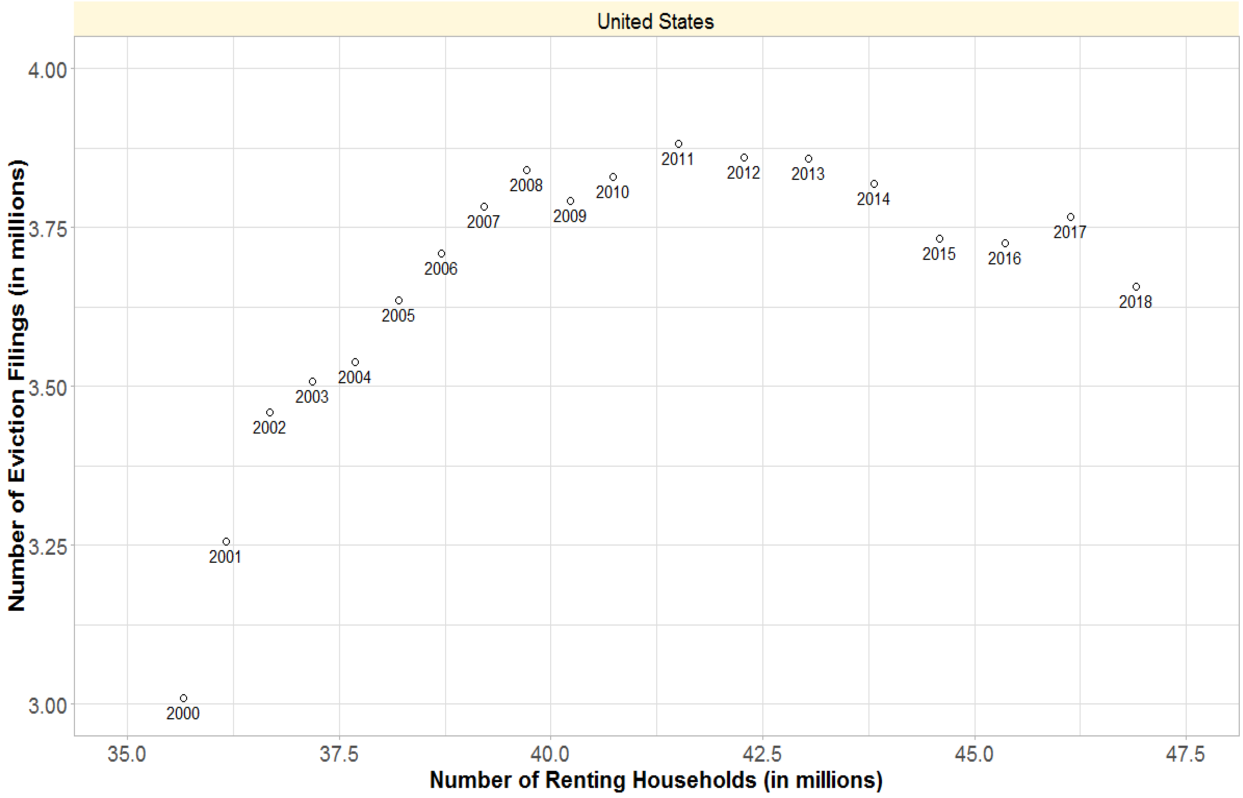

Fig. S4. Annual number of eviction filings and renting households, United States, 2000-2018

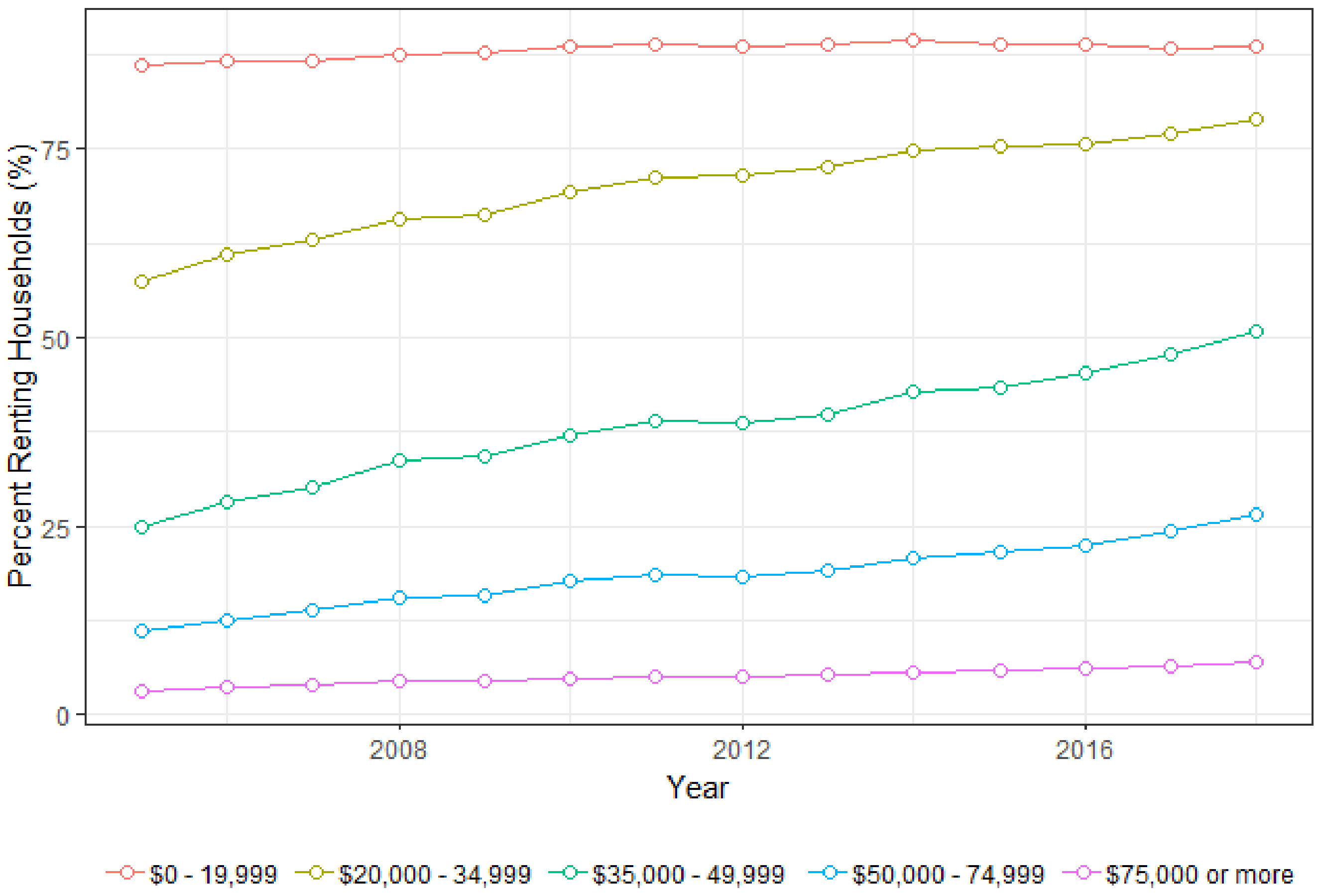

Fig. S5. Renting households spending 30% or more on housing costs, by income, 2005-2018
Source: 1-year American Community Survey, 2005-2018 (Table B25106)

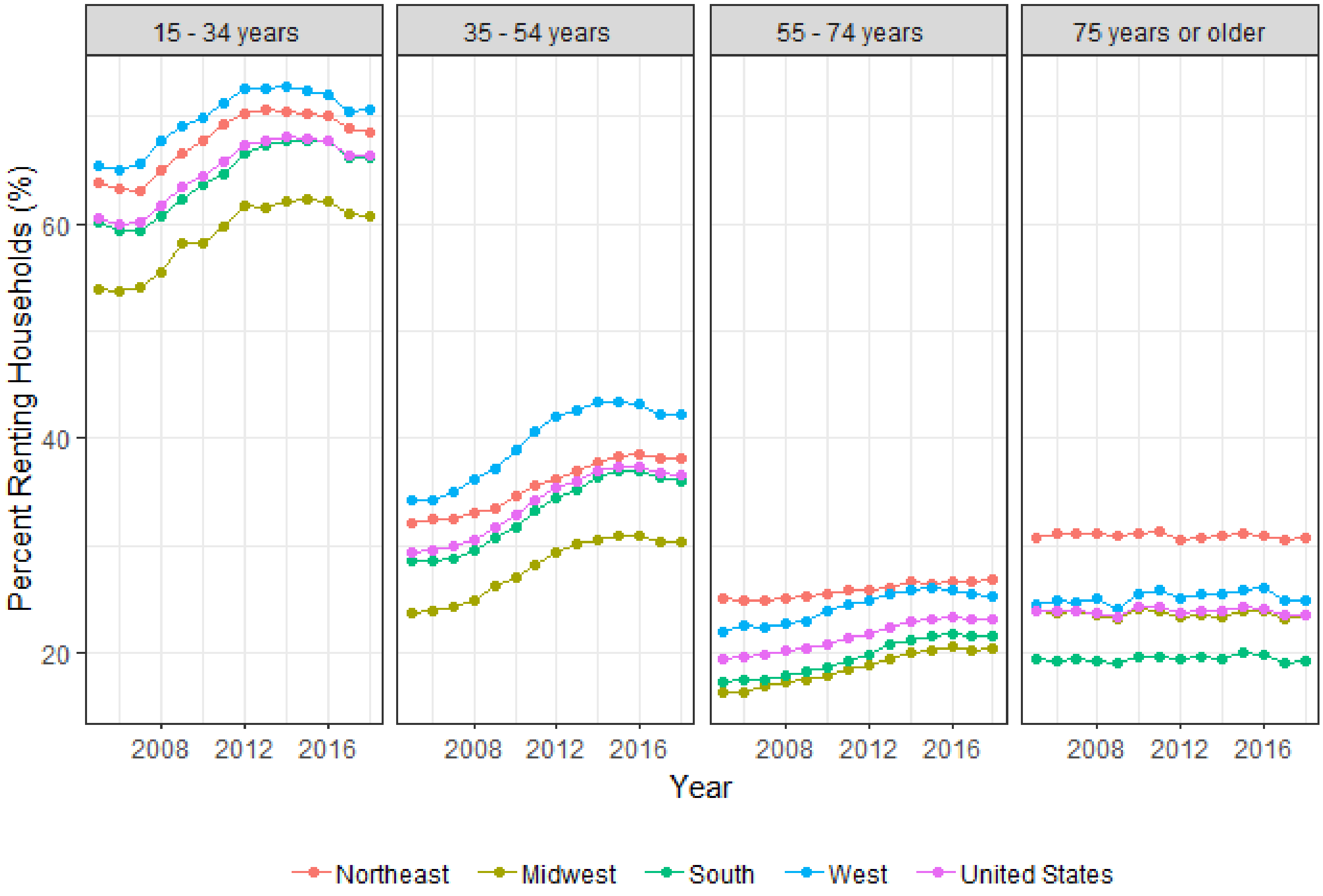

Fig. S6. Percent renting households by age of householder and region, 2000-2018
Source: 1-year American Community Survey, 2005-2018 (Table B25007)

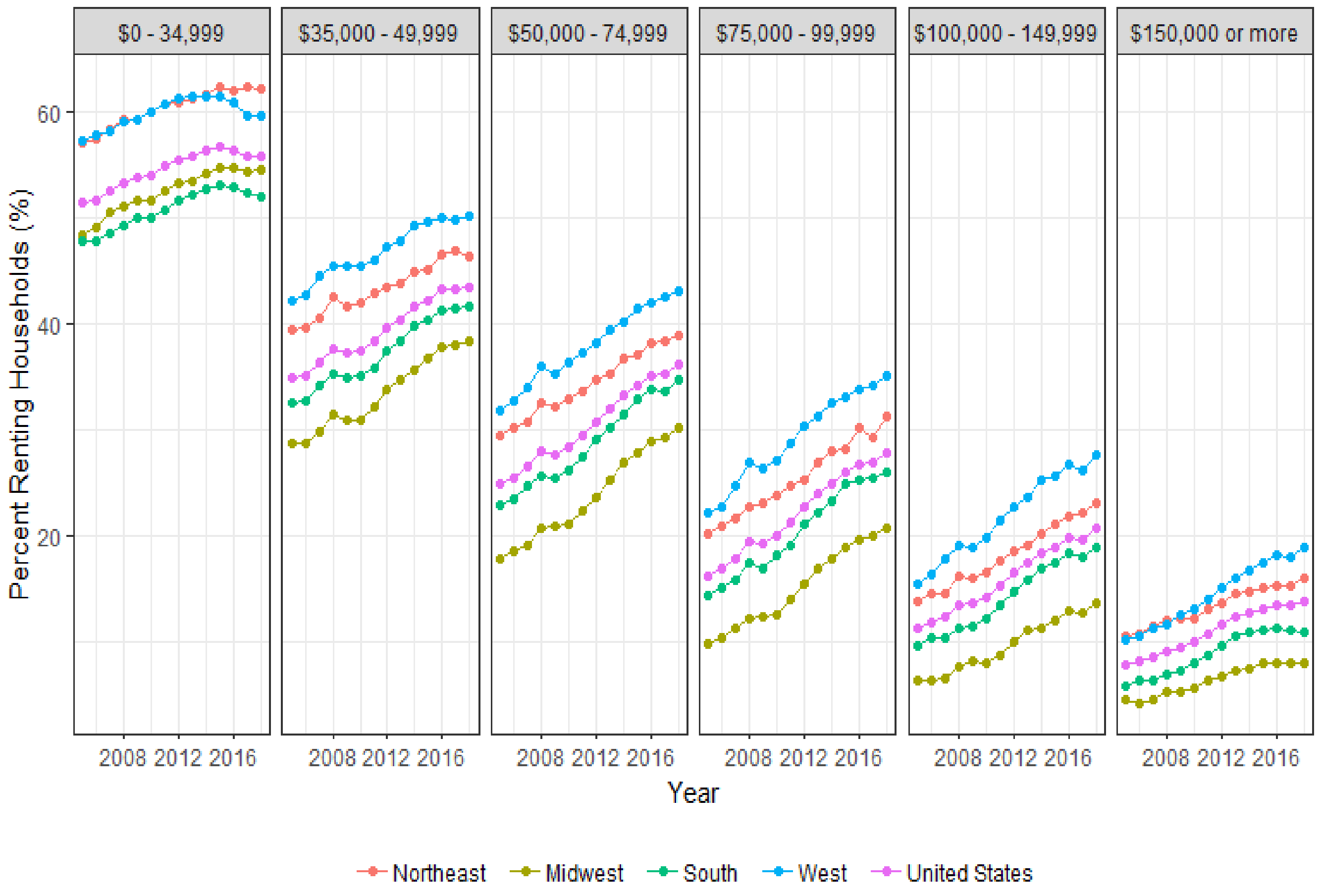

Fig. S7. Percent renting households by household income and region, 2000-2018
Source: 1-year American Community Survey, 2005-2018 (Table B25118)

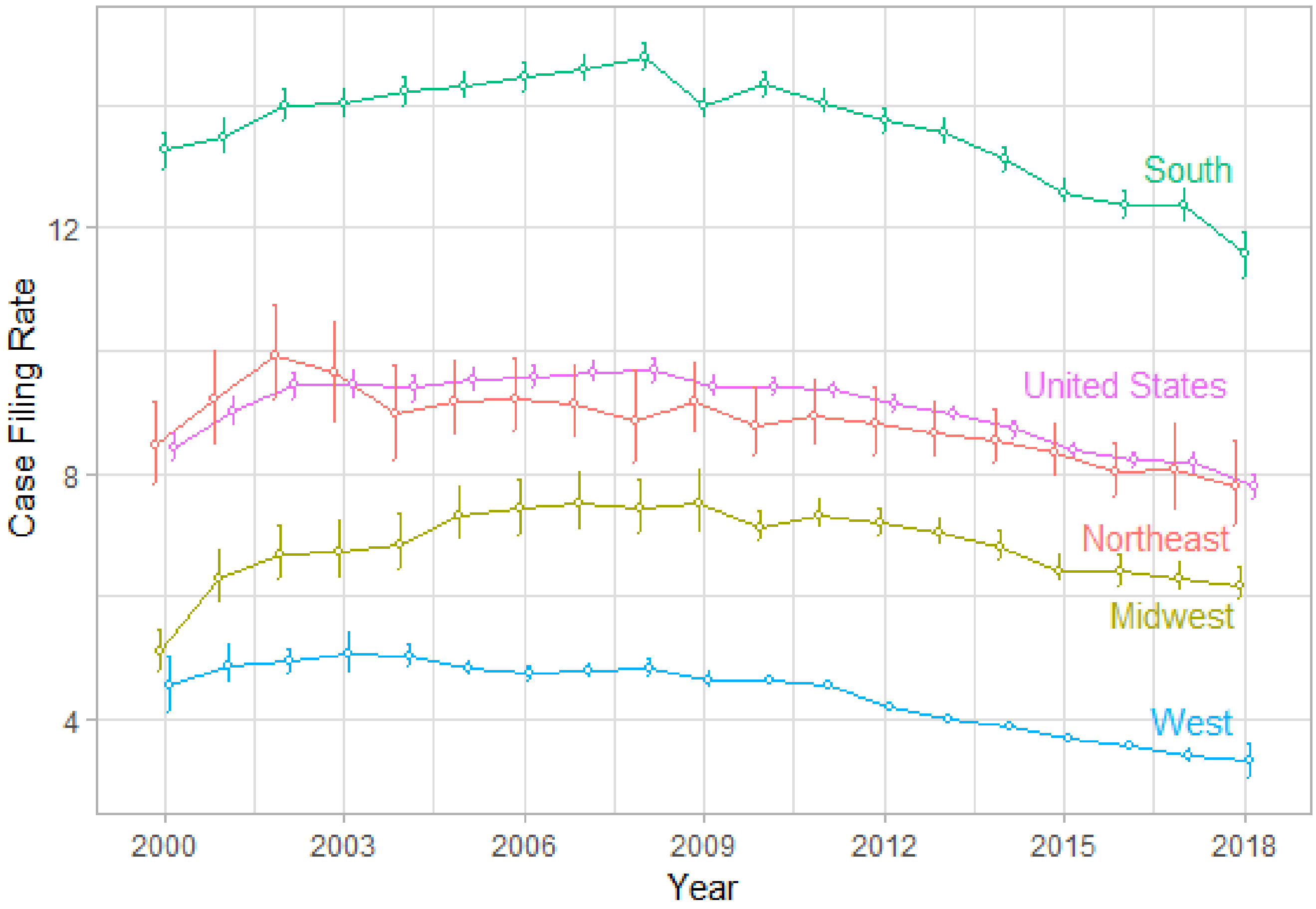

Fig. S8. Eviction filing rates, by region, 2000-2018.
Error bars show 95% credible interval.

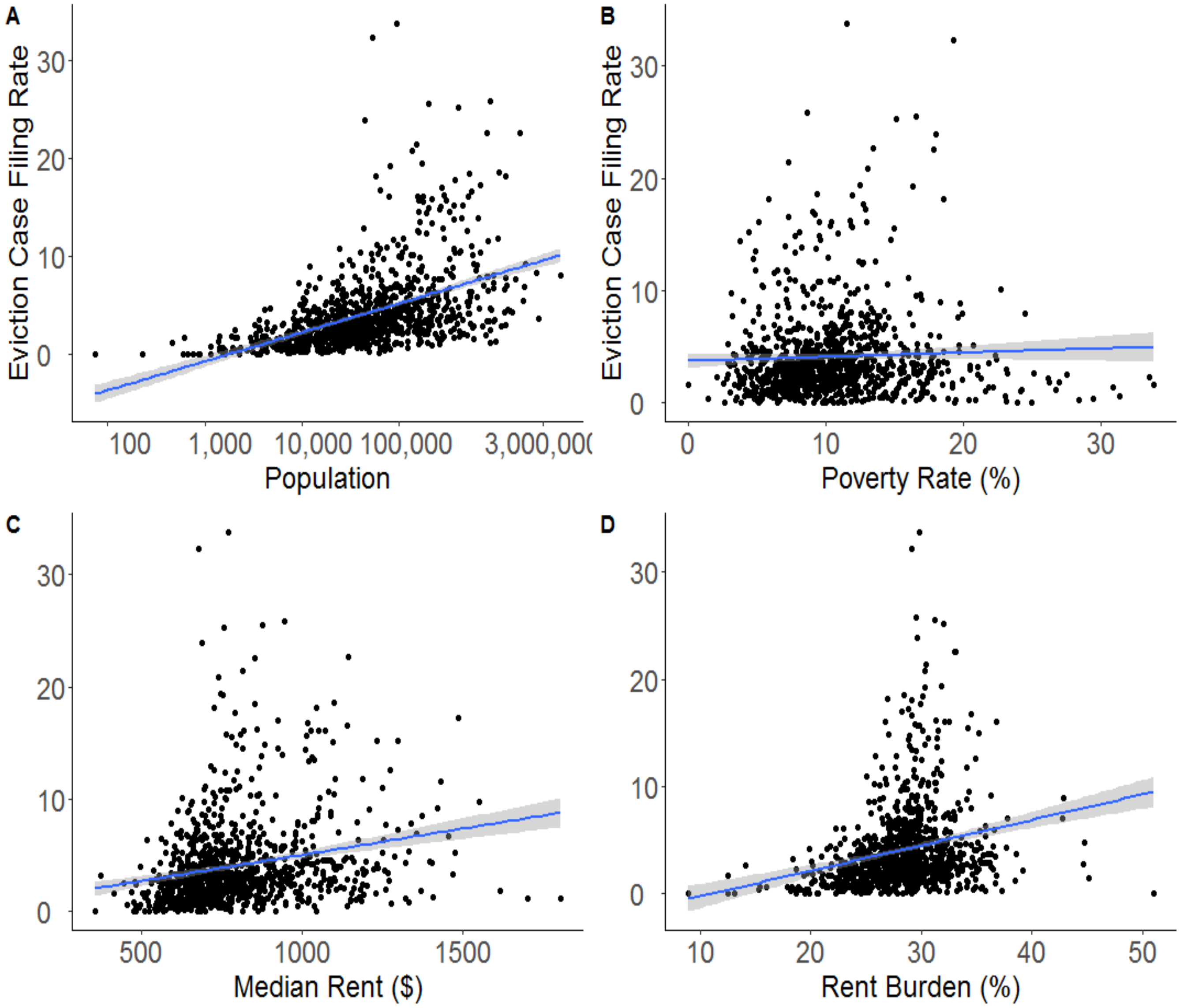

Fig. S9. Associations of county-level eviction filing rates with (A) population, (B) median rent, (C) poverty rate, and (D) rent burden, 2018

Blue lines show linear, bivarate association; shaded gray areas show 95% confidence intervals. N=980 counties with court-issued filing counts and complete ACS data on population, median rent, poverty rate, and rent burden. We did not include counties with estimated filing counts from the Bayesian posterior distribution as the model included median rent and number of renting households, which would be expected to be strongly correlated with population.

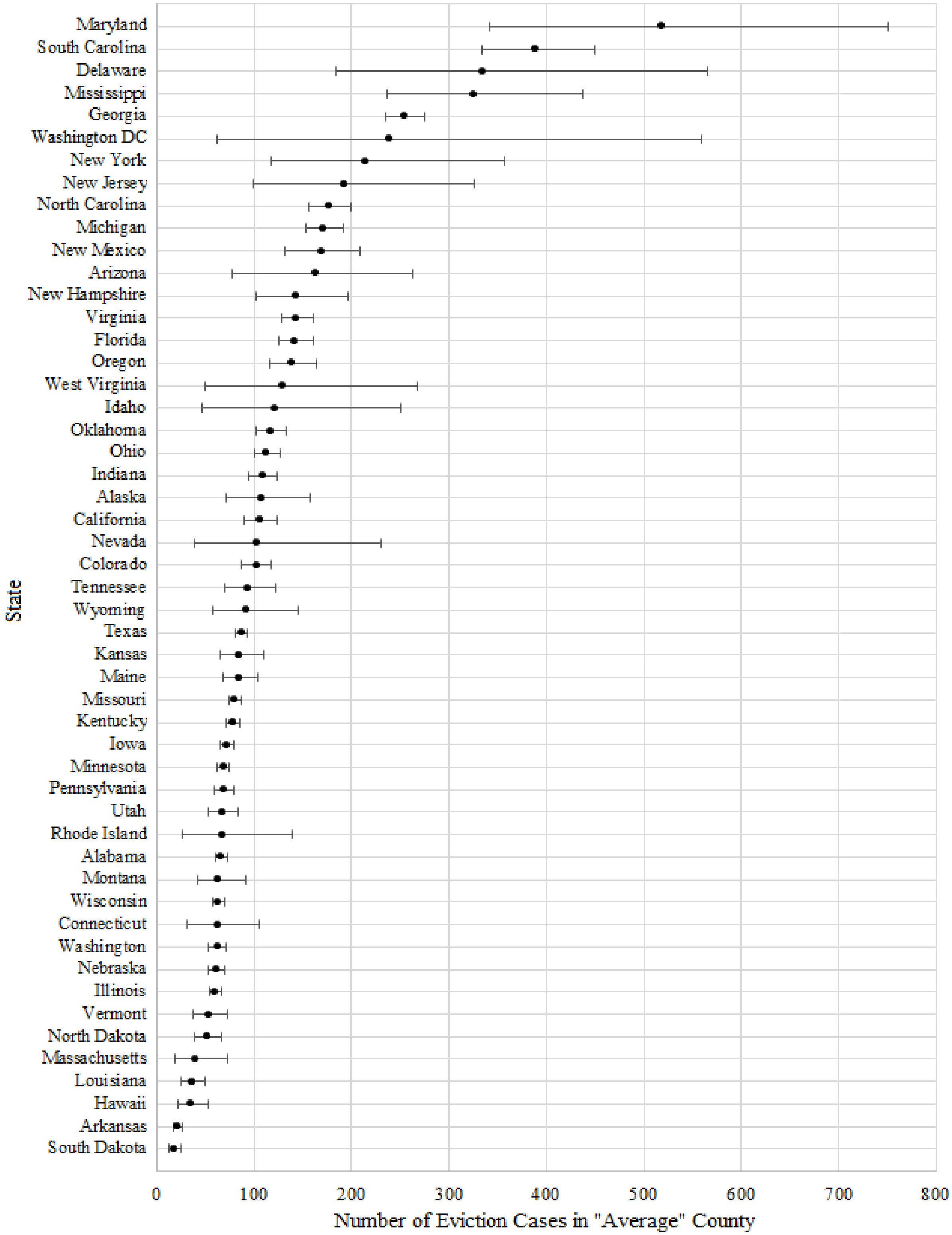

Fig. S10. Estimated eviction filings in demographically identical counties, by state, 2018

All covariates (number renting households, household density, percent African American population, median income, median rent, unemployment rate, and number of courts that hear eviction cases) are set equal to overall means and county and regional variation are marginalized over. Error bars show 95% credible intervals.

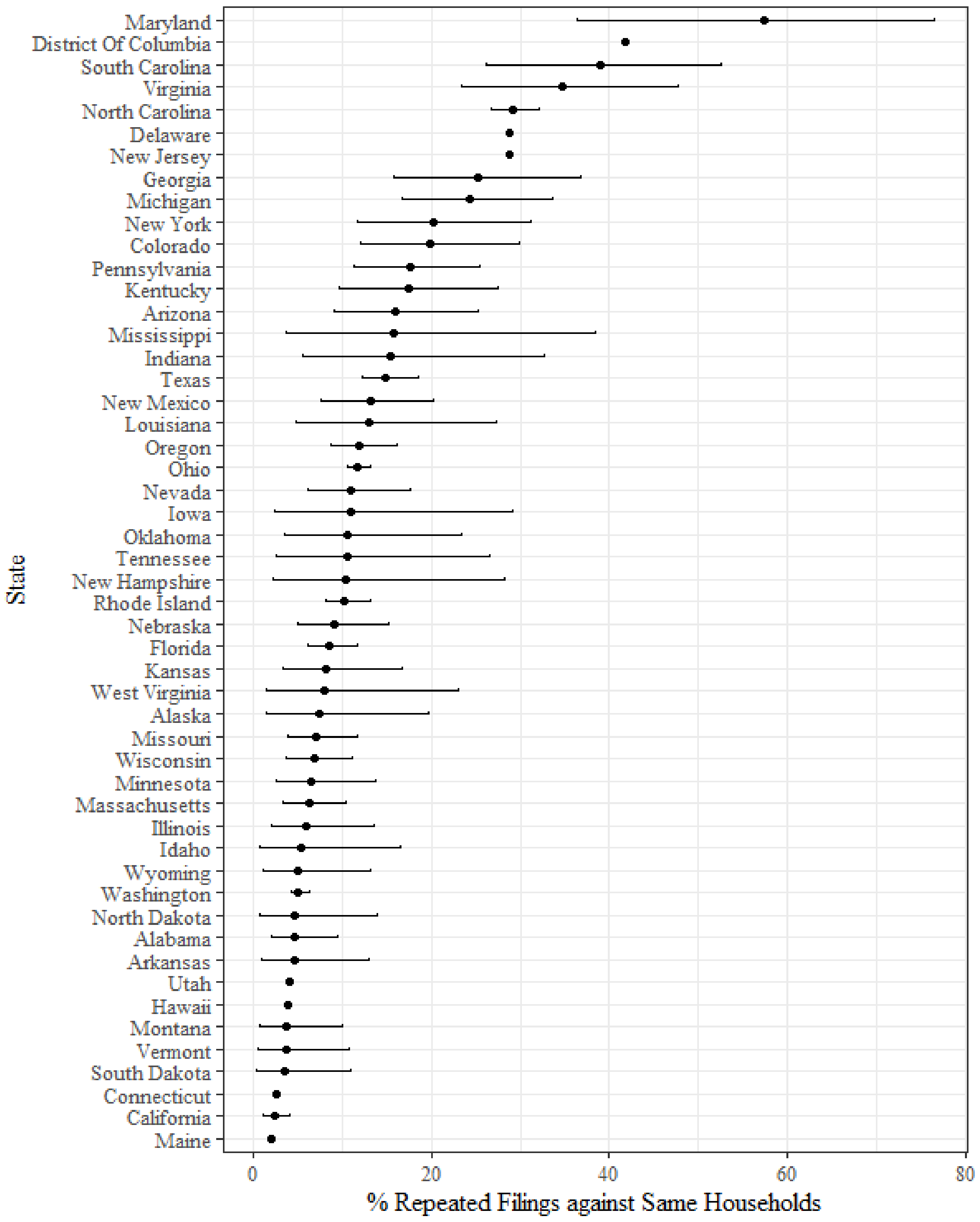

Fig. S11. Percent repeated filings against same households, by state, 2018

Percent repeated filings calculated by dividing number of unique households represented in filings by total number of filings. Error bars show 95% credible intervals.

(A)

(B)

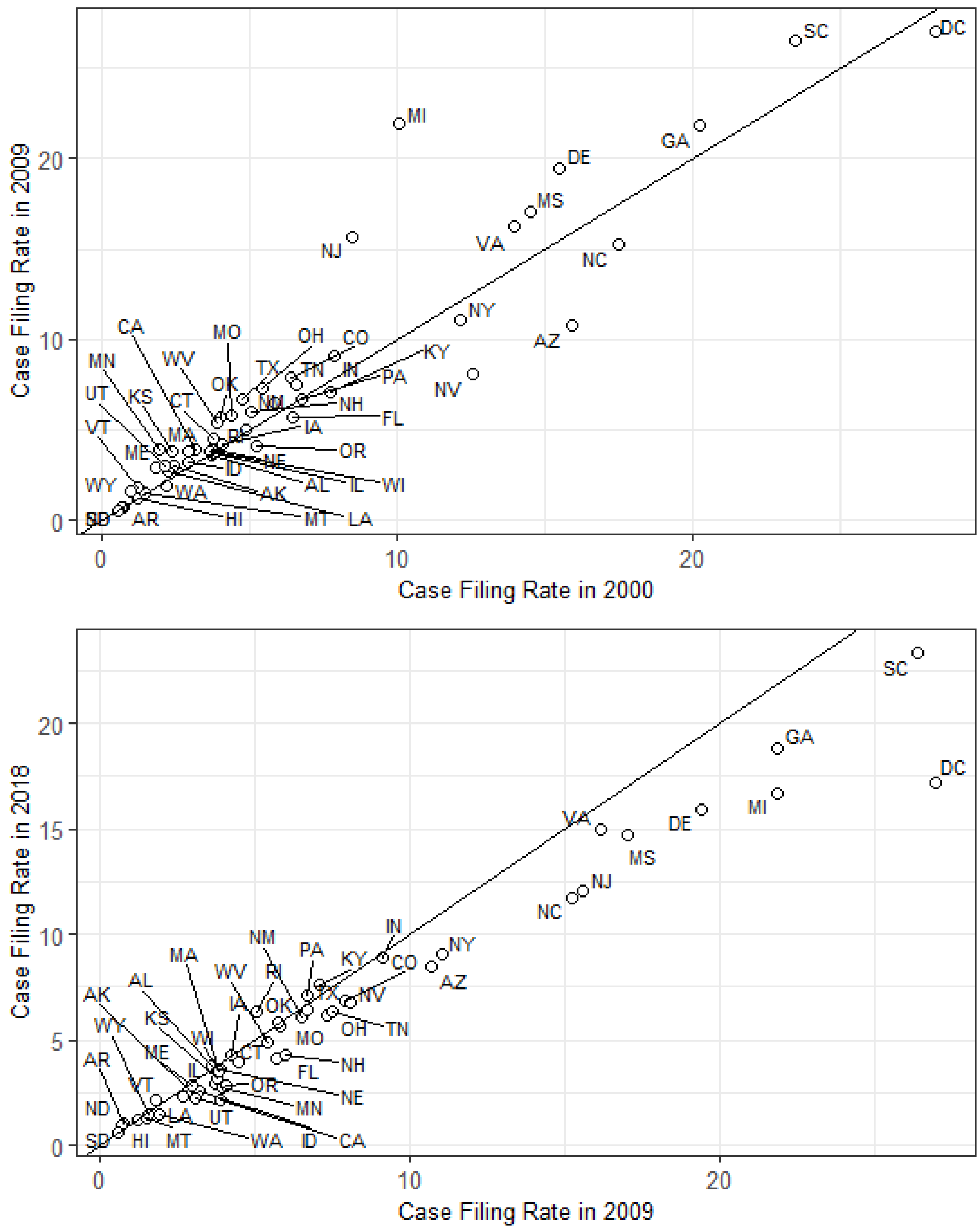

Fig. S12. State-level eviction filing rates, 2000 to 2009 (A) and 2009 to 2018 (B)

Filing rates for each state are plotted against one another for the years indicated on the axes. Filing rates calculated as predicted number of filings divided by number of renting households. The black line marks the diagonal. States falling close to the diagonal have roughly the same case rate in both years. Although some states shift above or below the diagonal, the relative disparities between states do not shift substantially.

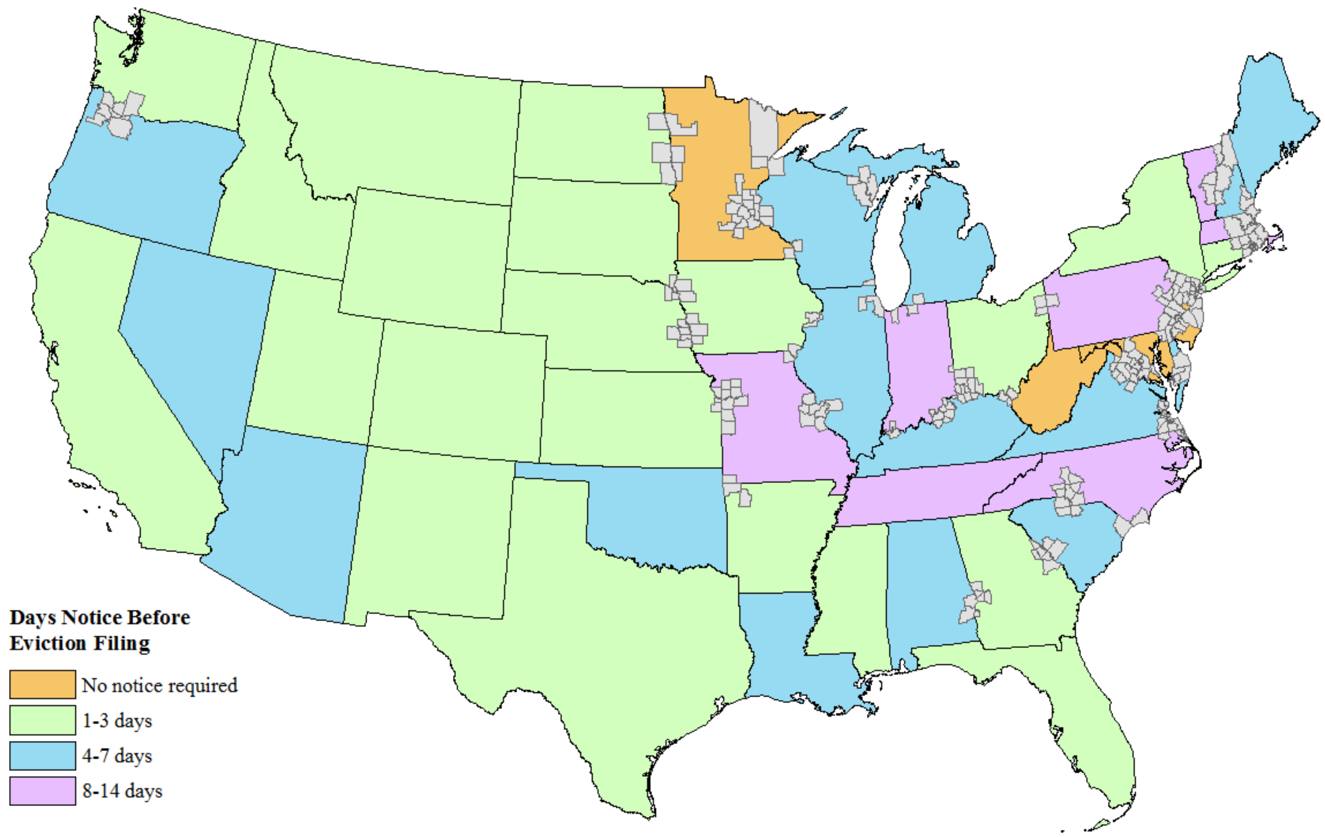

Fig. S13. Counties included in eviction notice requirements analyses

The primary analytic sample included 230 counties (shown in gray) located in 39 CBSAs that crossed state borders (N=3,074 county years). CBSAs included in sample listed in Table S8.

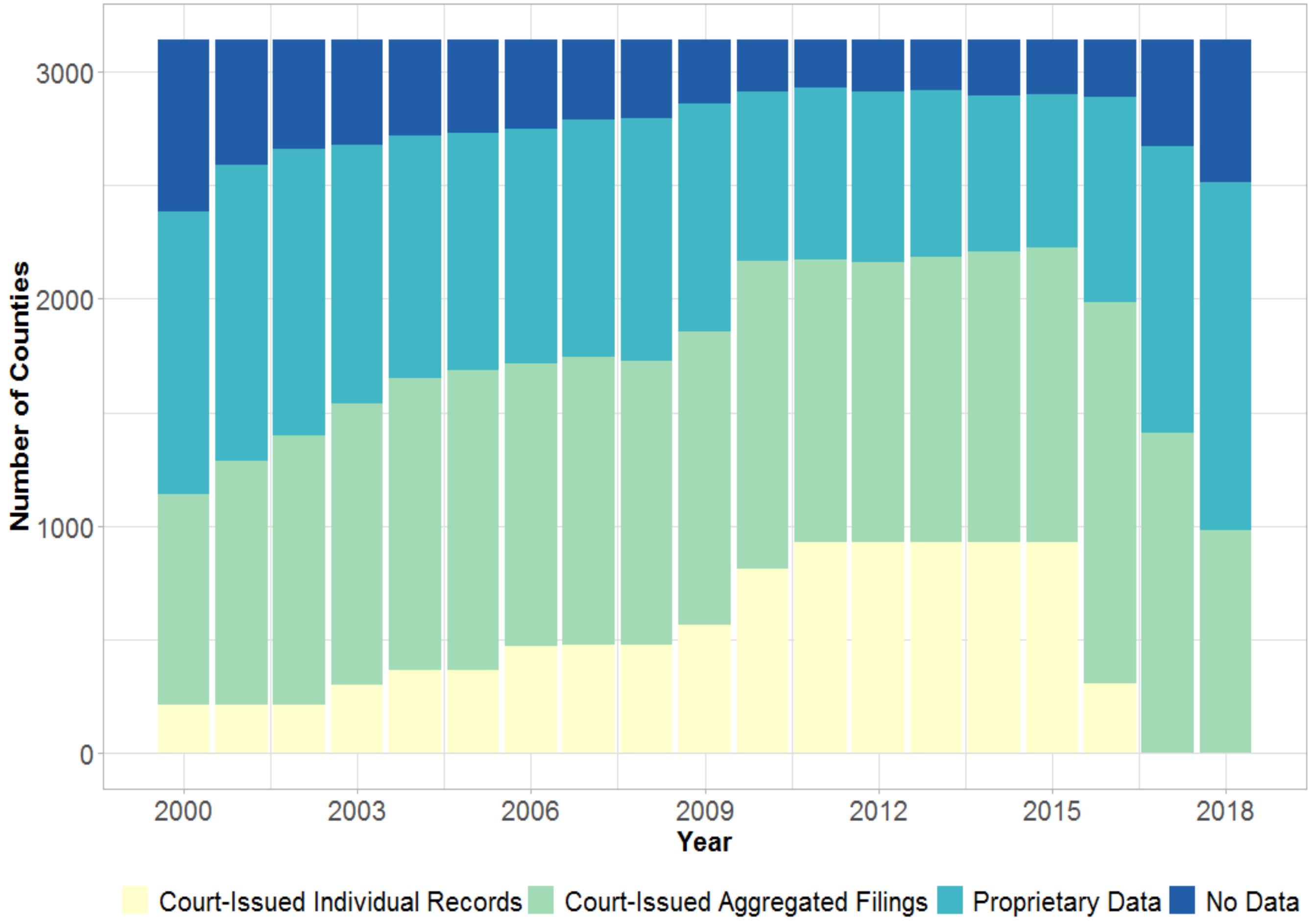

Fig. S14. Data sources underlying eviction filing estimates, 2000-2018
N=3,143 counties in each year (59,717 total county-years).

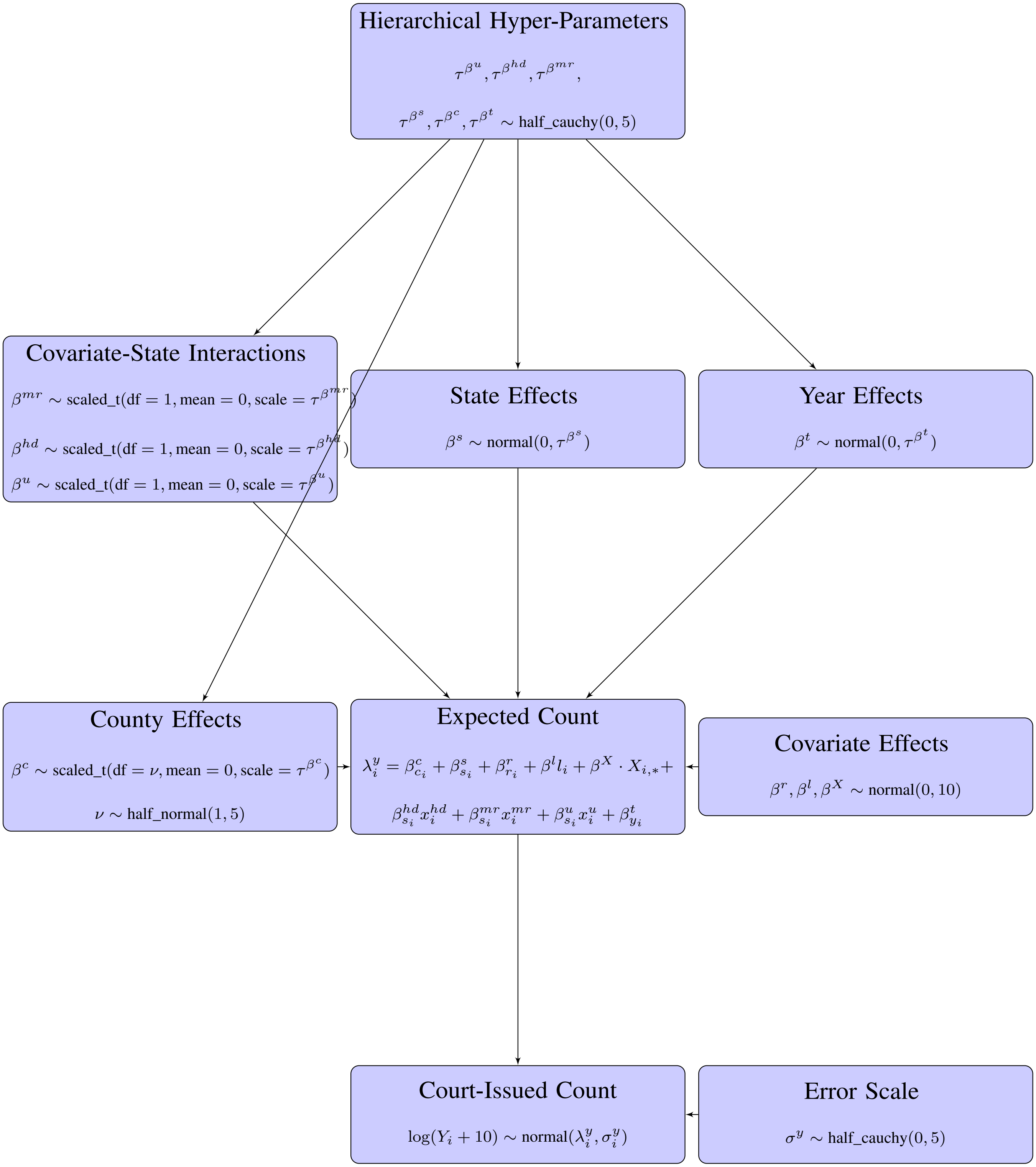

Fig. S15. Graph of probability model for county-level filing counts in court-issued data (Y)

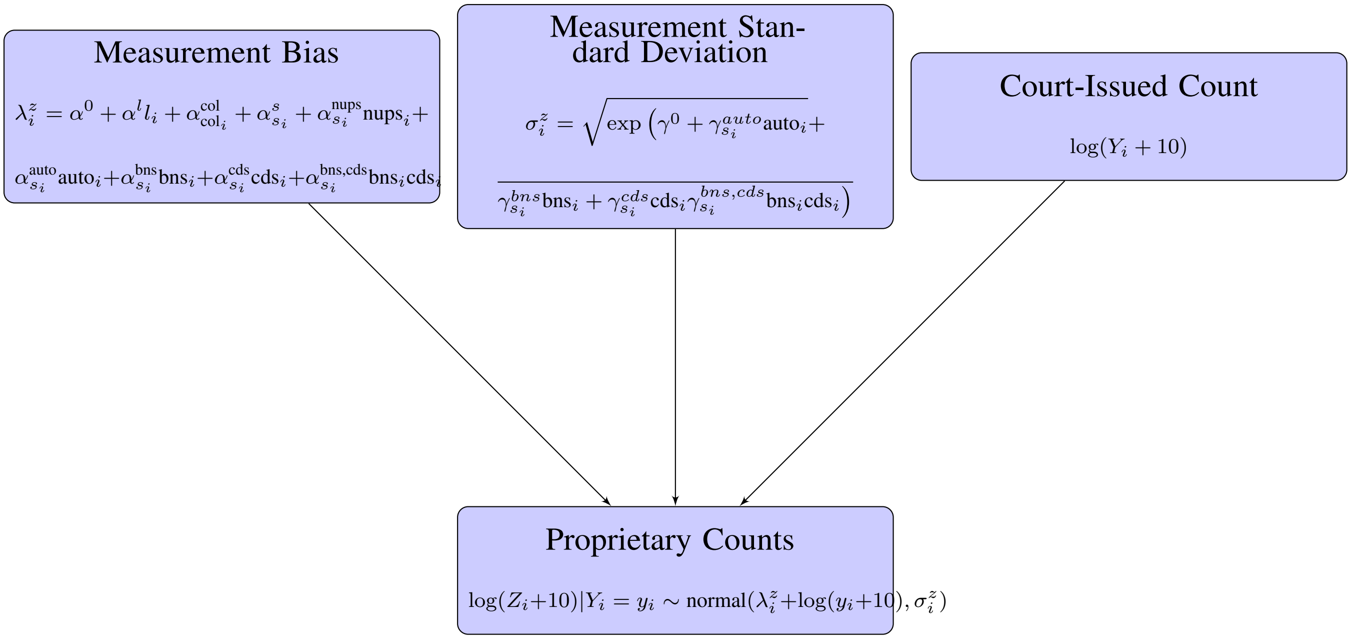

Fig. S16. Graph of measurement error model for Z

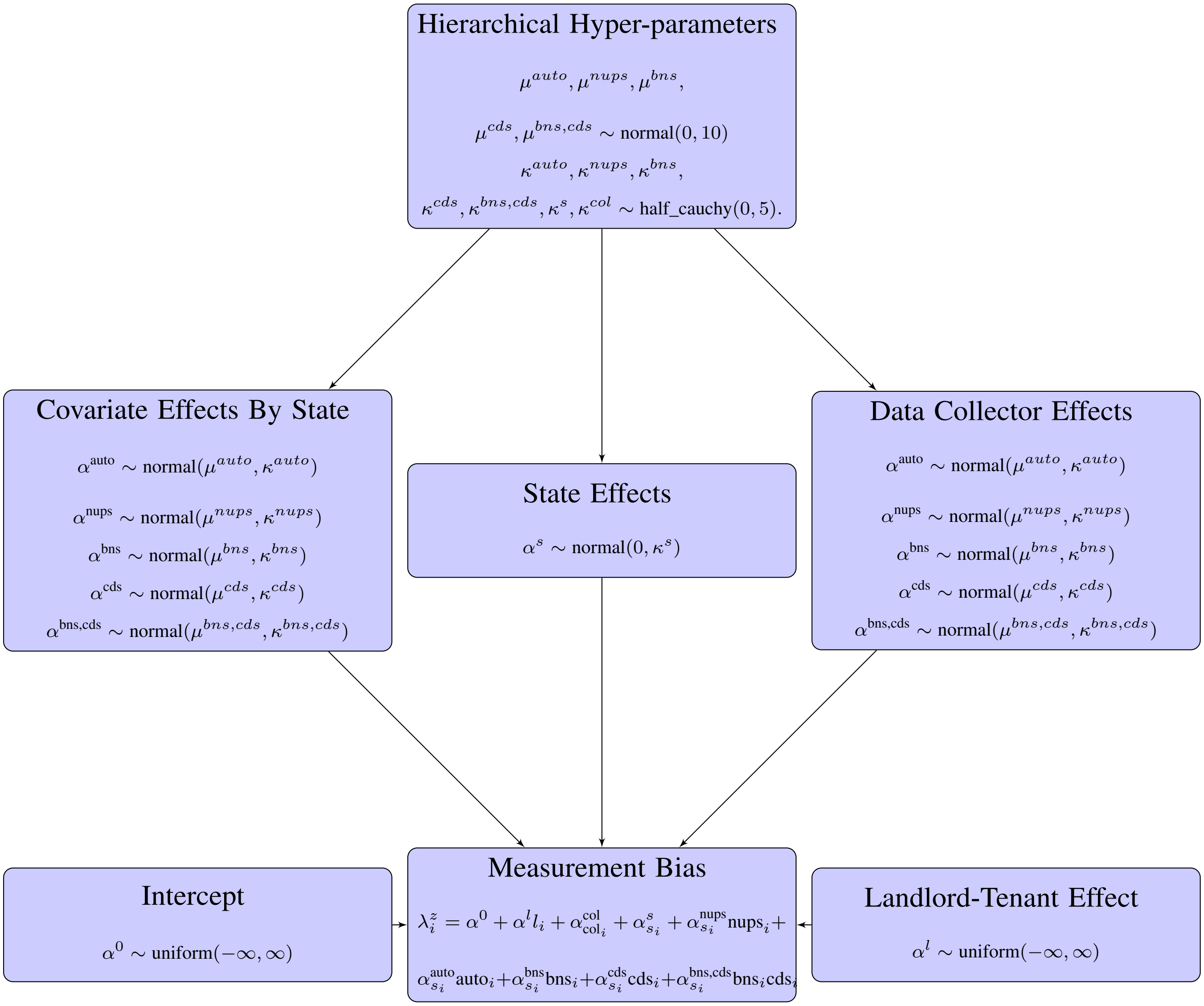

Fig. S17. Graph of measurement bias component of measurement error model for Z

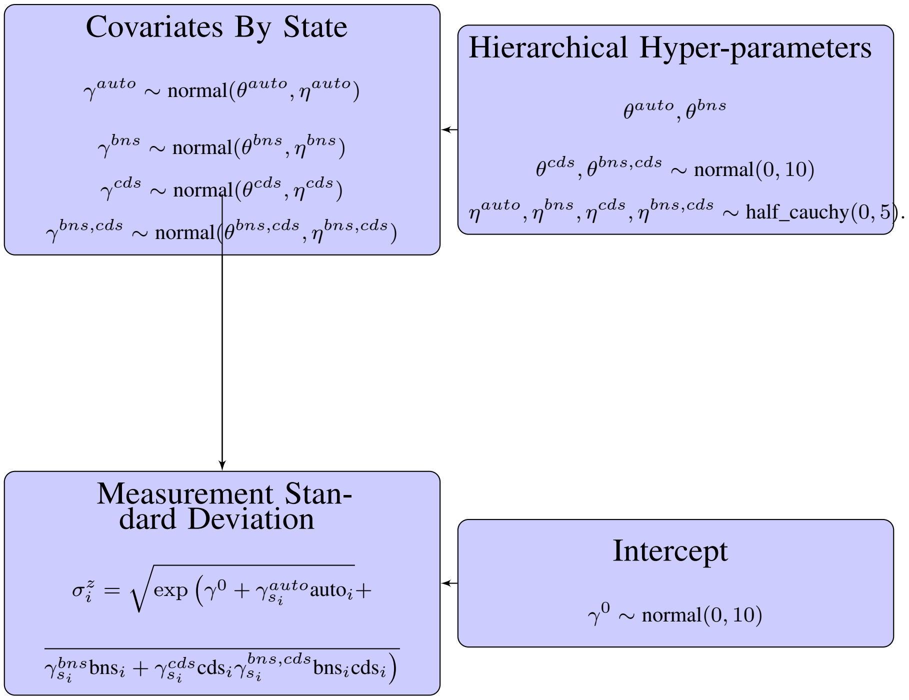

Fig. S18. Graph of the variance portion of the measurement error model for Z

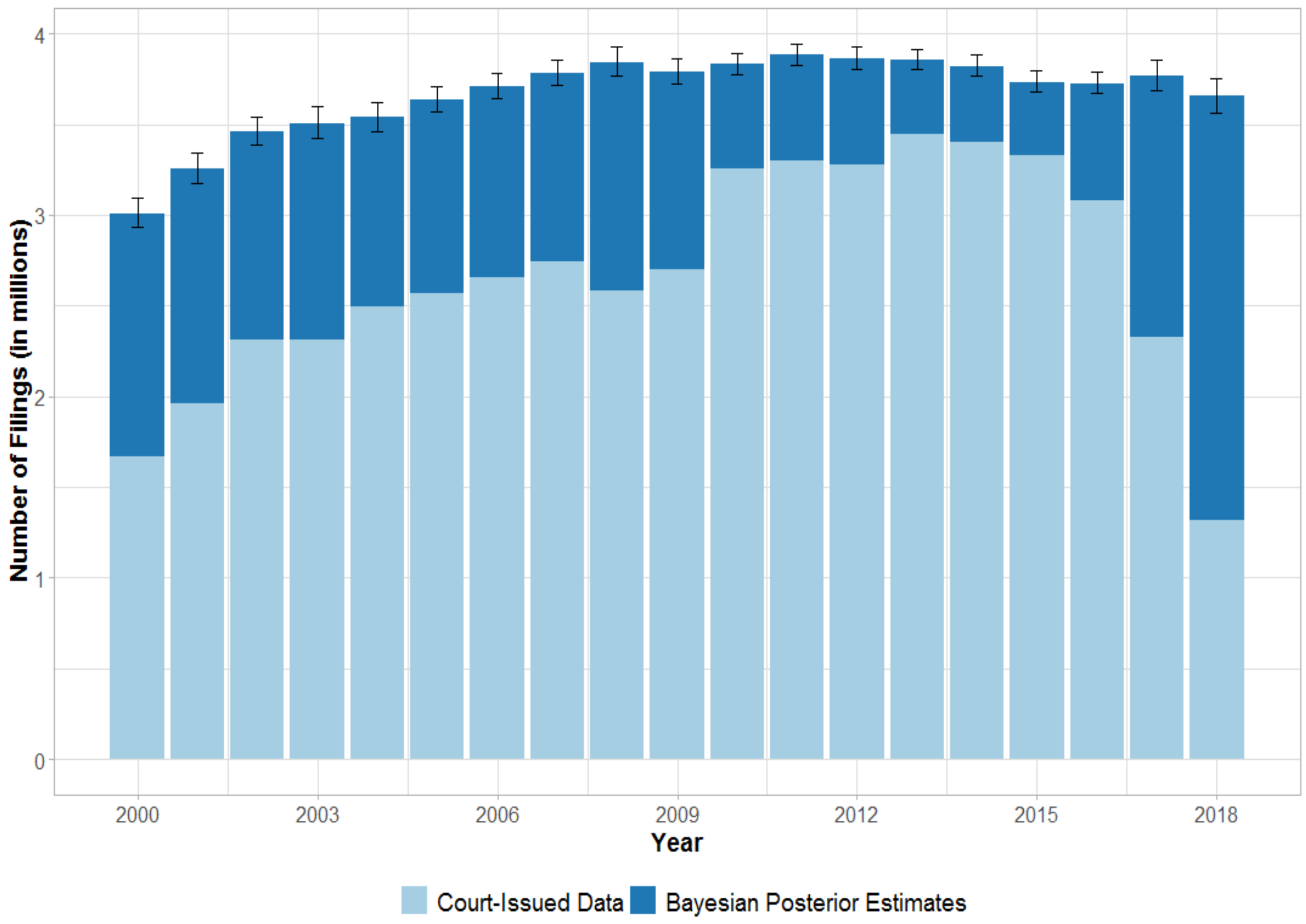

Fig. S19. National eviction filing estimates, by data source, 2000-2018

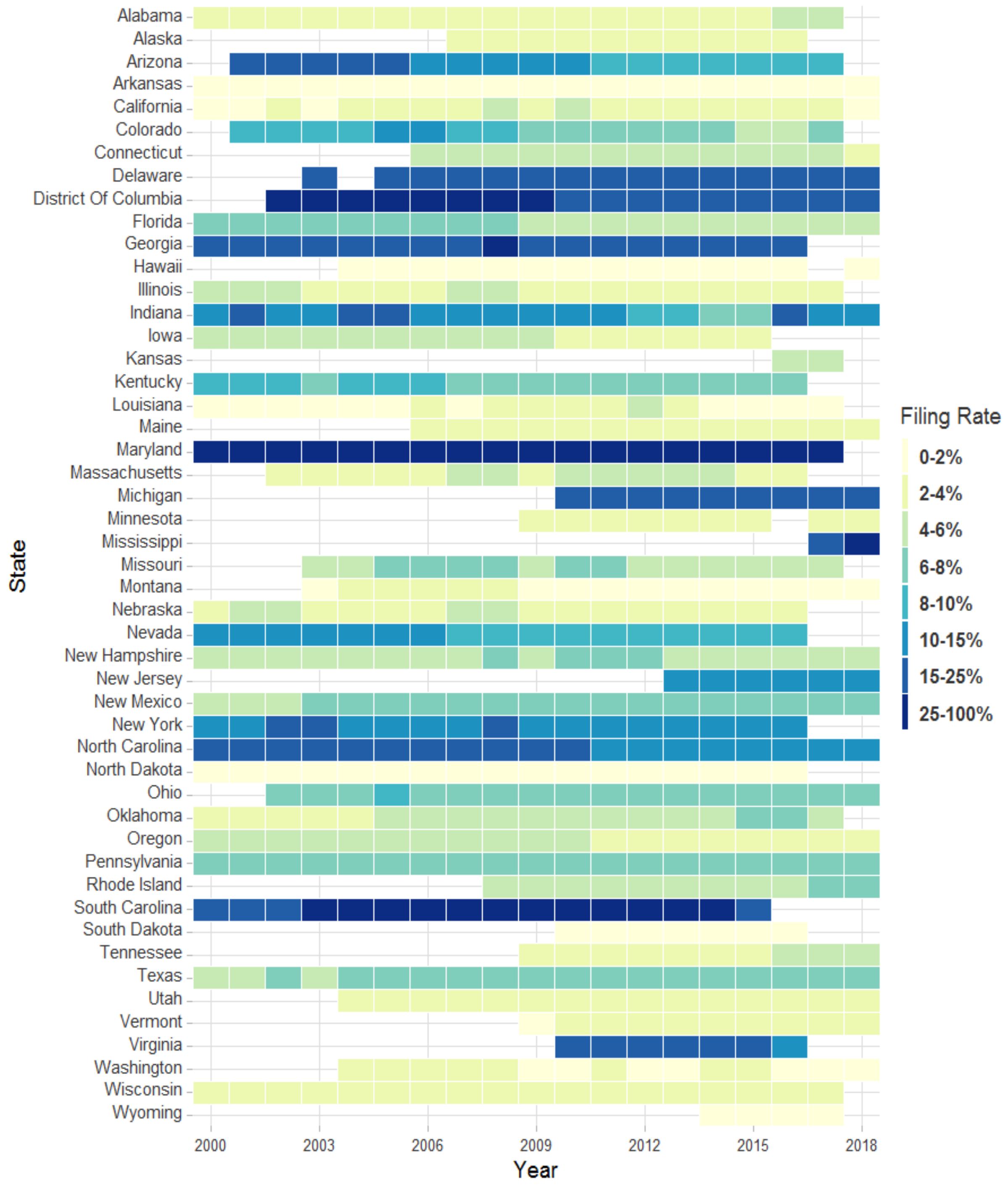

Fig. S20. Longitudinal eviction filing rates from court-issued data, by state, 2000-2018

Note: There was some fluctuation in availability of court-issued data across counties within states over time. Caution should be used when making direct year-to-year comparisons within states.

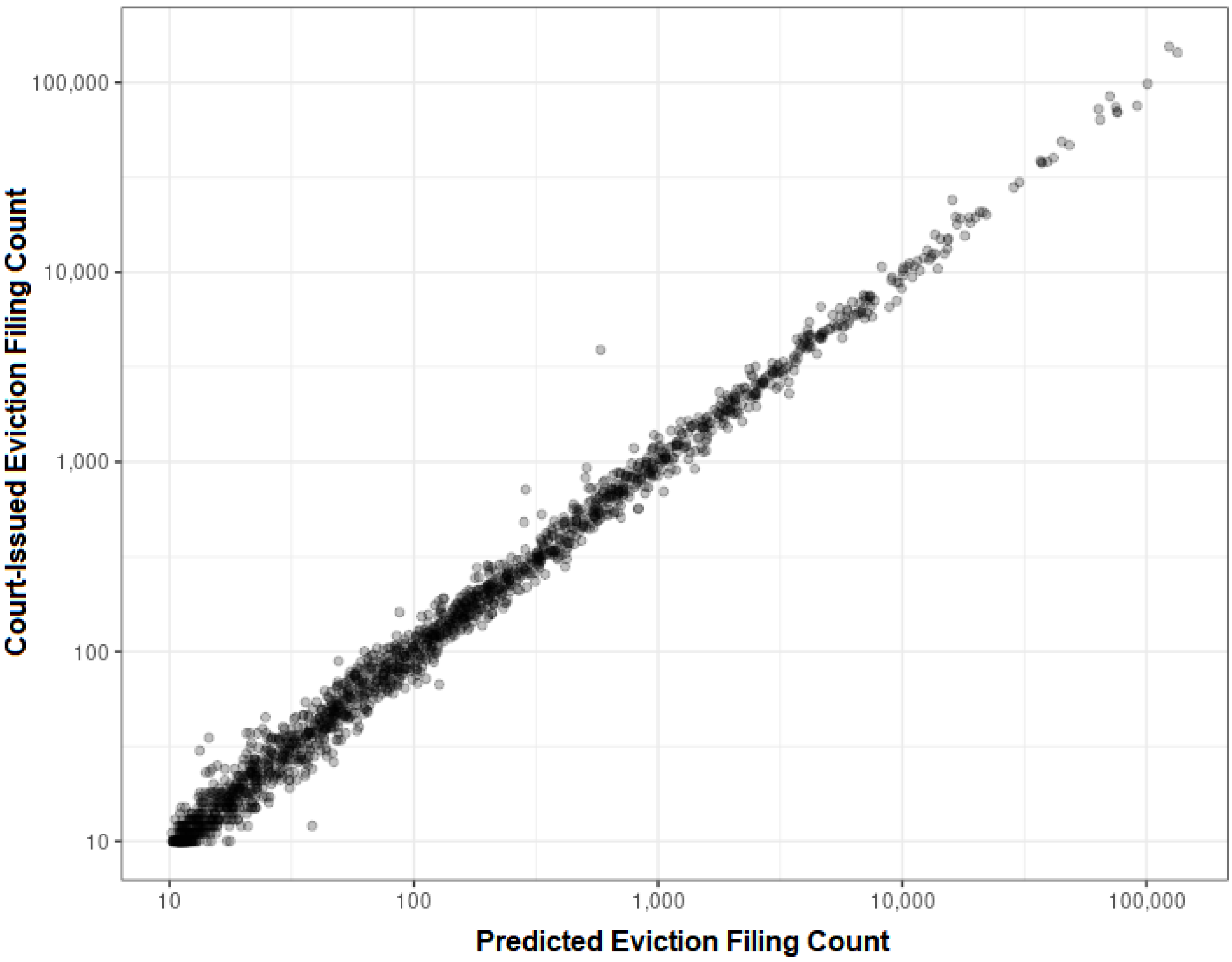

Fig. S21. Court-issued filing counts compared with Bayesian posterior mean predicted values with randomly selected holdout set (5%) of county-years

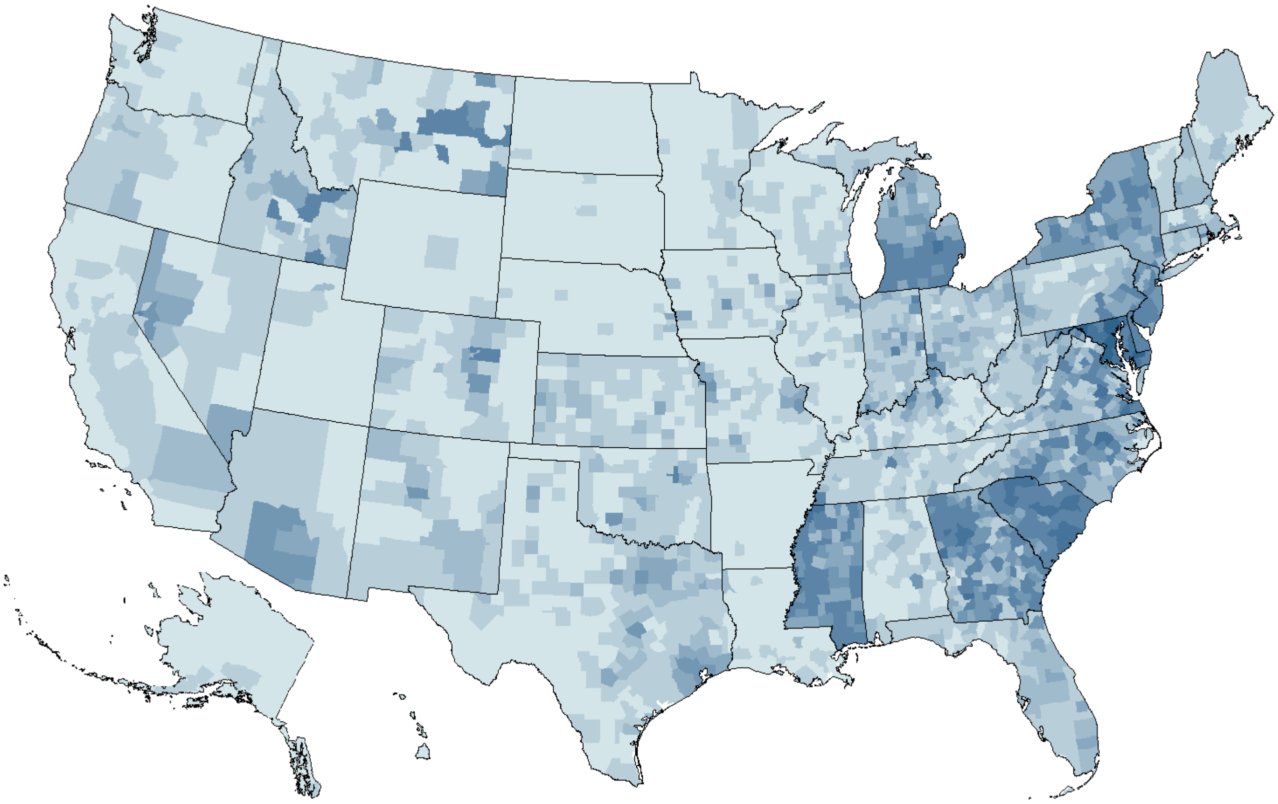

Fig. S22. County-level eviction filing rates predicted in Bayesian posterior mean distribution, 2018

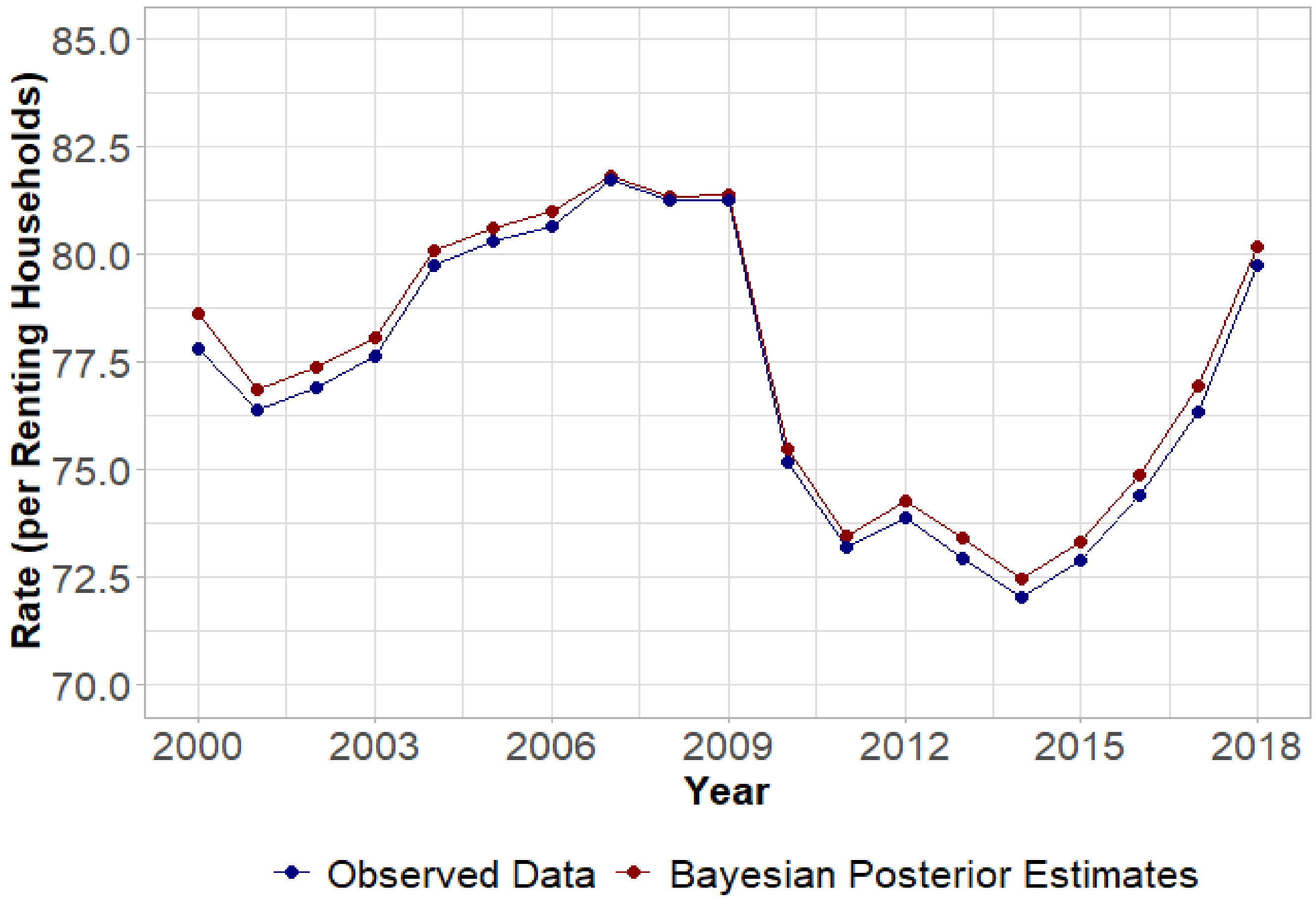

Fig. S23. Estimated percent eviction filings representing unique households, by observed data and Bayesian posterior predictions, 2000-2018

N=12,870.

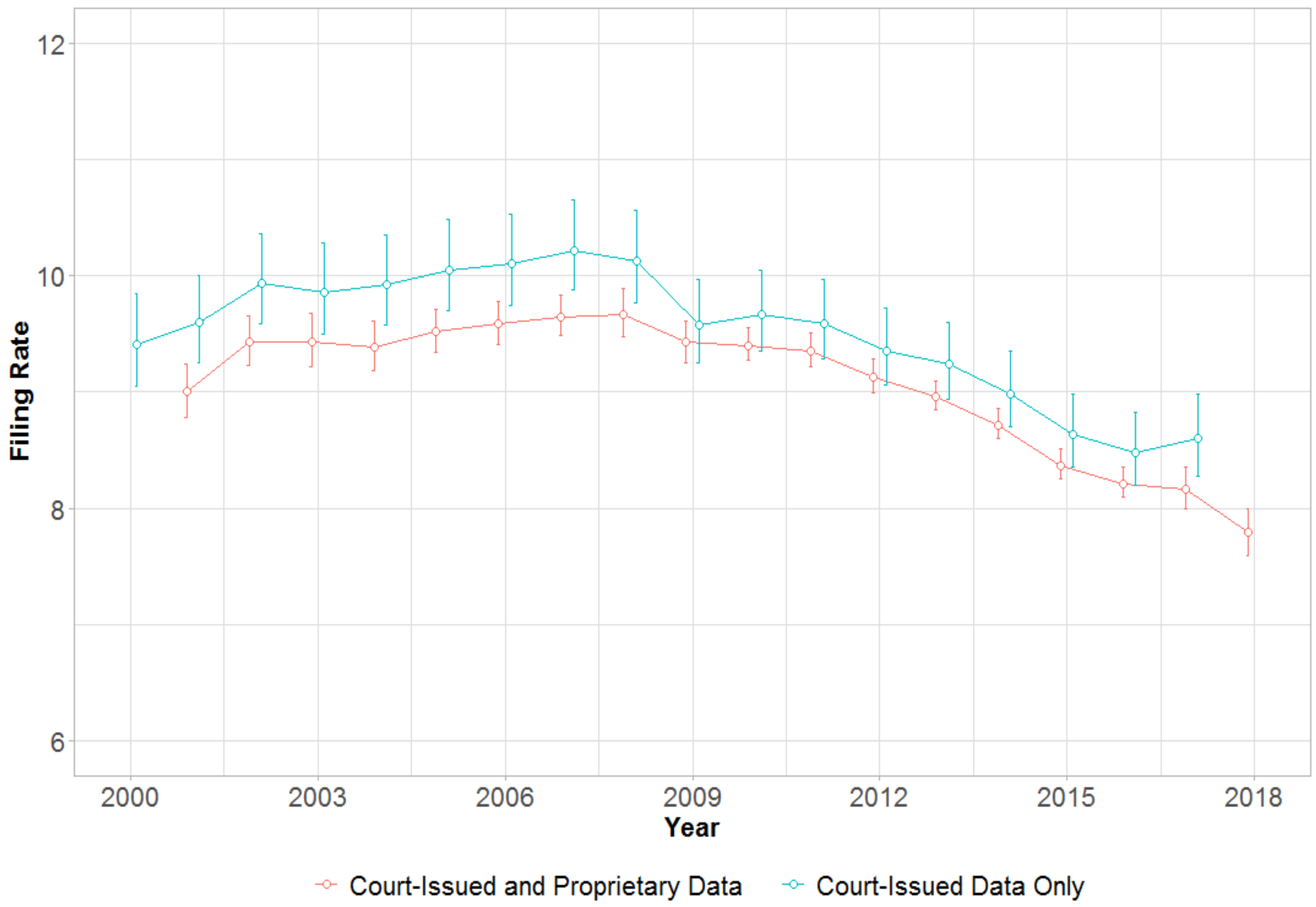

Fig. S24. National eviction filing rate, by inclusion of proprietary data as secondary measure of court-issued filing counts in Bayesian model, 2000-2018

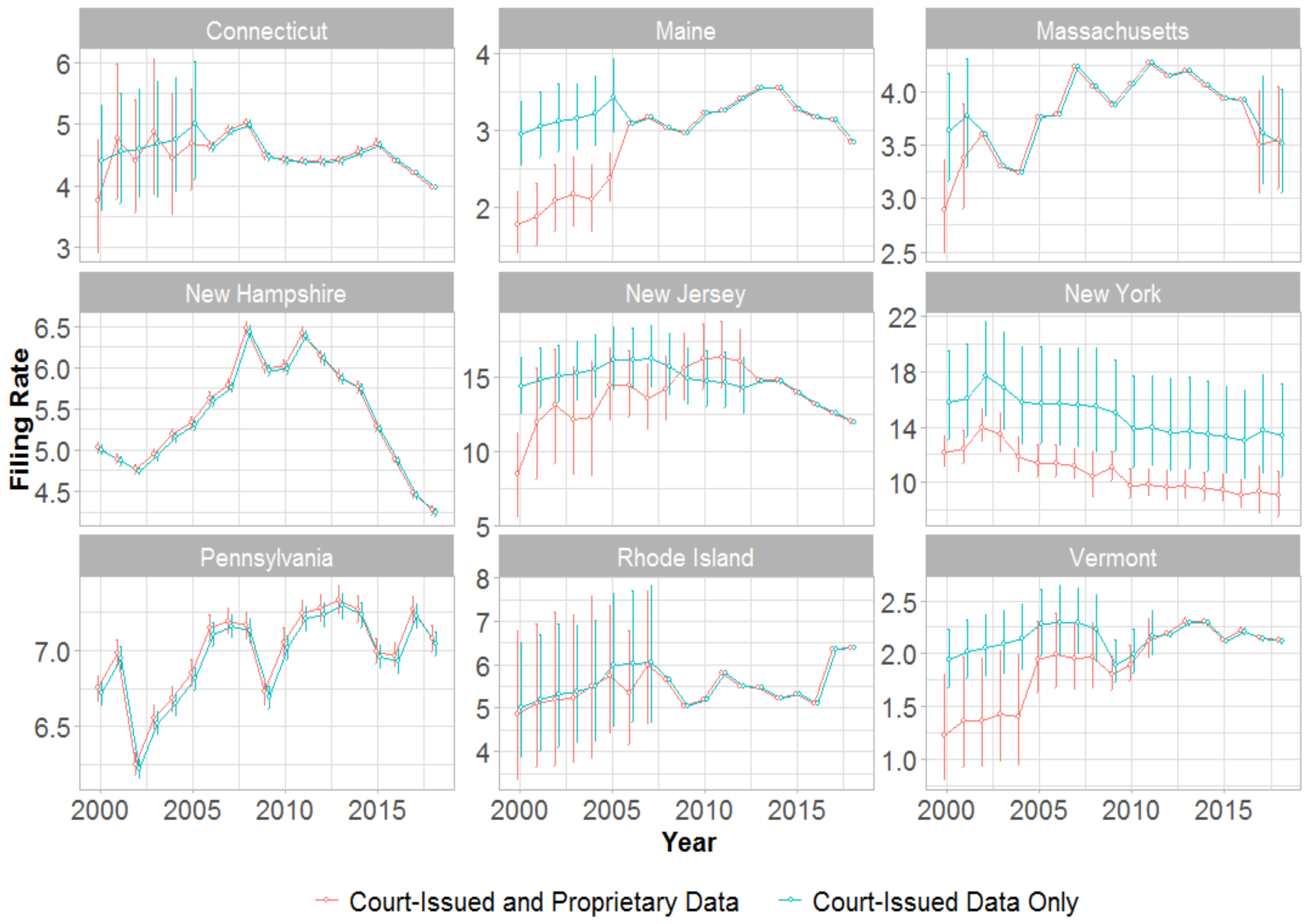

Fig. S25. State eviction filing rates, by inclusion of proprietary data as secondary measure of court-issued filing counts in Bayesian model, Northeast, 2000-2018

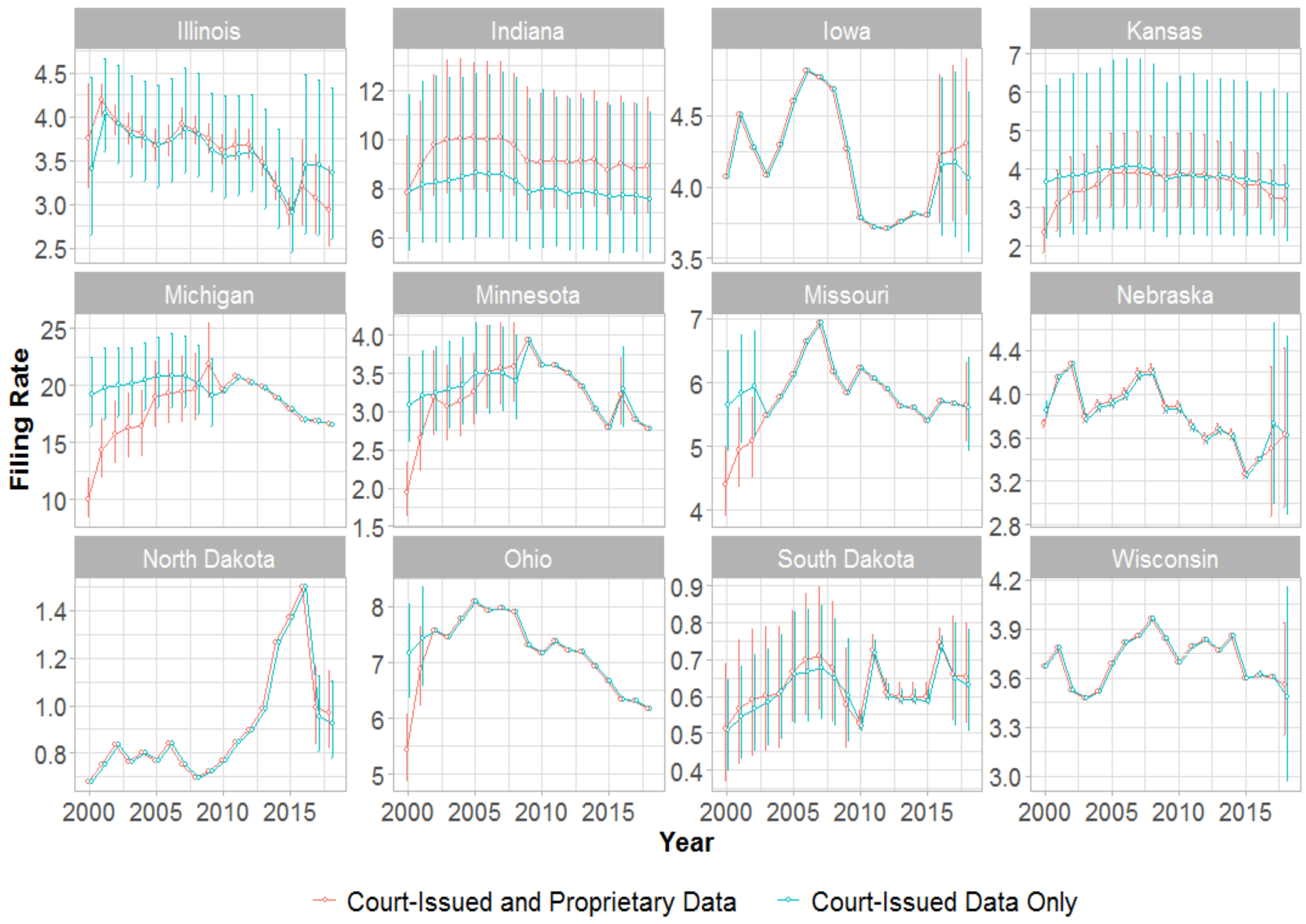

Fig. S26. State eviction filing rates, by inclusion of proprietary data as secondary measure of court-issued filing counts in Bayesian model, Midwest, 2000-2018

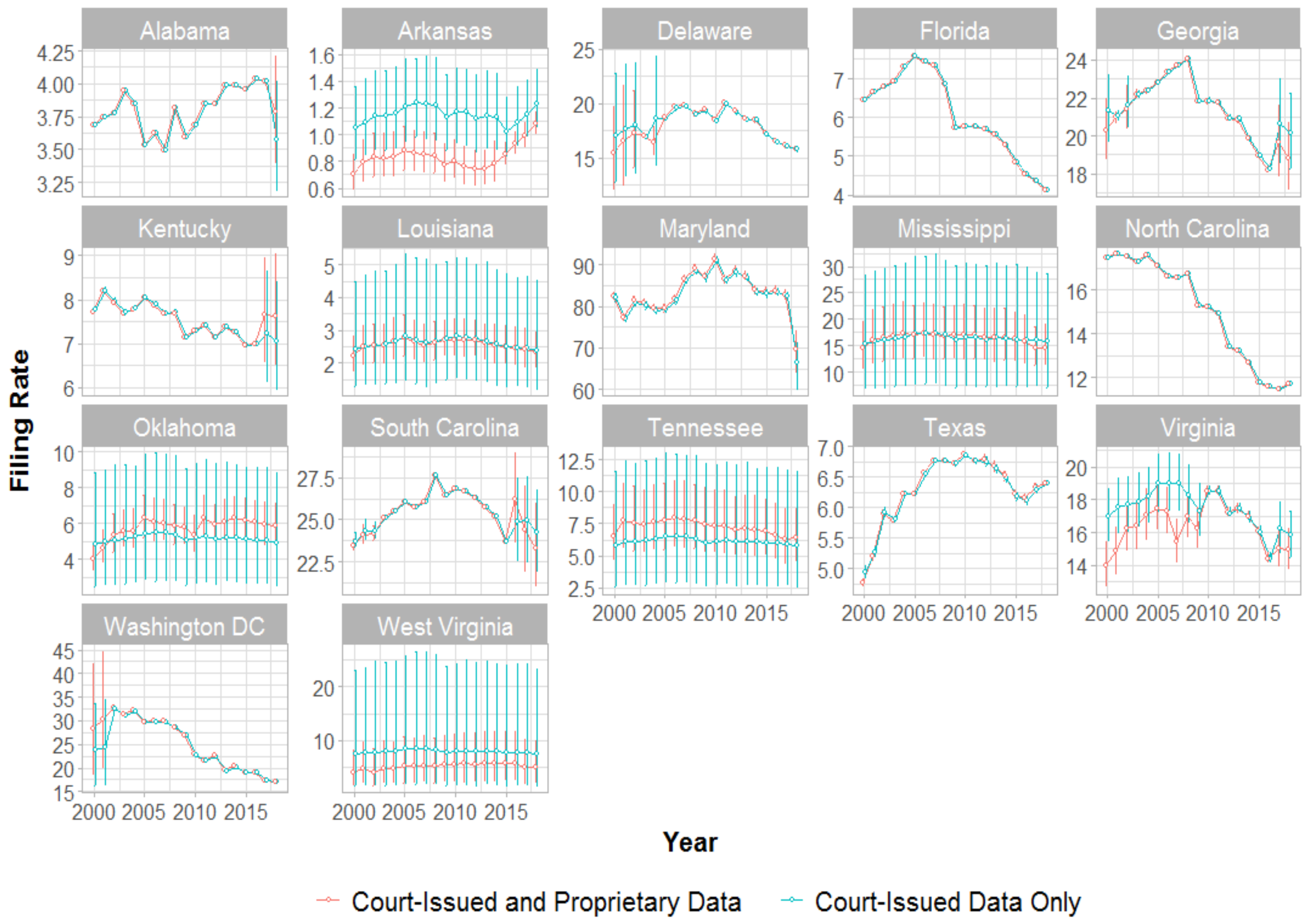

Fig. S27. State eviction filing rates, by inclusion of proprietary data as secondary measure of court-issued filing counts in Bayesian model, South, 2000-2018

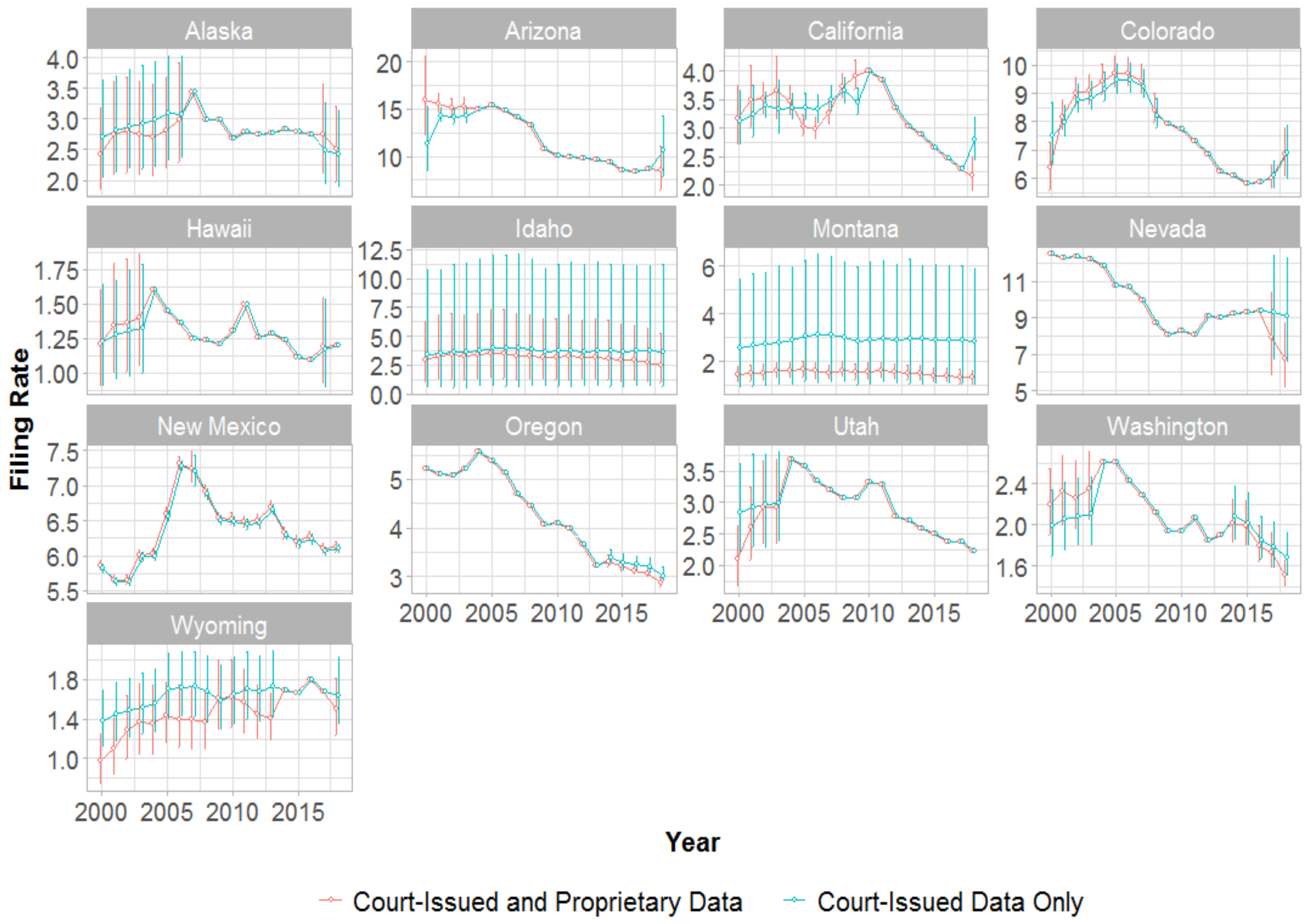

Fig. S28. State eviction filing rates, by inclusion of proprietary data as secondary measure of court-issued filing counts in Bayesian model, West, 2000-2018

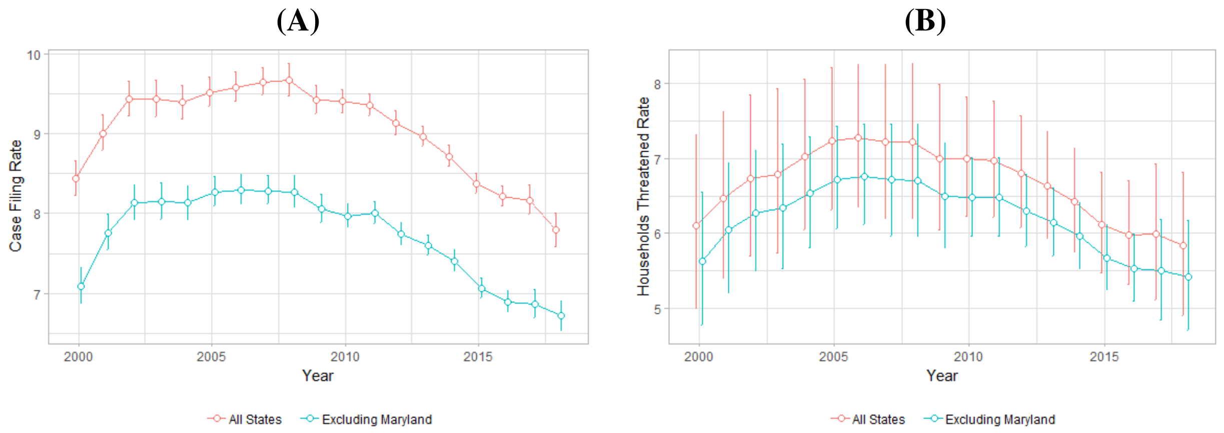

Fig. S29. Estimates of filing rates (A) and rates of households threatened with eviction (B), with and without Maryland, 2000-2018

Rates calculated by dividing the number of filings and unique households threatened with eviction by the number of renting households. Renting households estimated using linear interpolation between 2000 and 2010 Censuses and 2016 ESRI Business Analyst.

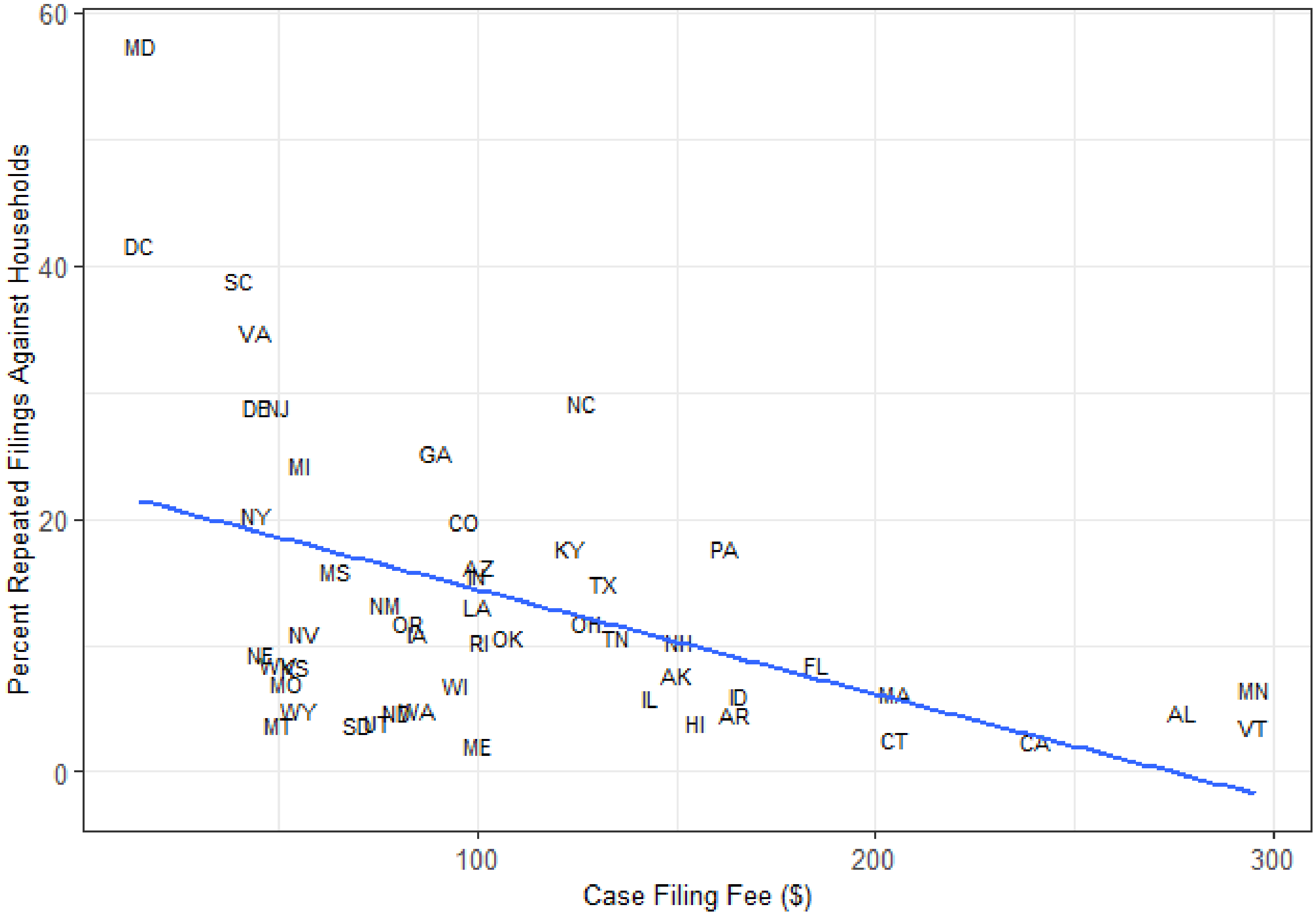

Fig. S30. Association between case filing fees and repeated filings against households, 2018
Rates of repeated filings against households were calculated by dividing the number of filings against households that had already experienced at least one prior case filing within the same calendar year by the total number of filings. Collection of case filing fees discussed in Section 1.3.13; filing fees were averaged across counties within states. Labels indicate state abbreviation. Solid blue line shows linear association. There is a negative relationship between filing fees and the percent of cases that represent repeat filings against the same household. Cost of filing is not a direct measure of the difficulty or ease of filing eviction cases across states; however, lower fees may reflect other aspects of the filing process that create lower barriers to entry for landlords using the court system. For example, very low filing fees in Maryland likely reflect the use of case filings as the initial eviction notice to tenants, resulting in substantially higher landlord filing volume.

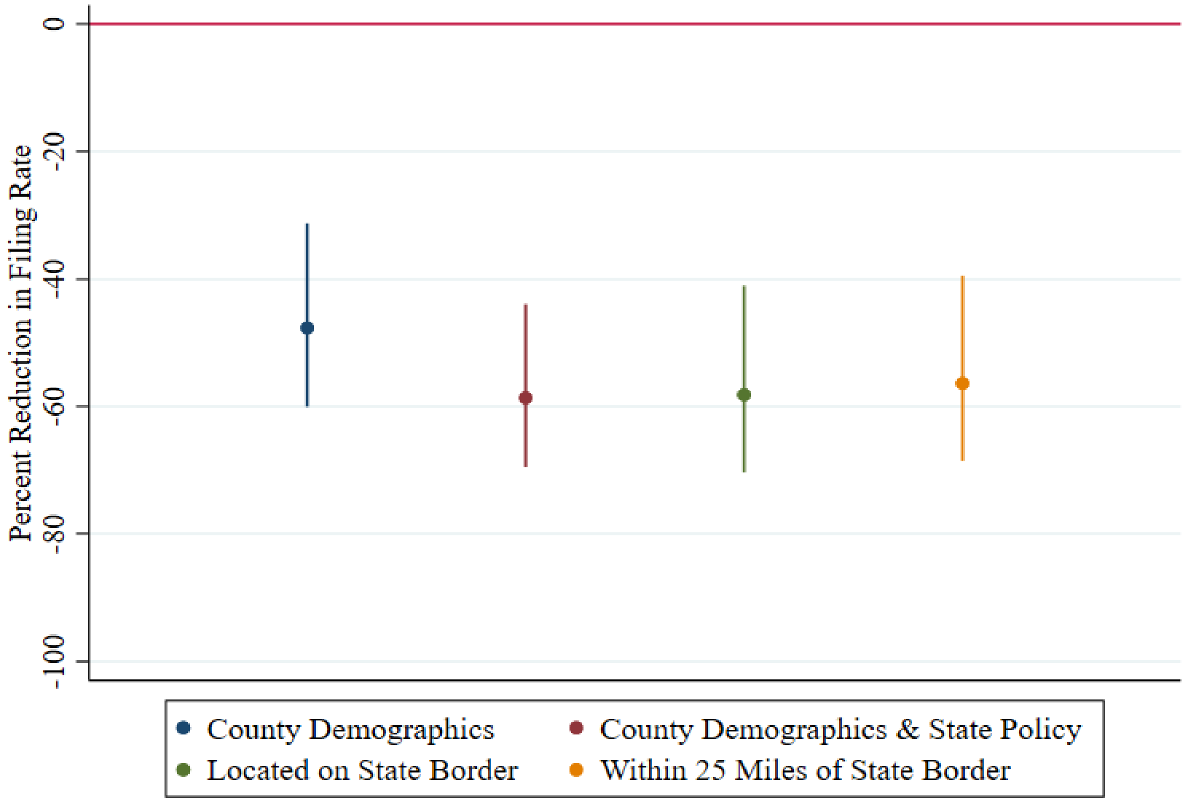

Fig. S31. Association between notice requirements for nonpayment eviction filings and eviction filing rate (binary indicator)

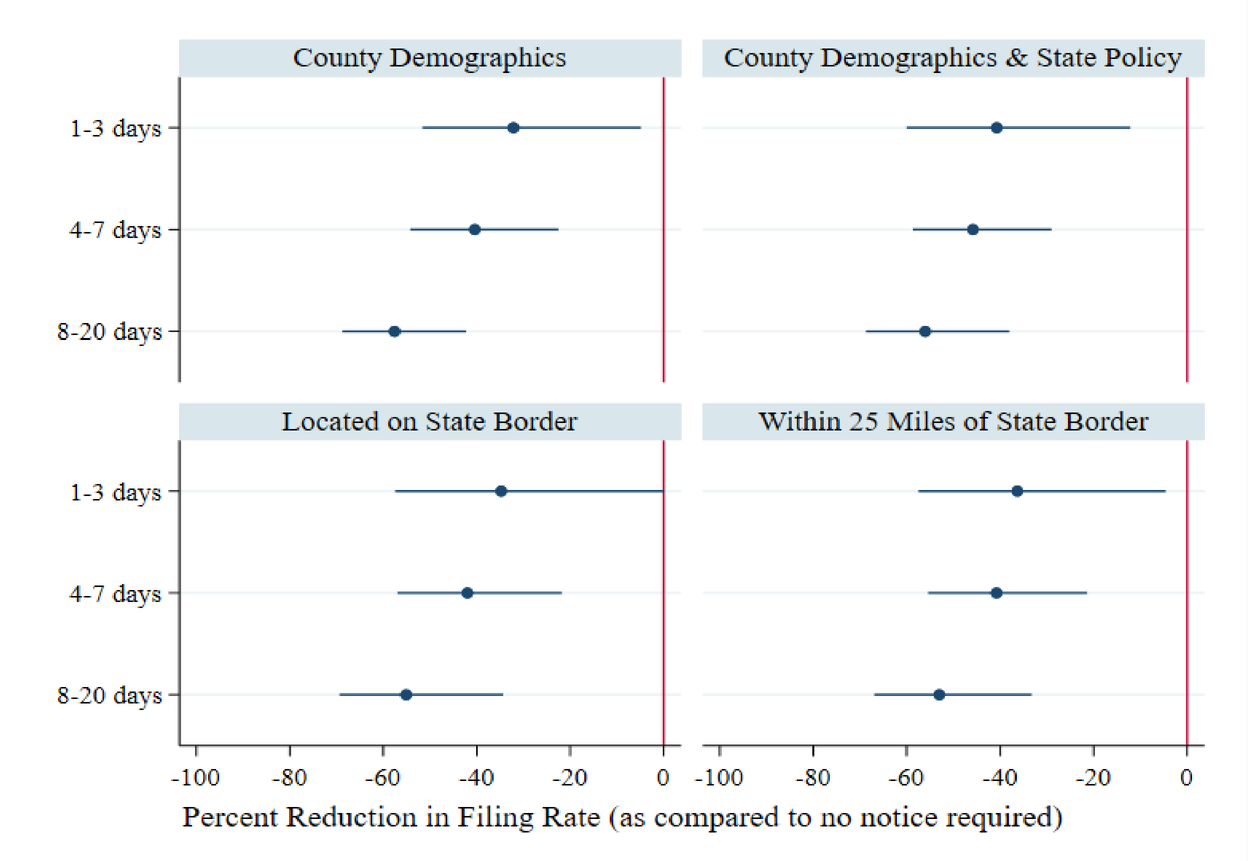

Fig. S32. Association between minimum number of days tenant can be late on rent before receiving an eviction filing and eviction filing rates

Estimated models analogous to those presented in Fig. 4.

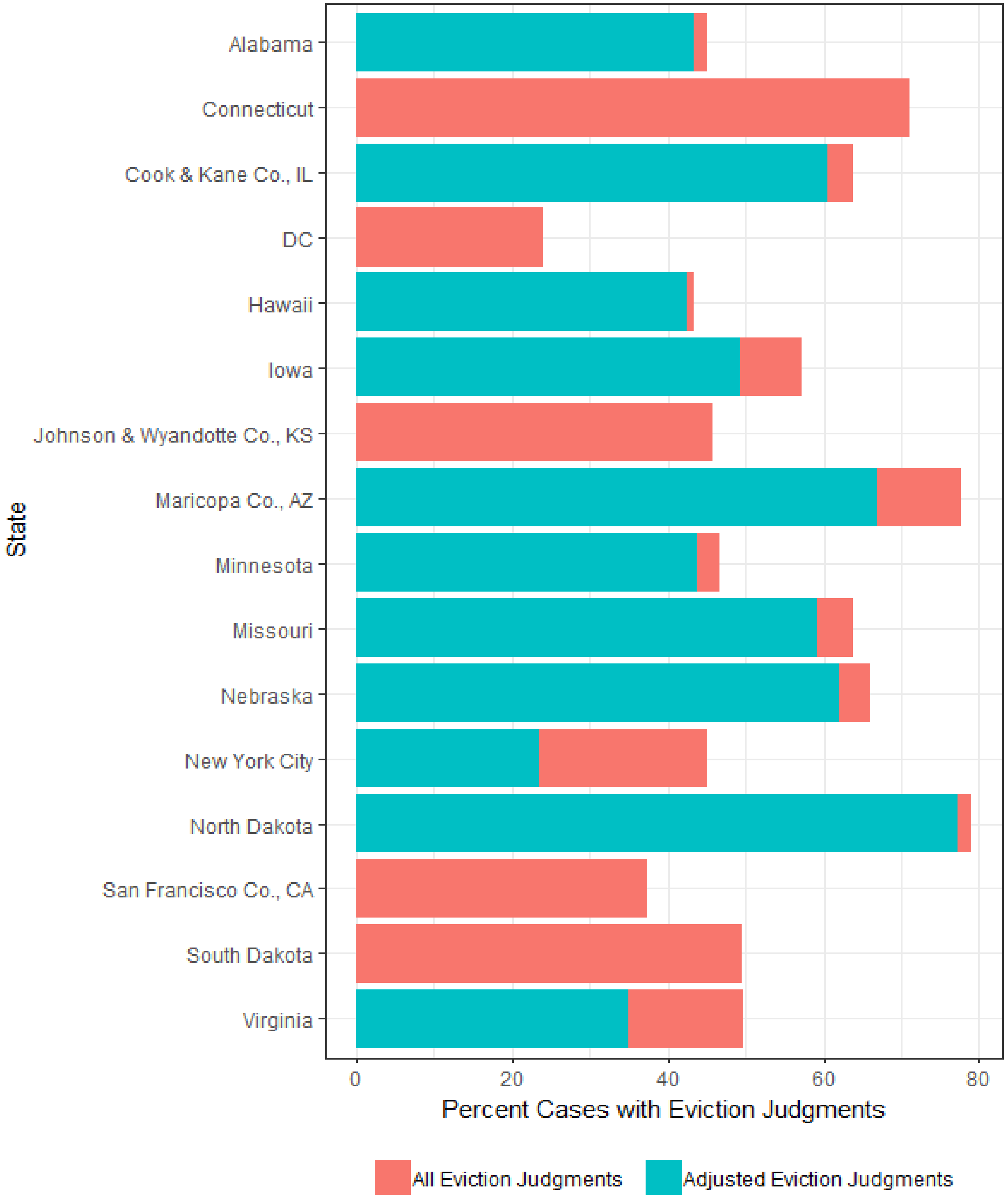

Fig. S33. Average rates of filings ending in eviction judgments, by state

Adjusted eviction judgment rates account for judgments on non-final filings against the same household. Adjusted eviction judgment rates cannot be calculated for San Francisco County, California; Connecticut; DC; Johnson and Wyandotte Counties, Kansas; and South Dakota due to missing name and address information.

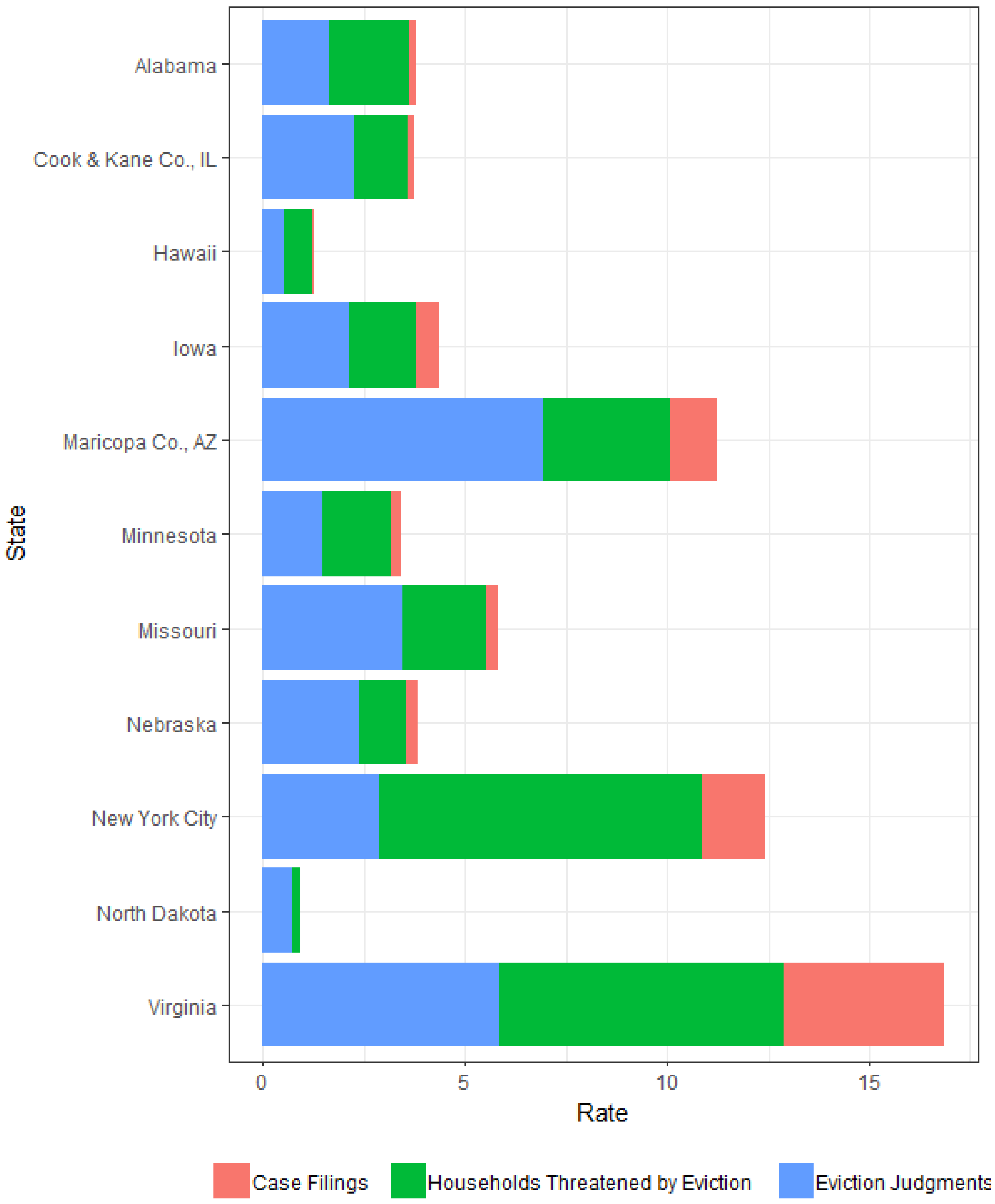

Fig. S34. Case filing, households threatened with eviction, and adjusted eviction judgment rates, by state

Rates calculated using total renting households as denominator and averaged across years.

10 Supplementary References and Notes

1. Arkansas is the only state that also allows eviction complaints to be filed in criminal courts as "Failure to Vacate" cases. For more information, see L. Foster, The Hands of the State: The Failure to Vacate Statute and Residential Tenants' Rights in Arkansas. **36**, 57.

2. Kansas Supreme Court, *Public Access to District Court Electronic Case Records* (2016).

3. D. Immergluck, J. Ernsthausen, S. Earl, A. Powell, Evictions, large owners, and serial filings: Findings from Atlanta. *Housing Studies* **35**, 1–22 (2019).

4. P. M. Garboden, E. Rosen, Serial filing: How landlords use the threat of eviction. *City & Community* **18**, 638–661 (2019).

5. This included data files received from courts in Alabama, Connecticut, Hawaii, Indiana, Missouri, New York, Oregon, and Philadelphia County.

6. Neither property nor defendant addresses were included in the data files received for DC, South Dakota, and Johnson & Wyandotte Counties in Kansas. Court data for Virginia included only the city name, state abbreviation, and zip code for defendant addresses, not the street address information. Court data for Orleans Parish, Louisiana included addresses for defendants and plaintiffs, but no information regarding which address corresponded to which party. For this reason, defendant addresses could not be consistently identified and assigned to cases.

7. We did not do this for the Philadelphia County court data. The addresses in the original data we received had already been cleaned and normalized using the Google Maps Geocoding API. Nearly all the cases (99%) were assigned a recognizable address.

8. ZCTAs do not align perfectly with USPS zip codes. ZCTAs are used for estimation of demographic statistics, as opposed to location identification for mail delivery, and reflect other areal unit boundaries (e.g., Census blocks) that are not considered in USPS assignment or realignment. ZCTAs may also include historical zip codes as well as secondary city names associated with a zip code.

9. We performed checks during the matching process to ensure that a 0.70 similarity score provided a reasonable basis for this assumption. This score is conservative and misses some of the more egregious errors in spelling of city names and significantly shortened abbreviations.

10. We also compared city names and states abbreviations in cases where a match could not be found for a city, state, and zip code combination. We used this measure to confirm the existence of a city within a state. Of the addresses that were not matched to a standardized city name, state, and zip code, 72.2% registered a valid city and state combination.

11. M. Desmond, A. Gromis, L. Edmonds, J. Hendrickson, K. Krywokulski, L. Leung, A. Porton, "Eviction Lab Methodology Report: Version 1.1.0" (Version 1.1.0, Princeton University, Princeton, NJ, 2018), (available at www.evictionlab.org/methods).

12. We omitted criterion (c) as the differences occurred in the street portion of the address rather than the city name or zip code. We used the remaining three criteria—(a), (b), and (d)—in that order.

13. Approximately 75% of commercial cases in the proprietary data were identified using this keyword method.

14. T. Enamorado, B. Fifield, K. Imai, Using a Probabilistic Model to Assist Merging of Large-Scale Administrative Records. *American Political Science Review*. **113**, 1–19 (2019).

15. M. A. Jaro, Advances in Record-Linkage Methodology as Applied to Matching the 1985 Census of Tampa, Florida. *Journal of the American Statistical Association*. **84**, 414–420 (1989).

16. W. E. Winkler, String Comparator Metrics and Enhanced Decision Rules in the Fellegi-Sunter Model of Record Linkage. *In JSM Proceedings, Survey Research Methods Section* (American Statistical Association, Alexandria, VA, 1990; pp. 354–359).

17. C. Geyer, R Package Pooh (University of Minnesota, 2017; https://cran.r-project.org/web/packages/pooh/pooh.pdf), *Partial Orders and Relations*.

18. S. Manson, J. Schroeder, D. Van Riper, S. Ruggles, *IPUMS National Historical Geographic Information System: Version 13.0 [Database].* (University of Minnesota, Minneapolis, 2018; http://doi.org/10.18128/D050.V13.0).

19. Missouri Census Data Center, *Geocorr: Geographic Correspondence Engine* (University of Missouri Office of Social and Economic Data Analysis, Missouri, 2014; http://mcdc.missouri.edu/applications/geocorr2014.html).

20. We also tested percent households (rather than population) residing in urban areas, but the results did not differ substantially.

21. Some states require the eviction notice to be served by the sheriff. In these states, the total case filing fee includes both the standard filing fee as well as the sheriff's fee to serve the notice.

22. BRB Publications, LLC, "Public Record Research System – PRRS" (2018), (available at www.brbpublications.com).

23. D. D. Ingram, S. J. Franco, NCHS Urban-Rural Classification Scheme for Counties. National Center for Health Statistics. *Vital Health Statistics*. **2** (2014).

24. Using these guidelines: https://www2.census.gov/geo/pdfs/maps-data/maps/reference/ us_regdiv.pdf.

25. We imposed the additional requirement that this 50% change represent a difference of at least ten court cases to prevent over-flagging of areas with low case volume.

26. S. E. Spielman, D. Folch, N. Nagle, Patterns and causes of uncertainty in the American Community Survey. *Applied Geography*. **46**, 147–157 (2014).

27. A. Gelman. Prior distributions for variance parameters in hierarchical models (comment on article by Browne and Draper). *Bayesian analysis*, **1**, 515-534 (2006).

28. Stan Development Team, *Stan Modeling Language Users Guide and Reference Manual* (2018; http://mc-stan.org).

29. We included the additional constraint that proprietary data coverage not exceed 130% of the filing volume reported in the court-issued data. Coverage in excess of 100% indicated that the proprietary data reported more case filings than the court-issued data. There were two primary reasons that the proprietary data may have exceeded court-issued filing counts. First, the proprietary data included all landlord-tenant cases while some court-issued data contained only eviction-specific cases. Second, some cases in the proprietary data had to be dated by judgment action rather than case filing (Section 1.3.11), which may have resulted in a few cases in these data being assigned to the subsequent calendar year. Over-coverage in the proprietary data was relatively uncommon, occurring in only 6.6% of county-years. When it did occur, the over-coverage tended to be minimal, with proprietary data coverage exceeding court-issued counts by more than 130% in only 1.9% of all county-years.

30. Public Justice Center, "Justice Diverted: How Renters in Baltimore Are Processed in the Baltimore City Rent Court" (Public Justice Center, Baltimore, MD, 2015).

31. C. Hartman, D. Robinson, Evictions: The hidden housing problem. *Housing Policy Debate* **14**, 461–501 (2003).

32. *Michie Annotated Code of Maryland*.

33. There is a limit to the number of times within a 12-month period that the tenant may "pay to stay" before the landlord can elect to refuse the past-due payment and follow through with the eviction (*32*).

34. The Maryland Judiciary reports can be accessed at: https://mdcourts.gov/publications/annualreports

35. M. E. Hatch, Statutory protection for renters: Classification of state landlord–tenant policy approaches. *Housing Policy Debate* **27**, 98–119 (2017).

36. Legal Services Corporation, "Eviction Laws Database: State/Territory Dataset." (Center for Public Health Law Research at Temple University's Beasley School of Law for Legal Services Corporation, 2021).

37. K. M. Pence, Foreclosing on Opportunity: State Laws and Mortgage Credit. *The Review of Economics and Statistics* **88**, 177–182 (2006).

38. T. J. Holmes, The Effect of State Policies on the Location of Manufacturing: Evidence from State Borders. *Journal of Political Economy*. **106**, 667-705 (1998).

39. R. Kleysteuber, Tenant screening thirty years later: A statutory proposal to protect public records note. *Yale L.J.* **116**, 1344–1388 (2006).

40. If adjusting for non-final cases against households, 8 states and 8 counties from which we can calculate this measure recorded an average of 229,986 eviction judgments on filings annually. This would be an average rate of 50.6% of filings ending in eviction judgments.

41. Changed Locks: Getting Evicted in Wyandotte. *Flatland KC* (2018), (https://www.flatlandkc.org/public-works/level-foundation/reporting/changed-locks-evicted-wyandotte/).
